# Biophysical insights into a cryptic ligand site in the hydrophobic core of human PCNA

**DOI:** 10.1016/j.bpj.2026.05.027

**Published:** 2026-05-20

**Authors:** Alexis N. Dispensa, Sharon N. Greenwood, Jason Yang, Daniel E. Logatto, James Fusco, E. Railey White, Roderic G. Eckenhoff, Zhiwei Liu, Brian P. Weiser

**Affiliations:** 1 Department of Cell & Molecular Biology, Rowan-Virtua School of Osteopathic Medicine, Rowan University, Stratford, NJ, USA; 2 Department of Cell & Molecular Biology, Rowan-Virtua School of Translational Biomedical Engineering & Sciences, Rowan University, Stratford, NJ, USA; 3 Internal Medicine, Lehigh Valley Health Network, Allentown, PA, USA; 4 Department of Anesthesiology and Perioperative Medicine, Thomas Jefferson University Hospital, Philadelphia, PA, USA; 5 Department of Anesthesiology & Critical Care, University of Pennsylvania Perelman School of Medicine, Philadelphia, PA, USA; 6 Department of Chemistry & Biochemistry, Rowan University College of Science & Mathematics, Glassboro, NJ, USA

## Abstract

Proteins contain small pockets that form due to imperfections in residue packing or the rotational and conformational movement of amino acids. In this work, we used the fluorescent probe 1-aminoanthracene (AMA) to detect a small ligand pocket in the hydrophobic core of human proliferating cell nuclear antigen (PCNA), which is a critical protein for DNA replication and repair. Fluorescence measurements of AMA reported that the core of PCNA had a dielectric constant (ε) of 4, which was very apolar and similar to cyclohexane (ε = 2). Protein mutagenesis, photoaffinity labeling, and molecular dynamics simulations localized the binding site for AMA next to PCNA residues L90 and L101, which also interacted with general anesthetics (sevoflurane and propofol). The ligand binding site was cryptic, i.e., it formed transiently and was only detectable in certain structural states of PCNA. Ligand binding to the cryptic site on PCNA structurally stabilized the trimeric protein and reduced its ability to disassemble and reassemble its subunits. Thus, the cryptic site in PCNA’s core serves to destabilize the assembled protein and promotes structural and oligomeric flexibility. Finally, the hydrophobic site is widely conserved among homologous β clamp proteins with a similar fold as PCNA. This work highlights how small fluorescent probes can reveal ligand sites within proteins, defines the chemical features of protein hydrophobic cores, and introduces a novel approach to modulate the oligomeric stability of PCNA.

## Graphical abstract

**Figure:**
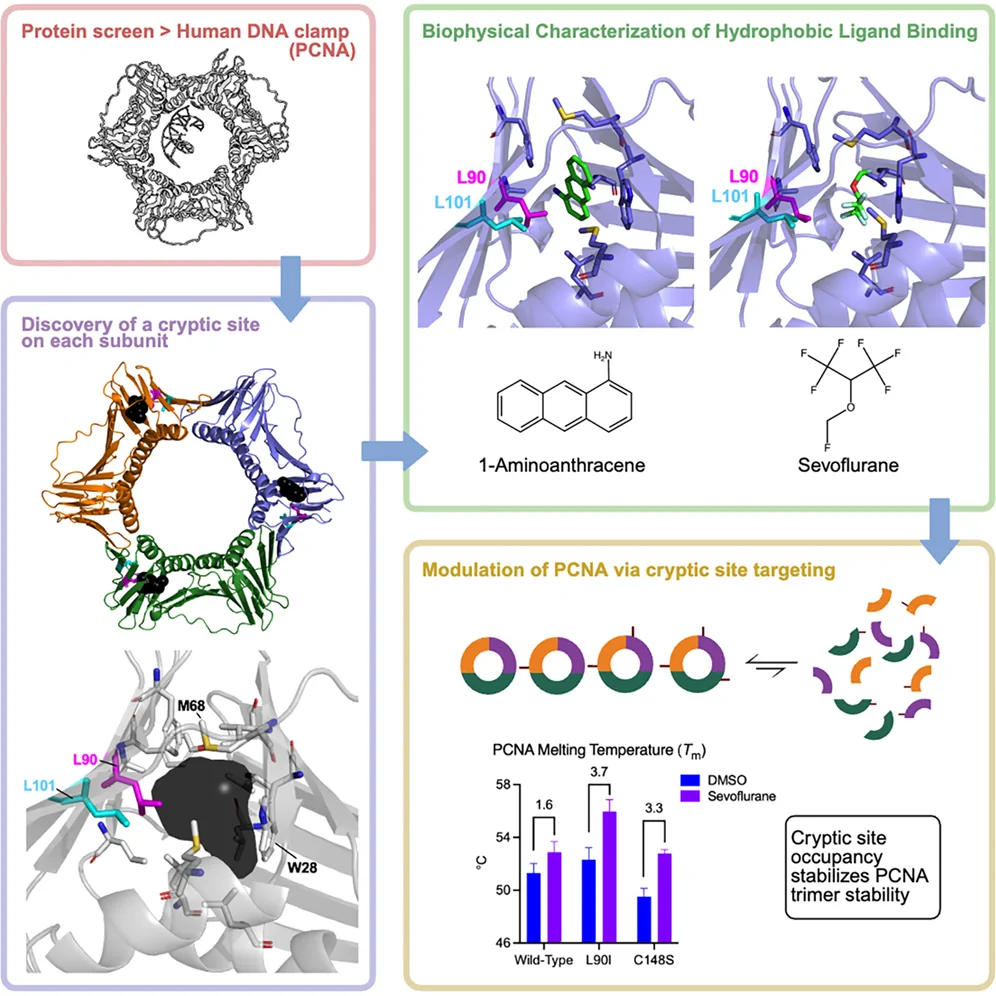

## Significance

Cryptic binding sites for small molecules appear and disappear on proteins as they sample different structural states. Many of these sites can be targeted for therapeutic purposes but are not well understood. In addition to surface binding sites, ligand pockets also form internally where hydrophobic residues are not tightly packed together. We used a fluorescent probe to detect these hydrophobic pockets on proteins. After a small screen, we identified a ligand binding site in the hydrophobic core of a human protein called PCNA. Clinically used general anesthetics also interacted with the site. We characterized physical, chemical, and functional features of the new ligand site on PCNA, which influenced the stability of the trimeric protein.

## Introduction

We have long understood that the interior of soluble proteins consists of hydrophobic residues that pack together to avoid the aqueous environment and help stabilize protein structure. However, due to imperfections in the packing of residue side chains, it was reported that on average ∼44% of the protein core is actually unoccupied volume, which can contribute to the formation of various cavities or tunnels inside proteins.^1^ These spaces affect protein dynamics and conformational flexibility and can play critical roles in allosteric regulation or ligand migration.^2^^,^^3^^,^^4^ These spaces may be transient or “cryptic,” appearing only in certain structural states due to protein dynamics, including small or large atomic movements such as side chain rotations or conformational changes.^5^^,^^6^^,^^7^^,^^8^ Cryptic and hydrophobic sites can be targeted with small molecules for therapeutic purposes, but there is much to understand about the formation of cryptic cavities, their persistence, and their physicochemical features.^5^^,^^6^^,^^9^

Techniques such as X-ray crystallography, NMR, and molecular dynamics (MD) simulations have allowed us to directly observe cavities in the protein core and calculate parameters such as their size, geometry, and polarizability.^3^^,^^6^^,^^7^^,^^10^^,^^11^^,^^12^^,^^13^ Detecting these spaces with molecular probes is challenging because of their small sizes, structural plasticity, and hydrophobicity, which limit well-defined molecular contacts in the sites and reduce opportunities for strong ligand-protein interactions such as hydrogen bonding. However, we note that an entire class of lipophilic drugs—injectable and inhalable general anesthetics—bind weakly to hydrophobic protein sites (μM to mM *K*_d_ values), yet anesthetics can be surprisingly selective for their protein targets.^14^^,^^15^^,^^16^ General anesthetics are small (<250 Da) and lipophilic compared with conventional therapeutics, but even these molecules are larger than the minimal chemical probes, such as benzene or xenon (itself an anesthetic), which are used to explore smaller internal protein cavities.^3^^,^^10^^,^^17^^,^^18^^,^^19^

In this work, we used fluorescence to probe hydrophobic protein sites using 1-aminoanthracene (AMA), which has fluorescence that “turns on” when the molecule is in apolar environments.^20^^,^^21^^,^^22^^,^^23^ Low molecular weight fluorophores such as AMA can produce various changes in quantum yield and solvatochromism in response to their molecular surroundings, solvent polarity, or pH.^21^^,^^24^^,^^25^^,^^26^ AMA also has general anesthetic properties^16^^,^^22^ and has been reported to bind over a dozen structurally diverse proteins (Table S1). Known AMA binding sites on proteins frequently overlap with regions targeted by other anesthetics, lipids, and small lipophilic molecules such as pheromones and odorants (Table S1). Several AMA binding sites are known to be druggable and correspond to pockets or cavities bound by clinically used compounds.^16^^,^^22^^,^^27^^,^^28^^,^^29^^,^^30^

We used AMA to discover a novel cryptic site in the hydrophobic core of human proliferating cell nuclear antigen (PCNA), which is a ring-shaped protein composed of three identical subunits. In the cell, PCNA encircles dsDNA and acts as a scaffold for localizing eukaryotic replication and repair proteins to the replication fork.^31^ PCNA is part of a large, evolutionarily conserved family of sliding clamps that are structurally and functionally similar, including prokaryotic β clamps, which enable processivity for proteins on DNA. PCNA is also a drug target for cancers because of its critical importance for cellular DNA replication.^32^^,^^33^ We characterized structural and chemical features of the cryptic site on PCNA and identified other ligands that bind to the site (e.g., general anesthetics). The cryptic site exists as empty space inside PCNA’s core that weakly destabilizes the trimeric protein and promotes its disassembly and reassembly, which spontaneously unloads PCNA from dsDNA.^34^^,^^35^ Our work supports the idea that open cavities or space within the protein core inherently affect structural stability and can influence processes such as oligomerization or exchange in multi-subunit assemblies.

## Materials and methods

### Chemicals

**Figure 1:**
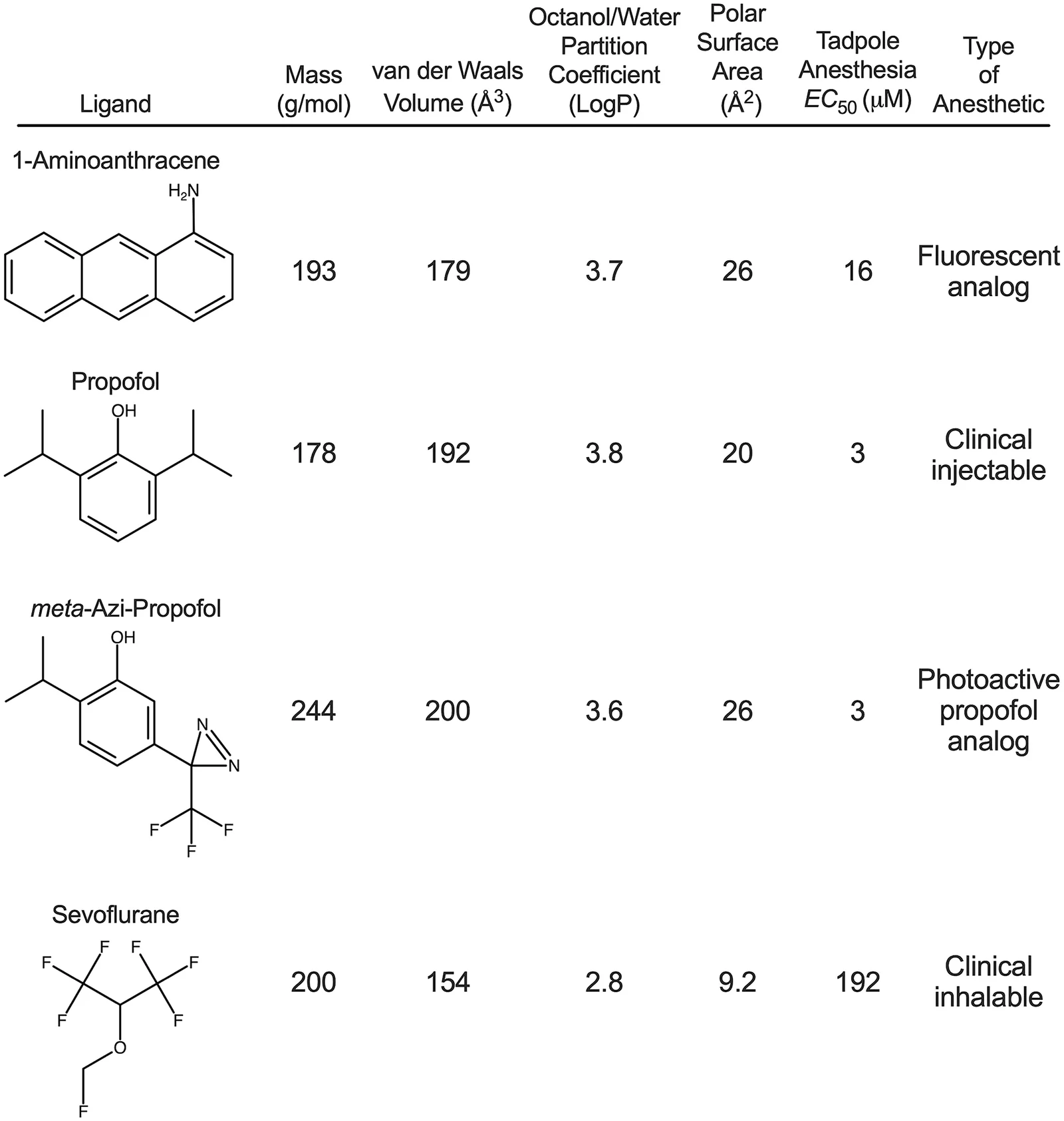
Chemical structures and properties of anesthetic compounds used in this study. Values in the table were previously reported.^16^^,^^22^^,^^36^^,^^37^^,^^38^

Chemical and pharmacologic properties of relevant small molecules can be found in Figure 1. AMA was purchased from TCI America (Portland, Oregon), and propofol (2,6-diisopropylphenol) was purchased from Alfa Aesar (Ward Hill, Massachusetts). The synthesis of *meta-*azi-propofol (AziP*m*) has previously been reported.^36^ Sevoflurane (fluoromethyl-1,1,1,3,3,3-hexafluoroisopropyl ether) was purchased from TCI America.

### Proteins

Proteins that were purchased included human apolipoprotein E (ApoE) (amino acids 19–317 with a C-terminal 6xHis tag) (Bon Opus Biosciences, Shawnee, Kansas); pyruvate kinase from rabbit muscle (Roche, Basel, Switzerland); lactate dehydrogenase from rabbit muscle (Roche); *E*. *coli* single-stranded binding protein (Qiagen, Hilden, Germany); thermolysin (Promega, Madison, Wisconsin); streptavidin (Promega); and SpyCatcher3 (Bio-Rad, Hercules, California). Human ΔN555-Replication factor C (RFC), which was composed of five subunits, was a generous gift from Dr. Brian Kelch (University of Massachusetts).^39^ 6xHis-PCNA was expressed and purified as described previously^40^; when relevant, standard QuikChange/site-directed mutagenesis was used to introduce mutations onto the plasmid encoding the protein. Expression and purification of full-length human uracil DNA glycosylase (UNG2) and *S*. *cerevisiae* ubiquitin-like protease 1 catalytic domain (amino acids 403–621) have been reported.^40^ Human sirtuin isoform 2 (SIRT2) catalytic domain (amino acids 34–356) and human sirtuin isoform 3 (amino acids 120–399 with an N-terminal 6xHis tag) were also expressed and purified as previously described.^27^^,^^41^

Purified replication protein A-70N contained amino acids 1–120 of the human RPA1 gene with an N-terminal 8xHis tag. We originally inserted the 8xHis tag into the p11d-tRPA plasmid that encodes RPA1 along with other RPA subunits.^42^ Then, we amplified the 8xHis tag and residues 1–120 flanked by NdeI and NotI sites for standard restriction enzyme cloning into a pET21a vector.^40^ The tagged replication protein A-70N construct was expressed and purified with standard procedures for Ni^2+^ chromatography followed by size exclusion chromatography. The sequences of all plasmids used for protein expression were verified with Sanger sequencing.

Human FAM72a was a tag-free recombinant protein that was produced in the following manner. The human FAM72a gene was obtained from a plasmid that was a generous gift from Dr. Jessica Stewart (University of North Carolina). This gene was amplified and inserted into a pET21a vector using standard cloning methods such that the following construct was encoded: 8xHis-SUMO-FAM72a with a Gly-Ser-Gly-Ser-Gly linker between the 8xHis tag and SUMO domain.^40^ The protein was expressed and purified with standard procedures, which included removal of the 8xHis-SUMO tag with the SUMO protease during purification.^40^

Untagged human PCNA and SpyTag-His-PCNA were expressed from the same pET21a vector as 6xHis-PCNA after truncating or enlarging the gene with site-directed mutagenesis.^40^ Untagged PCNA was expressed as the wild-type protein sequence (UniProt: P12004). SpyTag-His-PCNA had the SpyTag-containing sequence MAHIVMVDAYKPTKGG upstream of an N-terminal 6xHis tag. After standard protein expression conditions,^40^ untagged PCNA and SpyTag-His-PCNA were purified in the following manner. Cell pellets were resuspended in lysis buffer (20 mM Tris-HCl, pH 7.5, 30 mM NaCl, 1 mM DTT, protease inhibitor cocktail) and lysed with a microfluidizer. After centrifugation to remove cell debris, the lysate was flowed through SP-sepharose resin (Cytiva, Marlborough, Massachusetts), and then PCNA in the flow through was captured on Q-sepharose resin (Cytiva). Protein was eluted with a stepwise gradient of 100–650 mM NaCl dissolved in lysis buffer. PCNA-containing fractions were pooled and diluted 1:5 in Buffer A (20 mM Tris-HCl, pH 7.5, 30 mM NaCl, 1 mM DTT, 5% glycerol). The protein was further purified by fast protein liquid chromatography by loading the material onto a Bio-Rad ENrich Q-column and eluting with a linear gradient of Buffer B (20 mM Tris-HCl, pH 7.5, 1 M NaCl, 1 mM DTT, 5% glycerol).

### Fluorescence assays with protein and AMA

All assays were performed at 23°C in PBS with 1 mM DTT as described elsewhere.^27^ A 200-μL quartz microcuvette (3 mm path length) was used with a Horiba Fluoromax-4 fluorometer for all measurements. To measure background spectra, protein samples were diluted in PBS and excited with 410 nm while an emission scan was collected from 420 to 700 nm. AMA was then added to achieve a final concentration of 100 nM, and the sample was gently mixed by pipetting before repeating the fluorescence scan. Background spectra from protein alone were subtracted from the spectra of protein with AMA, and the resulting background-subtracted counts per second were plotted against emission wavelength.

To determine binding affinities between AMA and proteins, we measured the fluorescence of 100 nM AMA in the presence of increasing protein concentrations while subtracting protein-only background fluorescence as described above. The peak fluorescence value at each protein concentration was extracted from the AMA spectra, and then, this value was normalized by dividing it by the maximum fluorescence observed in each assay. Normalized fluorescence was plotted as *Y* values against the protein concentration as *X* values. A curve was fit to the data to determine the *K*_d_ for AMA interacting with the protein using the quadratic binding equation:

$$Y=Y_{\min}-\left[\frac{Y_{\min}-Y_{\max}}{2\left[AMA\right]}\right]\times \left[b-\sqrt{b^{2}-4\;\left[PROTEIN\right]\left[AMA\right]}\right]$$

$$b=K_{d}+\left[PROTEIN\right]+\left[AMA\right]$$

Here, *Y*_*min*_ and *Y*_*max*_ were the minimum and maximum normalized intensities, [AMA] was the total AMA concentration, [PROTEIN] was the protein concentration, and *K*_d_ was the protein concentration at which half-maximal saturation occurred.

For AMA/ligand competition assays, 10 μM PCNA was equilibrated with the indicated concentration of competitor ligand (along with 0.5% DMSO as the vehicle), and a background emission scan was collected. AMA was then added to a final concentration of 100 nM, the sample was mixed by pipetting, and the fluorescence scan was repeated. Background spectra of PCNA with competitor ligand were subtracted from spectra with AMA as before. The fluorescence of AMA alone in the absence of protein was subtracted from all values because this was the theoretical minimum fluorescence that could occur in the competition assay. The fluorescence intensities were then normalized by dividing the values by the fluorescence of 10 μM PCNA with 100 nM AMA in the absence of competitor and multiplying by 100%. Normalized fluorescence intensities were plotted as *Y* values against the log_10_ of the competitor ligand concentration as *X* values, and the data were fit with a sigmoidal curve:

$$Y=Y_{\min}+\frac{Y_{\max}-Y_{\min}}{{1+10}^{n*\left(\log\left({IC}_{50}\right)-X\right)}}\text{,}$$

where *Y*_min_ and *Y*_max_ were set to 0 and 100, respectively, the *IC*_50_ was the *X* value at the halfway point between the two asymptotes, and *n* was the Hill slope. Under our assay conditions, the *IC*_50_ values for the displacement of AMA from PCNA proteins using sevoflurane/propofol were essentially equal to the *K*_d_ values for the interaction of sevoflurane/propofol with PCNA.^43^ Fractional occupancies of PCNA bound to sevoflurane in specific assays were estimated using the equation

$$\%Occupancy=\frac{\left[L\right]}{K_{d}+\left[L\right]}\times 100\text{,}$$

where [L] was the concentration of sevoflurane, and *K*_d_ was the affinity of PCNA for sevoflurane.

To evaluate the relationship between residue hydrophobicity in the cryptic site and the fluorescence of AMA, we considered how the cryptic site contained leucine residues at positions 90 and 101 in the wild-type protein. All of the PCNA variants that we tested retained one leucine at 90 or 101 along with a second amino acid for the mutation; therefore, we examined the effect of the second (nonleucine) residue that was at position 90 or 101. Hydrophobicity scores for that second residue were obtained from Monera et al. who derived a hydrophobic scaling system that was also used by Fpocket.^44^ The hydrophobicity scores for the second residues were plotted as *X* values against the peak emission wavelengths of AMA in the presence of the corresponding variants as *Y* values. A curve was fit to the data using the equation

$$Y=Y_{\min}+\left(Y_{0}-Y_{\min}\right)\times \exp\left(-K*X\right)\text{,}$$

where *Y*_min_ was the minimum *Y* value at the plateau, *Y*_0_ was the initial *Y* value at the lowest *X* values, and *K* was a constant controlling the transition from *Y*_0_ to *Y*_min_.

### Fluorescence measurements with AMA in solvents and calculation of protein dielectric constants

Background spectra of solvents were collected, and then, AMA (200 nM) was dissolved in the solvents and scanned with our fluorometer (excitation = 410 nm, emission = 420–700 nm). The background spectra were subtracted from the spectra of solvent with AMA. The peak emission wavelength of AMA in each solvent was then extracted from the background-subtracted spectra after smoothing the spectra using a second-order Savitzky-Golay polynomial filter (nine-point window) as implemented in GraphPad Prism v10.^45^ The peak emission wavelengths were plotted as *Y* values against the solvent dielectric constants (ε) as *X* values. A hyperbolic curve was fit to the data using

$$Y=Y_{\min}+\frac{\left(Y_{\max}-Y_{\min}\right)*X}{K+X}\text{,}$$

where *Y*_*min*_ and *Y*_*max*_ were the minimum and maximum emission wavelengths (nm) of AMA, respectively, and *K* was the solvent polarity at which AMA’s emission wavelength was halfway between its minimum and maximum. Subsequently, we used this as a standard curve to determine the dielectric environment of AMA binding sites by interpolation using the peak emission wavelength of AMA in the presence of individual proteins or DOPC; we also used the Savitzky-Golay smoothing method in determining peak emission wavelengths for these spectra.

### PCNA photolabeling and mass spectrometry

Purified 6xHis-PCNA was diluted in PBS with 1 mM DTT, pH 7.4, to a final concentration of 0.47 mg/mL (30 μM PCNA monomer). AziP*m* was added to a final concentration of 10, 20, or 50 μM on ice for 25 min in the dark; every sample also contained 1% DMSO from preparing AziP*m* stock solutions. Samples were transferred to a 1-mm pathlength quartz cuvette and exposed to a 350-nm light source (Rayonet RPR-3500 lamp, Branford, Connecticut) for 25 min at a distance of <1 cm. For a control “sham” photolabeling experiment, 6xHis-PCNA was incubated with 1% DMSO and exposed to the same 350-nm light source without the addition of AziP*m*. After photolabeling, the protein was precipitated with acetone, resuspended in buffer containing DTT, and alkylated with iodoacetamide. 6xHis-PCNA was then digested in solution with trypsin, desalted with C18 StageTips,^46^ and then analyzed with an Orbitrap Elite Hybrid Ion Trap-Orbitrap Mass Spectrometer (MS) coupled to an Easy-nanoLC 1000 system. Spectra of PCNA peptides were analyzed with MaxQuant version 2.1.4.0.^47^ The analysis included the dynamic oxidation of methionine (+15.9949 m/z) and static alkylation of cysteine by iodoacetamide (+57.0215 m/z). PCNA amino acids were also searched for a possible mass adduct of +216.0762 m/z, which corresponded to a residue photolabeled with AziP*m*.^48^^,^^49^ Up to two missed trypsin cleavages were allowed in the searches. The spectra showing an AziP*m* adduct on PCNA peptides were then manually reviewed for confirmation. Using the human PCNA sequence from the UniProt database (UniProt: P12004), the following sequence coverages were obtained for the photolabeling conditions: 10 μM AziP*m*, 83.1%; 20 μM AziP*m*, 83.1%; 50 μM AziP*m*, 83.1%; DMSO only, 95.8%. Further details on the photolabeling and mass spectrometry methods can be found elsewhere.^50^

### MD simulations

1-μs MD simulations of wild-type PCNA and PCNA(L90I) were run using an available crystal structure as the starting structure (PDB: 1VYM).^51^ Independent 500-ns simulations of PCNA and PCNA(L90I) were run using an AlphaFold model as the starting structure, downloaded from AlphaFold Protein Structure Database.^52^ MD simulations were performed using the Amber suite of biomolecular simulation programs (version 22)^53^ with the ff19SB force field for protein^54^ and protonation states assigned according to pH 7.0. The system was solvated in a cubic box of OPC water molecules with periodic boundary conditions,^55^ ensuring a minimum distance of 10 Å from the protein edge to the box boundary. To mimic physiological ionic strength, K^+^ and Cl^−^ ions were added to neutralize the system and achieve a final salt concentration of 150 mM. The system was equilibrated following a standard protocol: (1) relaxing (by energy minimization) solvent and ions with protein constrained, (2) relaxing the entire system to remove bad contacts in initial structures, (3) heating to 300 K gradually, and (4) equilibration under constant pressure (NPT) at 1 atm for 1 ns to ensure density matching that of an aqueous environment. Production simulations were run in the NVT ensemble. Long-range electrostatics were treated using the particle mesh Ewald method. MD trajectories were saved every 10 ps for downstream analysis. All visualizations were generated with PyMol (v3.1.5.1) or VMD.^56^

### Detection and analysis of cryptic binding sites

Putative ligand-binding pockets on human PCNA were detected using the Fpocket suite (version 4.0) using default parameters.^57^ The pocket characteristics of PCNA and PCNA(L90I) during their MD trajectories were analyzed using MDpocket.^58^ Specifically, the structure of PCNA at every 10 ns during the simulation was aligned to the starting (crystallographic or AlphaFold) structure based on root mean square fit of protein backbone atoms. Then, grid-based pocket detection identified all cavities along the trajectory. The density grid (.dx file) was loaded into VMD with the starting structure, and the iso-value was set to the lowest level that produced a pocket within 5 Å of residues 90 and 101 (i.e., we localized the analysis to the pocket at residues 90 and 101) (actual iso-values ranged from 0.1 to 0.5, depending on the simulation). The alpha spheres composing the site were selected, and MDpocket was re-run on the simulation using this pocket definition to calculate features of our site of interest, such as pocket volume. Frames containing pockets that overlap with alpha spheres from our selected sites, and which have specific features such as volumes large enough to accommodate our ligands, could then be identified and extracted as .pdb files. Detailed maps (.pdb files and .dx files) showing the shapes of the ligand site can be found in the Zenodo data sets that accompany this work.

Pockets on ∼100 structures of human and yeast PCNA, as well as various prokaryotic β clamps, were also detected using Fpocket with default parameters.^57^ The protein structures with the output pockets were aligned to human PCNA (PDB: 1VYM) to identify internal hydrophobic sites on the surveyed proteins that were positioned analogously to human L90/L101. AlphaFold3 models of human PCNA and PCNA(L90I) were generated using AlphaFold Server, which defaults to provide five models per run, all of which were analyzed.^59^

### Ligand docking for MD simulations

Ligand structures were prepared with LigPrep to generate low-energy 3D conformers with proper protonation states at physiological pH (7.0 ± 0.5) using Epik.^60^^,^^61^ All possible tautomers and stereoisomers were generated, and energy minimization was performed using the OPLS4 force field.^62^ Protein structures for docking were extracted from the MD simulations where the cryptic sites were open. Receptor grids were generated using Glide^63^^,^^64^^,^^65^ centered on the putative binding site, with the grid box size adjusted to fully encompass the pockets identified by MDpocket. Standard Precision (SP) Glide docking was performed with flexible ligand sampling, and the top-ranked poses were analyzed based on GlideScore and predicted binding modes. Key interactions were visualized and evaluated in Maestro (Schrödinger Release 2024-4), and the best-scoring poses were used as starting structures for subsequent MD simulations.

### PCNA thermostability measurements with fluorescence

PCNA unfolding was assessed by measuring intrinsic tryptophan fluorescence as a function of temperature in the presence and absence of sevoflurane.^66^ Experiments were conducted in PBS with PCNA at a final concentration of 3 μM. Sevoflurane was added from a freshly prepared stock in DMSO to a final concentration of 2.5 mM, and the same volume of DMSO (1%) was added to control samples. Samples (3.8 mL) were loaded into a synthetic quartz cuvette, sealed tightly and then subjected to a controlled temperature ramp from 25°C to 70°C with 3-min holds every 2.5°C increment using a Horiba Fluoromax-3 instrument equipped with a Peltier temperature controller. Tryptophan fluorescence was monitored with excitation at 280 nm and emission at 325 nm. For every experiment, fluorescence values were normalized such that the initial fluorescence of the folded protein at 25°C was set to 100, and the final fluorescence at 70°C was set to 0. The unfolding transition midpoint (*T*_m_) was determined by plotting the normalized fluorescence intensities as *Y* values against the temperature as *X* values and fitting the data with a sigmoidal equation:

$$Y=Y_{\min}+\frac{Y_{\max}-Y_{\min}}{1+{\left(\frac{T_{m}}{X}\right)}^{n}}\text{,}$$

where *Y*_min_ and *Y*_max_ were the normalized fluorescence intensities at the lower and upper asymptotes, *T*_m_ was the *X* value at the halfway point between the two asymptotes, and *n* was the Hill slope.

### PCNA subunit exchange

Subunit exchange between PCNA trimers in the absence and presence of sevoflurane was measured as follows. Reactions (600 μL) were initiated by mixing equimolar concentrations (10 μM each) of untagged PCNA and SpyT-His-PCNA in Buffer C (10 mM Tris-HCl, pH 7.5, 100 mM NaCl, 1 mM TCEP). At defined time points (0.33, 15, 30, 60, 90, 120 min), 100-μL aliquots were withdrawn, applied to pre-equilibrated Ni^2+^-NTA resin in a microcentrifuge tube, and gently mixed by pipetting to allow SpyT-His-PCNA to bind the resin. These were then centrifuged at 15,000 RPM for 1 min, washed with Buffer C, and then, SpyT-His-PCNA-containing trimers were eluted with Buffer D (10 mM Tris-HCl, pH 7.5, 100 mM NaCl, 1 mM TCEP, 300 mM imidazole). SpyCatcher3 (20 μM) (referred to as simply “SpyCatcher” in this work) was added to the eluate for 3 h to allow for its covalent reaction to the SpyTag of SpyT-His-PCNA. Samples were then separated by SDS-PAGE to resolve untagged PCNA from the SpyCatcher-SpyT-His-PCNA complex. After staining with Coomassie, band intensities for untagged PCNA and SpyCatcher-SpyT-His-PCNA were quantified using FIJI/ImageJ.^67^

To have the band intensities of untagged PCNA and SpyCatcher-SpyT-His-PCNA reflect their relative molar amounts, we had to correct for the darker staining of the latter protein per mol because of its additional mass. We multiplied the intensity of untagged PCNA by a correction factor (*C*_f_). The *C*_f_ was determined by analyzing known, molar equivalent amounts of untagged PCNA and SpyCatcher-SpyT-His-PCNA (i.e., “tagged PCNA”) on a Coomassie-stained SDS-PAGE gel and using the ratio

$$C_{f}=\frac{{Intensity}_{tagged\;PCNA}}{{Intensity}_{untagged\;PCNA}}\text{,}$$

where intensity values came from FIJI/ImageJ quantification. Thus, the relative molar amount of untagged/tagged PCNA subunits in each lane could be determined. The molar ratio of untagged/tagged PCNA subunits was plotted on the *Y* axis against time on the *X* axis, and the single-phase exponential data sets were fit with a curve using

$$Y=\left(Y_{\min}-Y_{\max}\right)*\exp\left(-k_{obs}*X\right)+Y_{\max}\text{,}$$

where *Y*_min_ was molar ratio at time 0 (which was constrained to 0), *Y*_max_ was molar ratio at the plateau at longer time points, and *k*_obs_ was a rate constant controlling the transition from *Y*_min_ to *Y*_max_.

If all of the untagged and tagged PCNA subunits distributed completely, evenly, and unhindered during the course of the assay, then the expected molar ratio of each trimeric species before pull-down with the Ni^2+^ resin would be as follows: trimers composed of three untagged subunits would represent 1/8 of the total population; those with two untagged and one tagged subunit, 3/8; those with one untagged and two tagged subunits, 3/8; and trimers with three tagged subunits, 1/8. Only the trimer composed entirely of untagged subunits will fail to bind to the Ni^2+^ resin, while the remaining species are retained. Consider the total number of subunits within the retained population along with the fraction of each species. One trimer containing three tagged subunits contributes 3 tagged subunits. Three trimers composed of one untagged and two tagged subunits contribute a total of 3 untagged and 6 tagged subunits. Three trimers composed of 2 untagged and 1 tagged subunit contribute a total of 6 untagged and 3 tagged subunits. The ideal untagged/tagged subunit ratio at equilibrium is therefore 9/12 or 3/4.

## Results

### Fluorescence measurements of AMA in various protein and chemical environments

**Figure 2:**
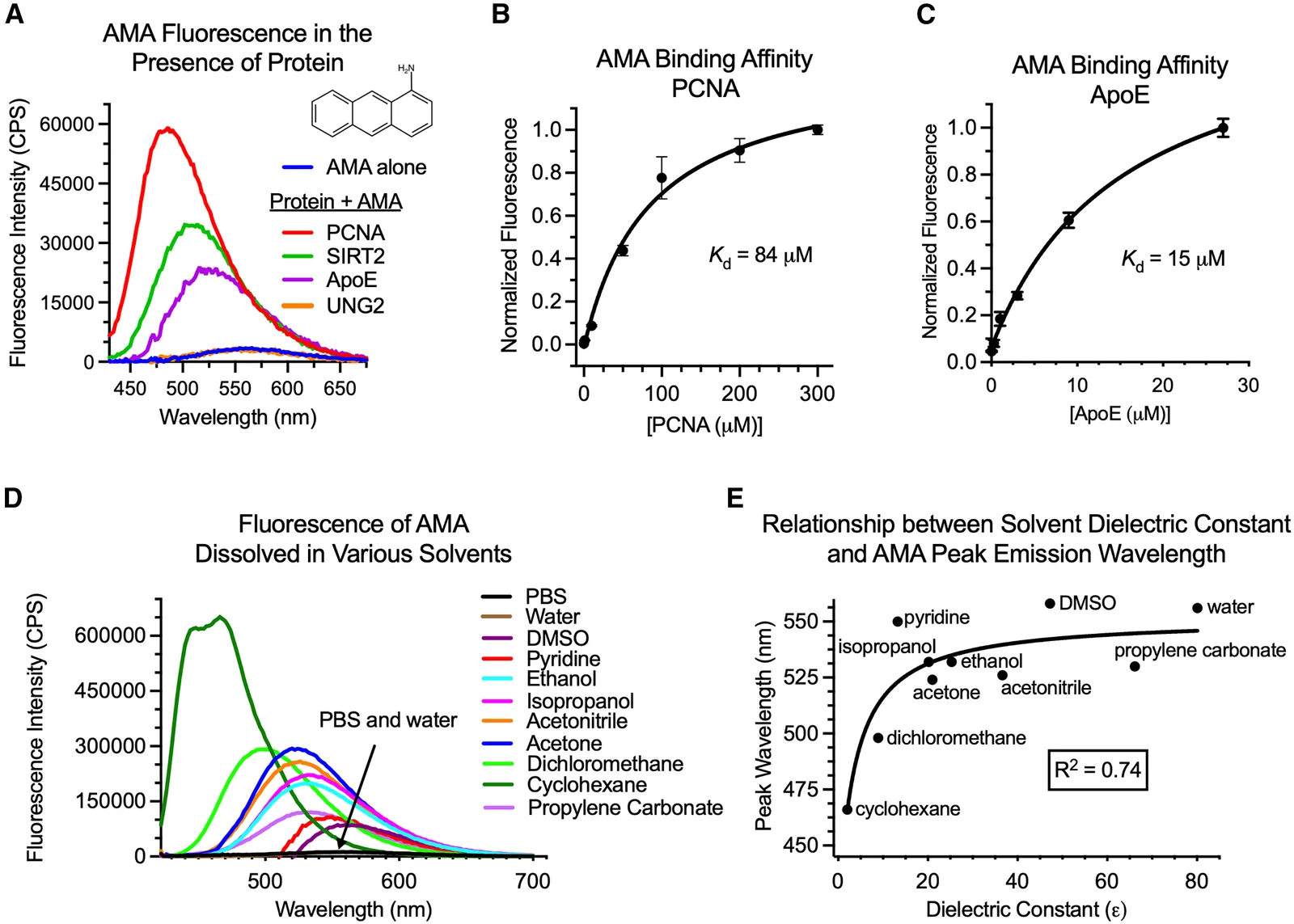
Fluorescence shifts of AMA when bound to proteins and in various solvents. (A) Fluorescence emission spectra of 100 nM AMA in PBS by itself or with 10 μM of the indicated proteins. (B) Binding isotherm showing the interaction of AMA with recombinant PCNA. Data points represent mean ± standard error. (C) Binding isotherm showing the interaction of AMA with recombinant ApoE. Data points represent mean ± standard error. (D) Fluorescence emission spectra of 200 nM AMA dissolved in the indicated solvents. (E) Nonlinear relationship between solvent ε and the peak emission wavelength of AMA from the data shown in (D). ε values were published elsewhere.^68^

To explore biophysical, chemical, and pharmacologic features of hydrophobic protein sites, we aimed to identify new interaction partners of AMA and to characterize its binding sites by measuring how the ligand’s fluorescence was affected by various proteins and chemical environments. In PBS buffer alone (pH 7.4), 100 nM AMA exhibited a peak emission wavelength of 560 nm with very weak fluorescence intensity (Figure 2A). The amine of AMA is neutral in PBS with a large dipole in the excited state, which promotes hydrogen bond interactions with the aqueous solvent and dielectric stabilization of the excited state, which contribute to nonradiative decay (i.e., loss of energy without light emission).^21^^,^^24^^,^^69^^,^^70^^,^^71^ However, in the presence of certain proteins including 10 μM PCNA, ApoE, or SIRT2, the fluorescence intensity of AMA increased up to 17-fold, and its peak emission wavelength significantly blueshifted, which was indicative of AMA binding to hydrophobic regions on the proteins (Figures 2A; Table 1).^22^^,^^23^ In the protein environment, the fluorophore was shielded from aqueous PBS, reducing polar solvent interactions that dampen AMA fluorescence.^21^^,^^24^ In contrast, the fluorescence intensity of AMA was unchanged in the presence of other proteins (e.g., UNG2), which indicated a lack of AMA interaction with hydrophobic sites on those macromolecules (Figures 2A; Table 1). The ability of proteins to induce changes in AMA fluorescence was not related to protein size, indicating that there was some binding selectivity for AMA beyond bulk nonspecific interactions. We further determined a *K*_d_ of 84 μM for AMA’s interaction with PCNA and a *K*_d_ of 15 μM for its interaction with ApoE (Figures 2B and 2C). We previously characterized the molecular interaction between AMA and SIRT2, which occurred with a *K*_d_ value of 37 μM.^27^

**Table 1:** Fluorescence changes of 100 nM AMA in the presence of 10 μM protein

Protein	Molecular weight (kDa)^a^	Fold change in AMA fluorescence intensity	AMA peak emission wavelength (nm)	Site dielectric constant (ε)^b^
Proliferating cell nuclear antigen (PCNA) (*H*. *sapiens*)	29	17	486	4
Sirtuin isoform 2 (SIRT2) (*H*. *sapiens*)	37	10	506	7
Apolipoprotein E (ApoE) (*H*. *sapiens*)	34	7	526	15
Pyruvate kinase (*O*. *cuniculus*)	57	3	530	19
ΔN555-Replication factor C (RFC) (*H*. *sapiens*)	217	3	532	21
Sirtuin isoform 3 (*H*. *sapiens*)	31	2	532	N/D^c^
Lactate dehydrogenase (*O*. *cuniculus*)	37	2	538	N/D^c^
Single-stranded binding protein (*E*. *coli*)	19	2	560	N/D^c^
Thermolysin (*G*. *stearothermophilus*)	36	1	580	N/D^c^
Replication protein A-70N (*H*. *sapiens*)	13	1	560	N/D^c^
Uracil DNA glycosylase isoform 2 (UNG2) (*H*. *sapiens*)	35	1	550	N/D^c^
Ubiquitin-like protease 1 (*S*. *cerevisiae*)	25	1	552	N/D^c^
FAM72a (*H*. *sapiens*)	17	1	532	N/D^c^
Streptavidin (*S*. *avidinii*)	17	1	532	N/D^c^

a Molecular mass of the protein polypeptide that was expressed and/or purified. Purification tags such as poly-histidine were excluded from the calculation.

b Determined by interpolation from the standard curve in Figure 2E.

c N/D, not determined. The signal above background was weak and noisy.

We were intrigued by the different emission wavelengths that AMA exhibited when bound to different proteins considering the experimental conditions were otherwise the same. We reasoned that variations in the dielectric microenvironment of the binding sites affected AMA’s emission. To demonstrate this, we measured the fluorescence of AMA dissolved in solvents covering a wide range of polarities in the absence of protein (Figure 2D). We observed a strong nonlinear correlation between the dielectric constant (ε) of the solvent and the peak emission wavelength of AMA (Figure 2E). The hyperbolic relationship has been seen for other solvatochromic fluorophores and reflects a nonlinear dependence of excited-state stabilization on solvent dielectric constant^72^^,^^73^; polarity effects increase steeply at low ε but tend to saturate at high ε due to limits in solvent reorientation around the excited fluorophore and specific solute-solvent interactions. The spectroscopic absorbance of AMA did not change as dramatically in the various solvents (Figure S1).

We determined ε values for the AMA binding sites on our proteins of interest by interpolation using the data in Figure 2E as a standard curve. The AMA site on PCNA had an ε of 4, which was very apolar and indicated a chemical environment within the protein that was similar to cyclohexane (Table 1). The AMA binding sites on SIRT2 and ApoE were more polar and had dielectric constants that were in line with dichloromethane or pyridine (Figures 2E; Table 1). As an additional point of reference for hydrophobicity, and to broaden the utility of AMA as a dielectric constant probe, we generated phospholipid bilayers in PBS using DOPC and measured the fluorescence of AMA after it partitioned into the membrane.^22^ AMA reported a peak emission wavelength of 536 nm and an ε value of 27 at its depth inside the membrane (Figure S2). In conclusion, we identified several new protein binding partners of AMA and found that the ligand reported a broad range of dielectric environments inside protein hydrophobic sites.

### Discovery of a highly apolar ligand site on PCNA

**Figure 3:**
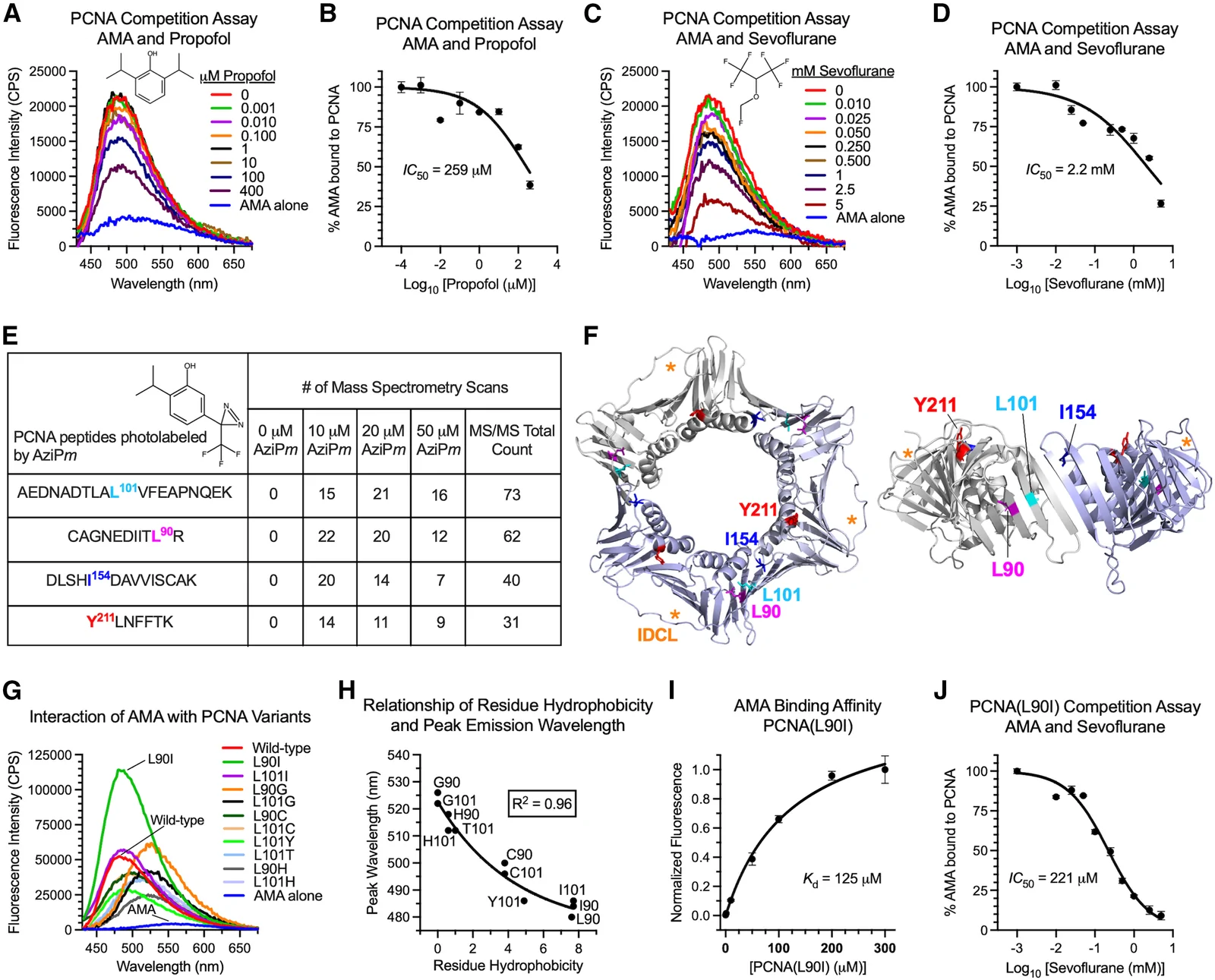
Interaction of AMA and anesthetics at a hydrophobic site near L90 and L101. (A) Emission spectra of 100 nM AMA equilibrated with 10 μM PCNA and the indicated propofol concentrations. Propofol caused a dose-dependent decrease in AMA fluorescence, which indicated AMA unbinding PCNA and returning to the aqueous solution. (B) Displacement of AMA from PCNA by propofol, as determined from the data in (A). The peak fluorescence intensity of AMA at each propofol concentration was normalized to the intensity of AMA bound to PCNA in the absence of competitor. Data points represent mean ± standard error. (C) Emission spectra of 100 nM AMA equilibrated with 10 μM PCNA and the indicated sevoflurane concentrations. (D) Displacement of AMA from PCNA by sevoflurane, as determined from the data in (C). (E) Table of peptides that were photolabeled during experiments with different concentrations of AziP*m*. Spectra can be found in Figure S4. (F) Left: location of AziP*m*-photolabeled residues on a PCNA trimer with the interdomain connecting loops (IDCL) also indicated as orange asterisks. Right: a side view of the structure (only two subunits are shown for clarity). (G) Emission spectra of 100 nM AMA equilibrated with 10 μM of the indicated PCNA variants. (H) Relationship between the peak emission wavelength of AMA when bound to the PCNA variants in (G) and the hydrophobicity of the residue substituted at position 90 or 101. Residue hydrophobicity scores were reported elsewhere.^44^ (I) Binding isotherm showing the interaction of AMA with recombinant PCNA(L90I). (J) Displacement of AMA from PCNA(L90I) by sevoflurane.

We began our search for AMA binding sites on several proteins that were not previously known to us as binding partners for AMA or chemically similar molecules including general anesthetics. We were unable to competitively displace AMA from its binding site on ApoE using propofol or sevoflurane (Figure S3). ApoE binds various lipids and fatty acyl chains that were incompatible with AMA competition assays because of their tendency to form bilayers or micelles that attract the fluorophore (Figure S2).^74^ We also found that sevoflurane did not displace AMA from pyruvate kinase; the inability of sevoflurane to displace AMA from ApoE or pyruvate kinase may indicate nonsaturable or nonconserved ligand interactions. In contrast, AMA was competitively displaced from PCNA by propofol and sevoflurane (Figures 3A–3D), with displacement from a shared binding site reducing AMA’s fluorescence as it returned to the aqueous environment. Propofol displaced AMA from PCNA with an *IC*_50_ of 259 μM, and sevoflurane displaced AMA with an *IC*_50_ of 2.2 mM (Figures 3B and 3D). These results suggested a specific binding site on PCNA that could be saturated with ligand. Coupled with the compelling hydrophobicity of the ligand site (Table 1) and the central role of PCNA in cell physiology, we selected it for further study.

To gain insight into the location of the AMA binding site on PCNA, we performed photoaffinity labeling using AziP*m*, a photoactivatable analog of propofol with similar physicochemical properties (Figure 1).^36^ The diazirine of AziP*m* undergoes photolysis upon exposure to long-wave UV to yield a reactive carbene that covalently attaches the ligand to its protein binding sites, which reliably overlap with sites for propofol and AMA.^27^^,^^36^^,^^49^^,^^75^ After photolabeling PCNA with AziP*m*, LC-MS/MS was used to identify four amino acids on PCNA that interacted with the anesthetic ligand (Figure 3E). This was evident because the photolabeled residues contained 216-Da mass shifts from an AziP*m* adduct (Figure S4). PCNA residues L90 and L101 were the most heavily labeled by AziP*m* (Figure 3E). These residues sit adjacent to each other in the folded protein within the same PCNA domain, and the leucine side chains were buried deep in the protein’s hydrophobic core (Figure 3F). Photolabeled residues I154 and Y211 were more exposed to the solvent, with Y211 also serving as a phosphorylation site (Figure 3F).^76^

We hypothesized that mutating amino acids located within the AMA binding site would alter the dielectric environment of the site, thereby changing AMA’s emission peak to report on the polarity of the site. We started by mutating residues L90 and L101 on recombinant PCNA proteins because of their strong interaction with the propofol photolabel. We found that the hydrophobicity of the amino acid substituted at position L90 or L101 determined the degree of AMA’s blueshift, which supported the idea that the AMA binding site was being manipulated (Figures 3G and 3H). PCNA tolerated the incorporation of slightly less apolar residues at L90 and L101, but incorporation of charged or polar residues produced misfolded PCNA aggregates, as observed during size exclusion chromatography (e.g., lysine, serine, or glutamine mutations). Alanine substitutions at I154 and Y211 also resulted in misfolded protein; we cannot rule out that these photolabeled residues contributed to secondary sites for the small molecules, but we did not pursue them further. We also mutated residues in surface pockets on PCNA that yielded properly folded protein with the AMA binding signal unaffected (Figure S5), although hydrophilic AMA sites may not produce measurable changes in fluorescence. Nonetheless, this mutational analysis was consistent with the photolabeling results and indicated that AMA and the anesthetics interacted with L90 and L101 on PCNA.

Finally, we noted a peculiar PCNA variant from the above assays with an isomeric substitution in the putative AMA site (L90I) where the hydrophobicity of the side chain was marginally altered. The peak emission wavelength of AMA did not change when bound to PCNA(L90I), yet the fluorescence intensity was significantly elevated (Figure 3G). We determined a *K*_d_ of 125 μM for the interaction of AMA with PCNA(L90I), which was similar to wild-type PCNA (*K*_d_ = 84 μM) (Figure 3I). We displaced AMA from PCNA(L90I) using sevoflurane and determined an *IC*_50_ of 221 μM. Sevoflurane had 10-fold higher affinity for PCNA(L90I) compared with wild-type PCNA where it displaced AMA with an *IC*_50_ of 2.2 mM (Figure 3D). These changes in binding that resulted from subtle mutation of L90 confirmed this as the location of a highly apolar ligand site on PCNA.

### Structure and dynamics of the cryptic PCNA binding site and ligand interactions

**Figure 4:**
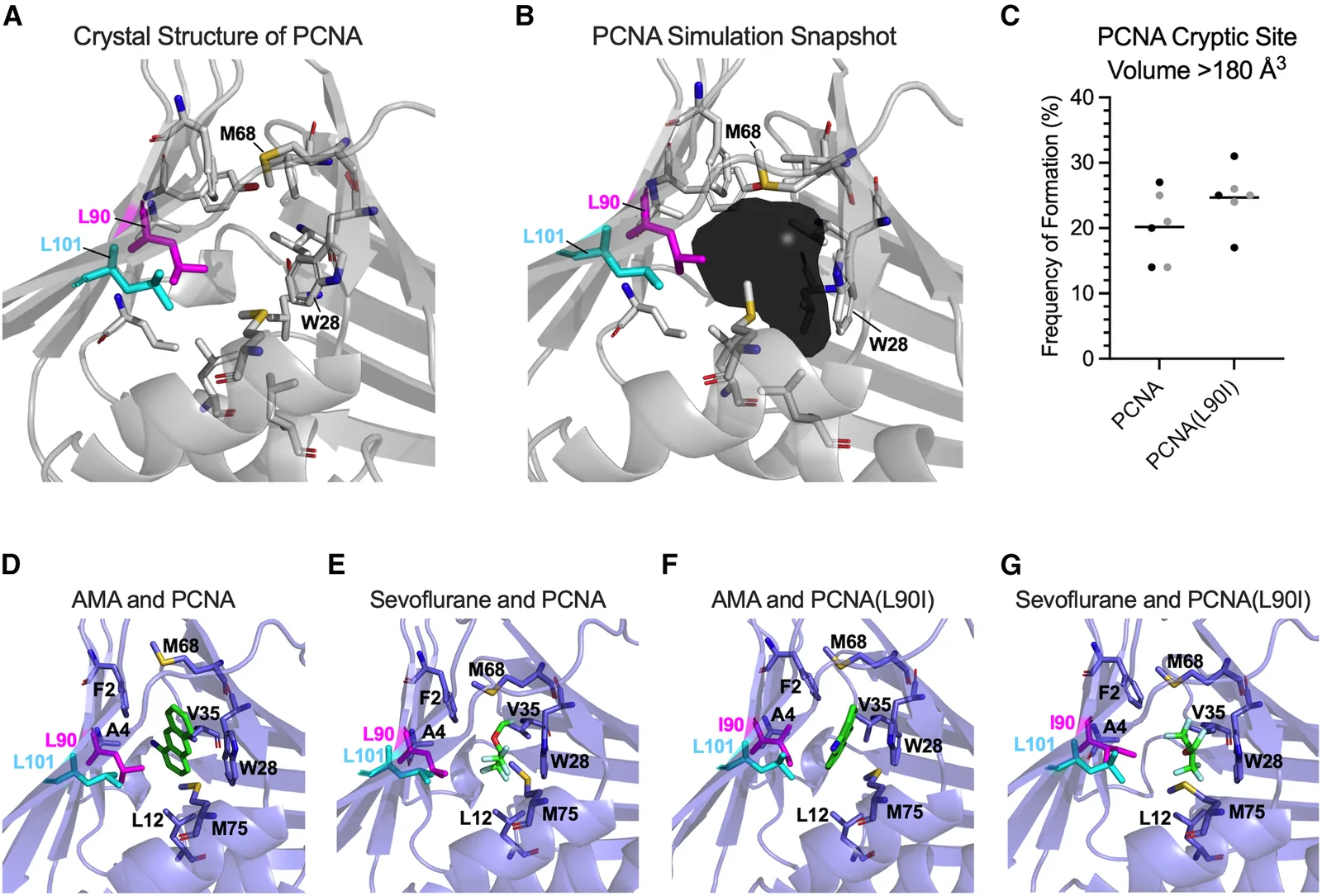
A cryptic binding site for small molecules in the hydrophobic core of PCNA. (A) View of residues near L90 and L101 in the crystal structure of apo-PCNA (PDB: 1VYM). (B) View of residues near L90 and L101 from a simulation snapshot of PCNA with the Fpocket-identified internal cavity shown in black. (C) Percent of MD simulation that each pocket formed near residues L90 and L101 that was large enough to accommodate general anesthetic ligands (black dots, three subunits from 1-μs simulation; gray dots, three subunits from 0.5-μs simulation; black bar, mean). The analysis was performed with MDpocket. (D) Highest scoring pose of AMA docked to PCNA. (E) Highest scoring pose of sevoflurane docked to PCNA. (F) Highest scoring pose of AMA docked to PCNA(L90I). The plane of the ligand is perpendicular to the plane of panel. (G) Highest scoring pose of sevoflurane docked to PCNA(L90I).

There are dozens of high-resolution PCNA structures where the homotrimer is bound to various polypeptides or ligands at a conserved surface pocket called the interdomain connecting loop (IDCL), through which PCNA orchestrates DNA metabolism.^31^ The IDCL site was rather distant (>30 Å) from the anesthetic site at L90 and L101 (Figure 3F). Initially, we analyzed the crystal structure of apo-PCNA to mimic the conditions of our ligand binding assays (PDB: 1VYM),^51^ and Fpocket identified >20 solvent-exposed surface cavities that were theoretically large enough to accommodate AMA, which has a molecular volume of 179 Å^3^ (Figure S6). However, no binding pocket was identified near residues L90 and L101, which were packed with other hydrophobic side chains in the core of the protein (Figure 4A). We considered how protein movement can transiently open and close small cryptic sites that otherwise may not appear in static structures.^5^^,^^6^^,^^9^^,^^77^^,^^78^ We ran two atomistic MD simulations of PCNA in water (1.5 μs total, Figure S7), and indeed, a space that was large enough to accommodate our ligands transiently appeared near L90 and L101 (Figure 4B). In part, this pocket was formed by the rotation of side chains out of the hydrophobic space (e.g., W28 and M68) (Figures 4A and 4B).

We examined the structure and dynamics of the cryptic anesthetic binding site that transiently appeared inside the hydrophobic core of PCNA and included residues L90 and L101 (Figure 4B). The site formed and disappeared in each subunit of the homotrimer independently and without correlation to other subunits (Figure S8). On average, a subunit contained a pocket that was large enough to bind anesthetics (>180 Å^3^) for 20% of the simulations (Figure 4C). The site was lined with side chains from hydrophobic residues that packed into the protein core (Table S2), and we could detect no ions or water molecules entering the site. We also simulated PCNA(L90I) for 1.5 μs. The physicochemical features of the hydrophobic site were similar on PCNA(L90I), except that the anesthetic pocket was open more often than wild-type protein (25% of the simulation) and had a larger average volume (median pocket volume = 120 Å^3^ for wild-type and 137 Å^3^ for PCNA(L90I) (Figure 4C; Figure S8)). The L90 side chain frequently contacted more distant residues than the I90 side chain, which positions a terminal methyl group one carbon closer to the protein backbone (Figure S8).

To evaluate ligand binding to PCNA’s cryptic site, we extracted structural snapshots from the simulations where the site was open in one or more subunits, and then, we docked AMA or sevoflurane onto wild-type PCNA or PCNA(L90I). AMA and sevoflurane could typically be docked into the internal pocket if its volume was sufficiently large with calculated binding free energies of ∼6 kcal/mol (Figures 4D–4G). The predicted poses for AMA and sevoflurane were heterogeneous when docked to the PCNA proteins, and the shape of the pocket was also heterogeneous. Ligand-protein binding was mediated by hydrophobic or van der Waals interactions. We then simulated PCNA and PCNA(L90I) with each of the proteins bound to three molecules of sevoflurane (one molecule per subunit). Sevoflurane remained bound to all of the cryptic sites during 1-μs simulations, although the molecules frequently reoriented within the site (Figure S9 and Video S1). Two additional simulations of PCNA or PCNA(L90I) bound to a single AMA molecule produced similar behavior in which AMA remained in the site with occasional reorientation (Figure S9 and Video S2). Furthermore, another simulation with AMA initially docked to a different PCNA(L90I) starting structure showed AMA unbinding after ∼310 ns and returning to the aqueous solvent, thereby providing a plausible entry/exit route to the protein’s core (Figure S9).

Video S1. MD simulation of sevoflurane bound to a cryptic pocket on PCNA (10 ns/frame) Even though sevoflurane remained bound to the internal pocket, note the mobility and different poses of the anesthetic, which was colored by atom (carbon, cyan; fluorine, brown; oxygen, red; hydrogen, white). RMSD for this ligand can be found in Figure S9.

Video S2. MD simulation of AMA bound to a cryptic pocket on PCNA (10 ns/frame) The molecule remained bound in the pocket but sampled different poses, including a complete reorientation of its amine near the end of the simulation. AMA was colored by atom (carbon, cyan; nitrogen, blue; hydrogen, white). RMSD for this ligand can be found in Figure S9.

### Biophysical effects of ligand binding to the cryptic site

**Figure 5:**
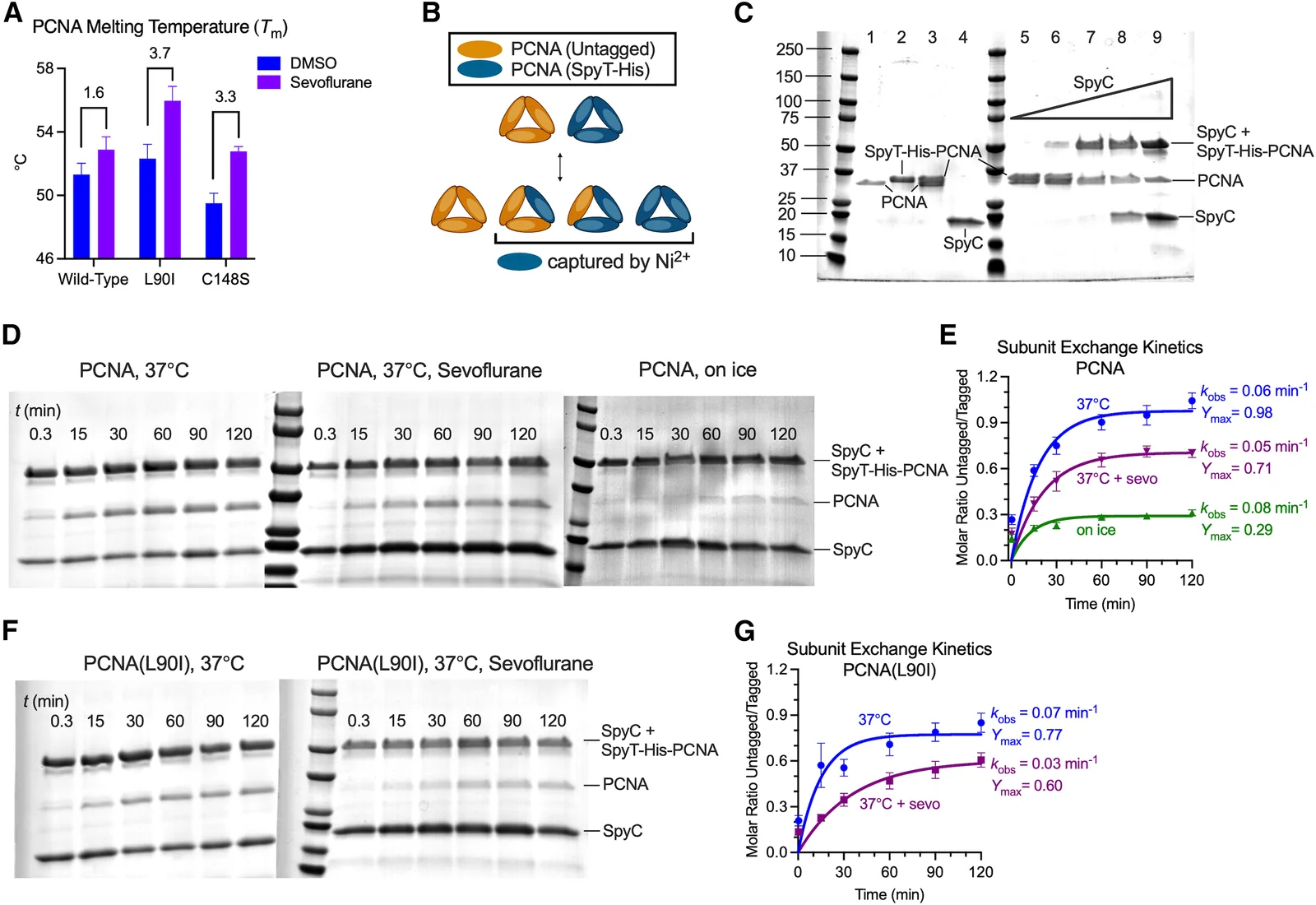
Cryptic site occupancy stabilizes the structure of the PCNA trimer. (A) Melting temperatures of recombinant PCNA proteins increased in the presence of 2.5 mM sevoflurane. (B) Scheme for subunit exchange assays where untagged PCNA exchanges subunits with a recombinant PCNA variant containing a SpyTag and 6xHis tag (SpyT-His), which can be captured on Ni^2+^ resin with accompanying subunits. (C) Coomassie-stained SDS-PAGE gel showing components of PCNA subunit exchange assays and the reaction of SpyT-His-PCNA with SpyCatcher. Lane 1, 5 μM (untagged) PCNA; lane 2, 5 μM SpyT-His-PCNA; lane 3, 5 μM PCNA +5 μM SpyT-His-PCNA; lane 4, 20 μM SpyCatcher; lanes 5–9 all contained 5 μM PCNA +5 μM SpyT-His-PCNA along with the following SpyCatcher concentrations: lane 5, 0 μM; lane 6, 1 μM; lane 7, 5 μM; lane 8, 10 μM; lane 9, 20 μM. (D) Representative Coomassie-stained gels from subunit exchange assays using PCNA at different temperatures with and without 2.5 mM sevoflurane. Over time, increasing amounts of untagged PCNA associated with SpyT-His-PCNA, which were captured together on Ni^2+^ resin before being eluted. The eluate was then reacted with SpyCatcher and run on the gel. (E) Kinetics of PCNA subunit exchange for the assay conditions shown in (D). Every data point represents mean ± standard error from three to five independent assays. (F) Coomassie-stained gels from subunit exchange assays using PCNA(L90I) with and without 2.5 mM sevoflurane. (G) Kinetics of PCNA subunit exchange for the assay conditions shown in (F). Every data point represents mean ± standard error from three to five independent assays.

We considered how ligand binding to the cryptic site could affect PCNA stability, dynamics, and activity. We focused our studies on sevoflurane because we could achieve high occupancy of PCNA sites due to its higher solubility than AMA, which was limited to only ∼33 μM.^22^ We equilibrated wild-type PCNA or PCNA(L90I) with 2.5 mM sevoflurane to assess how the anesthetic affected the thermostability of the protein. This ligand concentration was sufficient to occupy ∼53% of available cryptic sites in the wild-type protein and ∼92% in the mutant based on their different affinities for sevoflurane, and thermostability was assessed by monitoring the change in PCNA’s intrinsic fluorescence upon denaturation. The melting temperature (*T*_m_) of wild-type PCNA increased 1.6°C in the presence of sevoflurane, and the *T*_m_ of PCNA(L90I) increased by 3.7°C when bound to the ligand (Figure 5A; Figure S10). As a negative control, we found that sevoflurane reduced the *T*_m_ of UNG2, which lacks a lipophilic binding site for AMA (Figures 2A; Table 1), by ∼1°C, indicating weak destabilization of its folded structure (Figure S10).

Interestingly, a naturally occurring genetic variation in human PCNA yields the protein PCNA(C148S), which was reported to be a thermolabile homotrimer and the cause of human disorders that are characterized by impaired DNA replication and repair.^66^ The destabilizing C148S mutation and the cryptic site are sterically separated and distant at ∼21 Å apart; nonetheless, we hypothesized that ligand interactions in the cryptic site could reverse the thermosensitive phenotype of PCNA(C148S). We confirmed that the *T*_m_ of PCNA(C148S) was 1.8°C lower than wild-type PCNA (Figure 5A; Figure S10). PCNA(C148S) interacted with AMA and sevoflurane with a similar affinity as wild-type PCNA (Figure S10), and the dielectric constant of its cryptic binding site was unaltered by the mutation (ε = 4). Sevoflurane binding increased the *T*_m_ of PCNA(C148S) by 3.3°C, almost matching the *T*_m_ of wild-type PCNA bound to the ligand (Figure 5A; Figure S10). This demonstrated that ligand interaction in the cryptic site can overcome a long-range destabilizing protein mutation, although it is unclear whether stabilization and destabilization arise through the same mechanistic pathway.

Although PCNA functions as a homotrimer, its subunits can dissociate and reassociate in solution with a *K*_d_ value of ∼21 nM.^79^^,^^80^ Because sevoflurane stabilized the protein against unfolding, we thought that the ligand might enhance the association of its individual subunits within the homotrimer. We developed a strategy to measure subunit exchange between different PCNA trimers. Briefly, untagged recombinant PCNA was mixed with a tagged PCNA variant that contained a 6xHis tag and SpyTag on its N terminus (“SpyT-His-PCNA”) (Figure 5B).^81^ Over time, untagged PCNA subunits exchanged into assemblies with SpyT-His-PCNA (Figure 5B). We quantified the level of untagged PCNA that pulled down with SpyT-His-PCNA on Ni^2+^ resin; before analysis by SDS-PAGE, we covalently reacted the SpyTag with SpyCatcher to improve the resolution of the protein bands on the gel (Figure 5C).^81^ If untagged PCNA and SpyT-His-PCNA mixed and distributed evenly to an equilibrium during the course of the assay, then the molar ratio of untagged/tagged PCNA in the final pull-down would be 3/4 or 0.75 (see materials and methods).

For wild-type PCNA, the rate of subunit exchange followed first-order kinetics (*k*_obs_ = 0.05 min^−1^) and was within range of a rate determined for the exchange of yeast PCNA subunits under slightly different conditions.^34^ It is probable that single subunit exchanges dominated in the assay.^34^^,^^79^ For assays conducted at 37°C, the molar ratio of untagged/tagged PCNA in the final pull-down plateaued at 0.86 after ∼90 min (Figures 5D and 5E, blue *Y*_max_ value), which was close to the ideal value of 0.75, indicating complete mixing of the PCNA subunits. We determined that sevoflurane reduced the level of subunit exchange that occurred between untagged PCNA and SpyT-His-PCNA (Figures 5D and 5E, purple *Y*_*max*_ value). This implied that the pool of PCNA that was exchanging subunits was reduced in the presence of sevoflurane. As a positive control, we conducted experiments on ice where the homotrimer was very stable and the pool of exchanging subunits declined further (Figures 5D and 5E, green *Y*_max_ value), which was consistent with a previous report.^79^ The PCNA(L90I) variant behaved similarly to wild-type PCNA at 37°C (Figures 5F and 5G). Sevoflurane reduced the rate and level of subunit exchange that occurred in the mutant to a greater degree than wild-type PCNA, likely due to greater occupancy of the cryptic sites (Figures 5F and 5G).

Finally, we confirmed that the cryptic binding site was functionally independent from the IDCL site where PCNA binds to other proteins. We used fluorescence anisotropy-based binding assays to determine that sevoflurane and propofol did not affect the interaction of PCNA with full-length UNG2 (Figure S11).^40^ We found that propofol did not affect PCNA binding to a peptide called Pogo-Ligase that binds to the IDCL site (Figure S11).^40^^,^^82^^,^^83^ We found that sevoflurane did not affect the interaction of PCNA with a p15 peptide that binds to the same IDCL site, but with extended interactions (Figure S11).^84^ Last, we examined whether sevoflurane binding interfered with the loading of PCNA onto DNA by RFC, which is driven by ATP hydrolysis and is also mediated through IDCL site interactions.^85^^,^^86^ Sevoflurane did not affect the activity of RFC or its ability to load PCNA onto DNA (Figure S12).

### Conservation of internal hydrophobic sites in diverse sliding clamps

**Figure 6:**
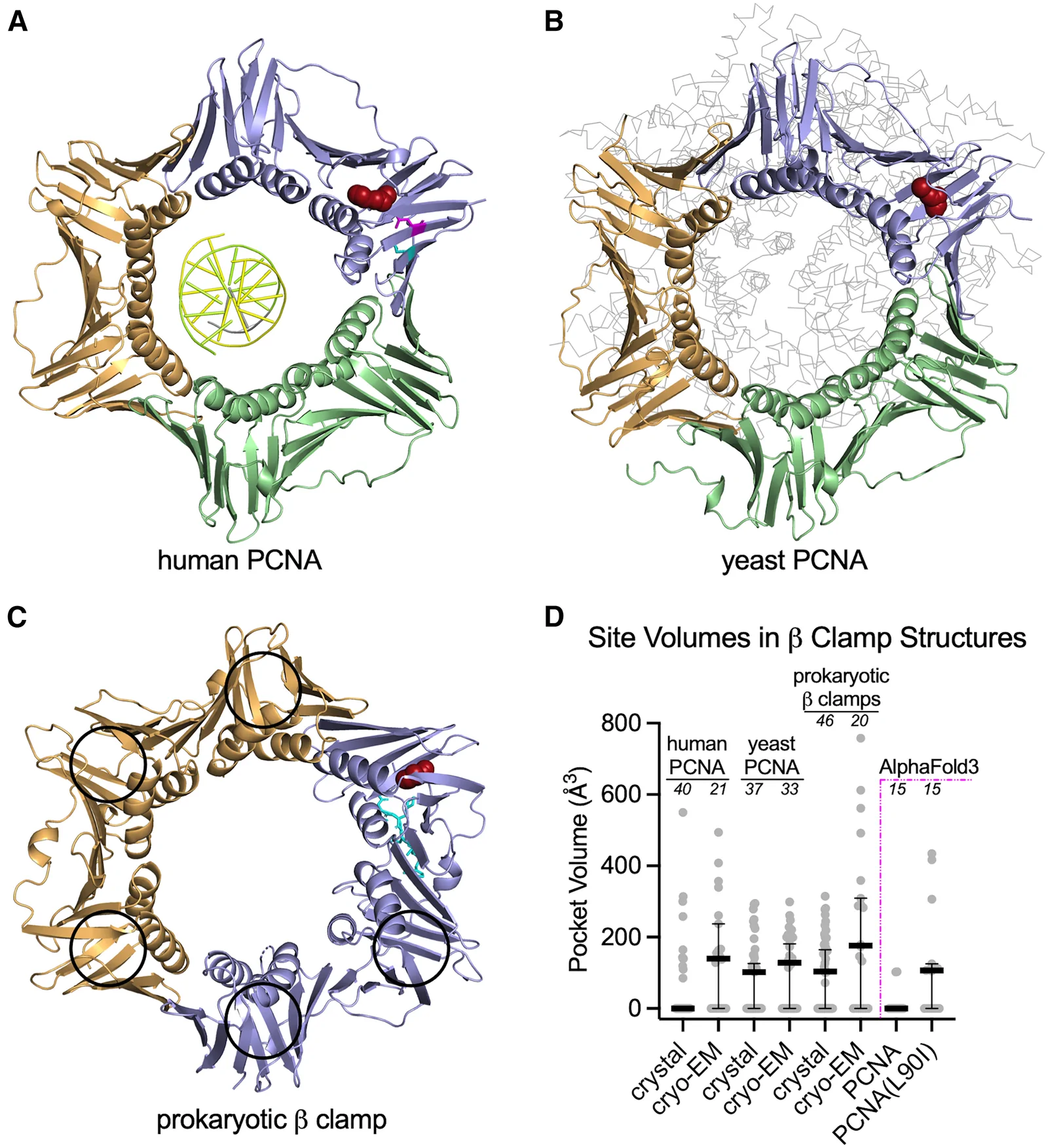
The hydrophobic site is conserved across the PCNA/β clamp fold. (A–C) Representative structures where we identified internal hydrophobic sites with variable volumes at analogous locations. Identified pockets are colored deep red spheres in the images. (A) X-ray crystal structure of human PCNA bound to DNA with L90 and L101 colored magenta and cyan, respectively (PDB: 6GIS). (B) X-ray crystal structure of yeast PCNA bound to replication factor C, which is shown as gray ribbons bound to the other side of the protein (PDB: 1SXJ). (C) X-ray crystal structure of the dimeric sliding clamp from the prokaryote *Pseudomonas aeruginosa* bound to a synthetic peptide molecule, colored cyan (PDB: 4TSZ). In panel (C), we also circled the five other pseudodomain locations in the β clamp fold where internal pockets may exist on select proteins (Table S3). (D) Internal hydrophobic site volumes for every subunit in 85 experimental PCNA or β clamp structures, along with data for five human PCNA or PCNA(L90I) AlphaFold3 models. Every gray point represents a single subunit, and the total number of analyzed subunits is written in italics above each data column. The black bar represents the median with the 95% confidence interval as error bars.

We analyzed ∼100 homologous PCNA and β clamp structures from humans, yeast, and prokaryotic organisms to test the hypothesis that the hydrophobic pocket is a conserved structural feature of the proteins, along with its variable volume. Many of these PCNA/β clamp proteins were bound to proteins, ligands, or DNA or may have contained point mutations, but nonetheless, they shared a homologous fold. Indeed, the hydrophobic pocket could be observed within the cores of various experimental protein structures from both X-ray crystallography and cryoelectron microscopy (Figures 6A–6D; Table S3). Some β clamp proteins, including human PCNA, occasionally featured internal pockets in adjacent pseudodomains (Figure 6C; Table S3). Even AlphaFold models of PCNA and PCNA(L90I) exhibited variable pocket volumes within subunits of the same homotrimer, and in the case of wild-type PCNA, the AlphaFold-predicted pockets were too small in size for the ligands examined in this study (Figure 6D). This analysis supports the idea that a localized hydrophobic space, dynamic in volume, is a conserved feature of this protein family. Additionally, the presence or detectability of the site may be dependent on many factors. As an example, for the human experimental structures, we found a significant relationship where lower resolution structures tended to provide larger pocket volumes (Figure S13). How the sites were affected by other factors such as DNA or protein interactions (including crystal contacts), rotameric conformations in the final model,^87^^,^^88^ and user-defined algorithm thresholds (e.g., pocket detection cutoffs) remains to be systematically evaluated.

## Discussion

Using AMA as a small fluorescent probe, we identified a cryptic site in the core of human PCNA that can accommodate general anesthetics and other ligands with similar chemical features. The site forms because of packing defects among hydrophobic side chains and because of their spontaneous movement within the pocket. More than a dozen residues contributed to the formation of the site, which continually changed its shape and size during MD simulations, yet the site was sensitive to subtle perturbations like point mutations. We found that the site existed transiently as empty space in the protein core that weakened the strength of the PCNA oligomer; ligand binding to the site stabilized the PCNA trimer and protected the protein from thermal denaturation. Interestingly, after conducting DNA replication and repair activities at replication forks, some fraction of trimeric PCNA spontaneously dissociates to unload itself from dsDNA,^34^^,^^35^ which might be tuned or influenced through this binding site. Finally, homologous β clamp proteins from yeast and prokaryotes have a similar pocket that forms in their hydrophobic core, indicating that this feature is conserved across the fold.

Related to cryptic site mutagenesis, the isomeric variant PCNA(L90I) had physical changes in pocket architecture and pharmacology compared with wild-type PCNA. Using BLAST and other tools,^89^ we were unable to find a naturally occurring vertebrate PCNA with an isoleucine at that position, suggesting a selective pressure to retain the leucine. Notably, the emergence of isoleucine at that position in several invertebrates was accompanied by additional substitutions in neighboring pocket residues (e.g., *Daphnia pulex* or *Littorina saxatilis*). Together, these observations support the idea that the cryptic site of PCNA evolved to tune the activity of the DNA binding protein, rather than being a passive by-product of protein folding. A more refined framework is needed for understanding the evolutionary conservation and divergence of site features with regard to protein sequence, structure, and function. Our biochemical assays showed limited crosstalk between the cryptic site and the highly conserved IDCL site. However, examining the context dependence of internal pockets in proteins from various organisms must be approached cautiously with static experimental and *de novo* models that do not capture the full range of structural sampling observed in solution or simulations or that may bias certain states due to experimental parameters or training data sets.

Our fluorescence experiments measured the dielectric constant of AMA binding sites on proteins including its hydrophobic site in the core of PCNA (ε = 4), which was almost as apolar as cyclohexane (ε = 2). Dielectric constant is a parameter of polarizability, which is critical for calibrating computational models of electrostatics, solvation, and p*K*_a_ calculations.^11^ Because the dielectric environment governs how charges and dipoles interact within a protein, it directly influences folding stability, conformational transitions, and ligand recognition.^90^^,^^91^^,^^92^ Previously, an environmentally sensitive fluorescent amino acid, which was site specifically incorporated on the model protein GB1, reported an ε of 6 in a buried hydrophobic domain and a range of ε values at other sites, which were consistent with our measurements.^72^ The small size, structural plasticity, and hydrophobicity of our site reduced the possibility for high-affinity ligand interactions. Nonetheless, general anesthetics are evidence that these sites can be targeted for therapeutic value, and even weak binding ligands can offset destabilizing protein mutations by lowering the free energy of their folded state.^93^ The finding that sevoflurane had 10-fold higher affinity for PCNA(L90I) compared with wild-type PCNA, without an accompanying change in the dielectric environment of the site, also indicated that ε values alone were not sufficient to predict relative binding affinities for lipophilic ligands. Finally, the L90I substitution increased the fluorescence of AMA bound to the site compared with wild type. This could occur if the mutation increased the availability of the site, which was measured to a limited extent, but we also detected different interactions among residue side chains in the mutated cryptic site (Figure 4C; Figure S8). To some degree, ligand-protein interactions should change in the mutant cryptic site, supported by the change in sevoflurane affinity for PCNA(L90I), and changes in pocket geometry, ligand rigidity, or ligand orientation relative to nearby residues may affect AMA fluorescence intensity.

In conclusion, we used AMA as a fluorescence reporter of hydrophobic ligand sites to identify a cryptic binding site deep in the core of human PCNA. Ligands with similar physical and chemical features interact with PCNA at the site and alter its stability and dynamics. Although not explored here, it is possible that an endogenous ligand from the cell could bind to the site in humans or β clamp proteins from other organisms. As a general approach, we expect that screening or systematically probing proteins with fluorophores tuned to detect sites of specific sizes, shapes, or polarities could significantly expand the number of druggable sites in the proteome and uncover novel strategies to alter protein activities.

## Data and code availability

The experimental data sets underlying this study are openly available on Zenodo at https://zenodo.org/records/19630221 or https://doi.org/10.5281/zenodo.19630221.

## Acknowledgments

This work was supported in part by 10.13039/100000002National Institutes of Health grant R01GM135152 and 10.13039/100001774New Jersey Health Foundation grant PC 215-24 awarded to B.P.W. Additional support came from 10.13039/100000002National Institutes of Health grant R01GM135633 awarded to R.G.E. and a 10.13039/100005831Foundation for Anesthesia Education and Research grant awarded to E.R.W. The authors thank Drs. Benjamin Garcia and Natarajan Bhanu for assistance with mass spectrometry experiments. The authors acknowledge the use of BioRender to prepare the online graphical abstract.

## Author contributions

A.N.D. designed research, performed research, analyzed data, and wrote the manuscript; S.N.G. performed research; J.Y. performed research; D.E.J. performed research; J.F. performed research; E.R.W. performed research; R.G.E. contributed research tools; Z.L. contributed research tools and provided supervision; B.P.W. designed research, analyzed data, provided supervision, and wrote the manuscript. All authors reviewed the final manuscript.

## Declaration of interests

The authors declare no competing interests.

## Supporting citations

Refs. 94,95,96,97,98,99,100,101,102,103,104,105,106,107,108,109,110,111 appear only in the supplemental information.

## Supplemental information

Document S1. Figures S1–S13 and Tables S1–S3

Document S2. Article plus supplemental information
