# Supplementary material for: Biophysical insights into a cryptic ligand site in the hydrophobic core of human PCNA
Source: Biophys J. 2026 May 20;125(13):3471–88. doi: 10.1016/j.bpj.2026.05.027 (PMC13283471; doi:10.1016/j.bpj.2026.05.027)
Supplement: Supplementary file 1 [file mmc1.pdf]

Biophysical Journal, Volume *125*

Supplemental information

Biophysical insights into a cryptic ligand site in the hydrophobic core of human PCNA

Alexis N. Dispensa, Sharon N. Greenwood, Jason Yang, Daniel E. Logatto, James Fusco, E. Railey White, Roderic G. Eckenhoff, Zhiwei Liu, and Brian P. Weiser

Table S1. Previously reported proteins that interact with 1-aminoanthracene (AMA). ND = not determined.

Protein	K_d for AMA binding	AMA Binding Site Information	References
Serum albumin (*B. taurus*)	18 µM	ND	(1)
α-Acid glycoprotein (*H. sapiens*)	6 µM	ND	(2)
Apo-ferritin (*E. caballus*)	70-96 µM	Binding site shared with general anesthetics and lipids	(3–5)
β-lactoglobulin (*B. taurus*)	3 µM	ND	(2)
$GABA_A$ receptors (*H. sapiens*)	ND	Binding site shared with etomidate (β3 homopentamer); AMA potentiates GABA response (α1β2γ2L)	(3, 6)
Gastric triacylglycerol lipase (*C. familiaris*; *H. sapiens*)	8 µM	Binding site shared with β-octyl glucoside and is conformation-specific	(7, 8)
Leukocyte function-associated antigen-1 (*H. sapiens*)	ND	Binding site shared with lovastatin and general anesthetics	(9, 10)
Lipocalin 11 (*R. norvegicus*)	2 µM	ND	(11)
Odorant binding proteins (*S. scrofa*; *R. norvegicus*)	1-5 µM	Binding site shared with odorants, small lipophilic molecules, and general anesthetics	(2, 12–18)
Pheromone binding proteins (*M. brassicae*; *A. Polyphemus*)	1-5 µM	Binding site shared with pheromones, small lipophilic molecules, and fatty acids	(19, 20)
Salivary lipocalin (*S. scrofa*)	9-12 µM	Binding site shared with odorants and small molecules	(21)
Sirtuin isoform 2 (*H. sapiens*)	4-37 µM	Binding site shared with propofol and lipid-like molecules; affinity is conformation-dependent	(22)
Tear acid lipase-like protein (*M. auratus*)	12 µM	ND	(23)
Tubulin (*B. taurus*)	ND	AMA binds colchicine site and inhibits tubulin polymerization	(24)
Vomeronasal type 2 receptor (*M. musculus*)	ND	Binding site shared with pheromonal odorant 2-octanamine	(25)

Figure S1. UV-Vis absorbance spectra of AMA dissolved in various solvents. These spectra were collected using 1 cm pathlength cuvettes using a ThermoFisher nanodrop instrument in cuvette mode. The solvent alone was recorded as the background and subtracted from the 30 μM AMA measurement.

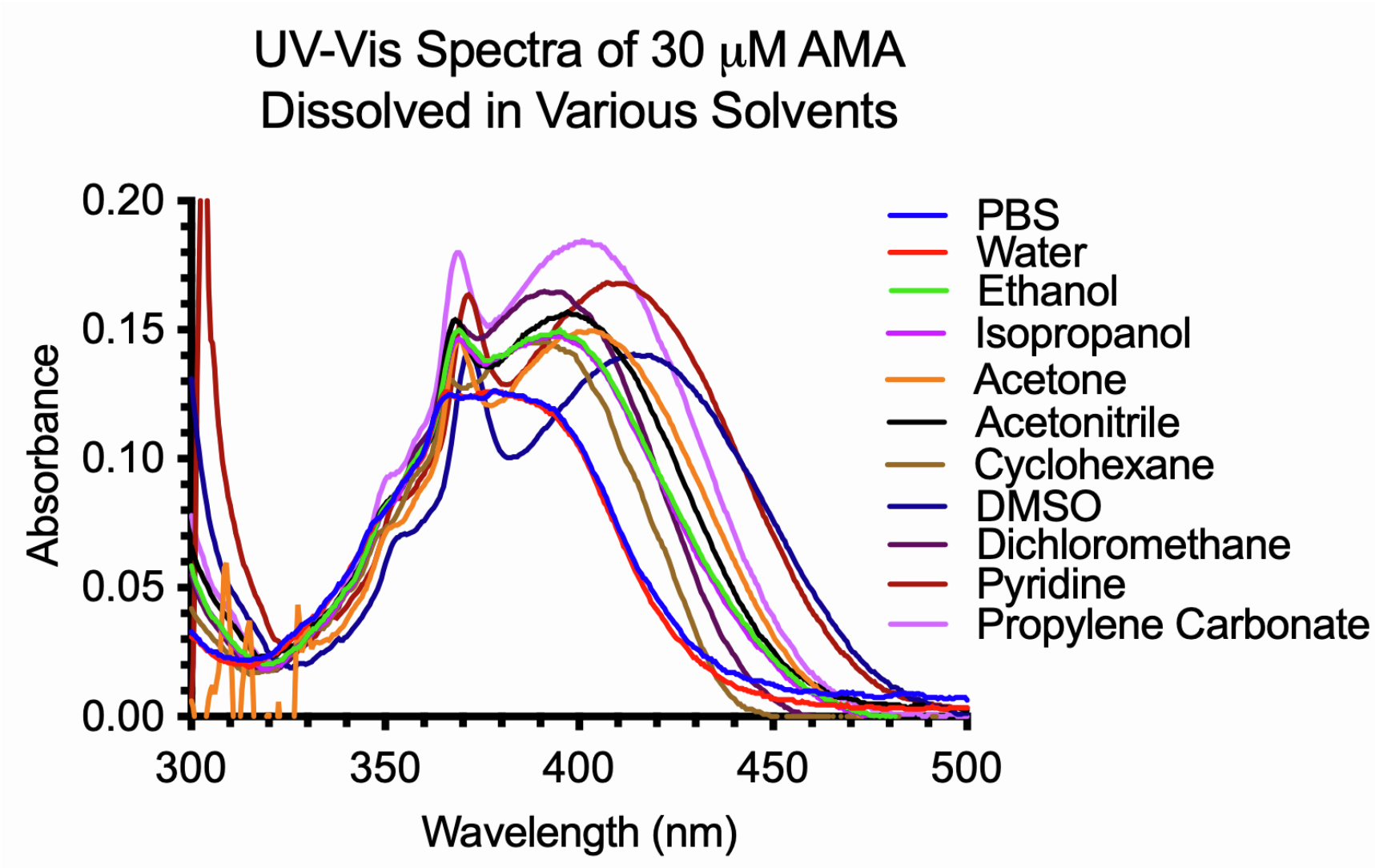

Figure S2. Fluorescence of 100 nM AMA in PBS with different concentrations of DOPC. DOPC was originally dissolved in ethanol, and the final concentration of ethanol in the cuvette was always 0.5%. For reference, the concentration of DOPC where it forms bilayers is ~50 μM (analogous to its critical micellar concentration) (26).

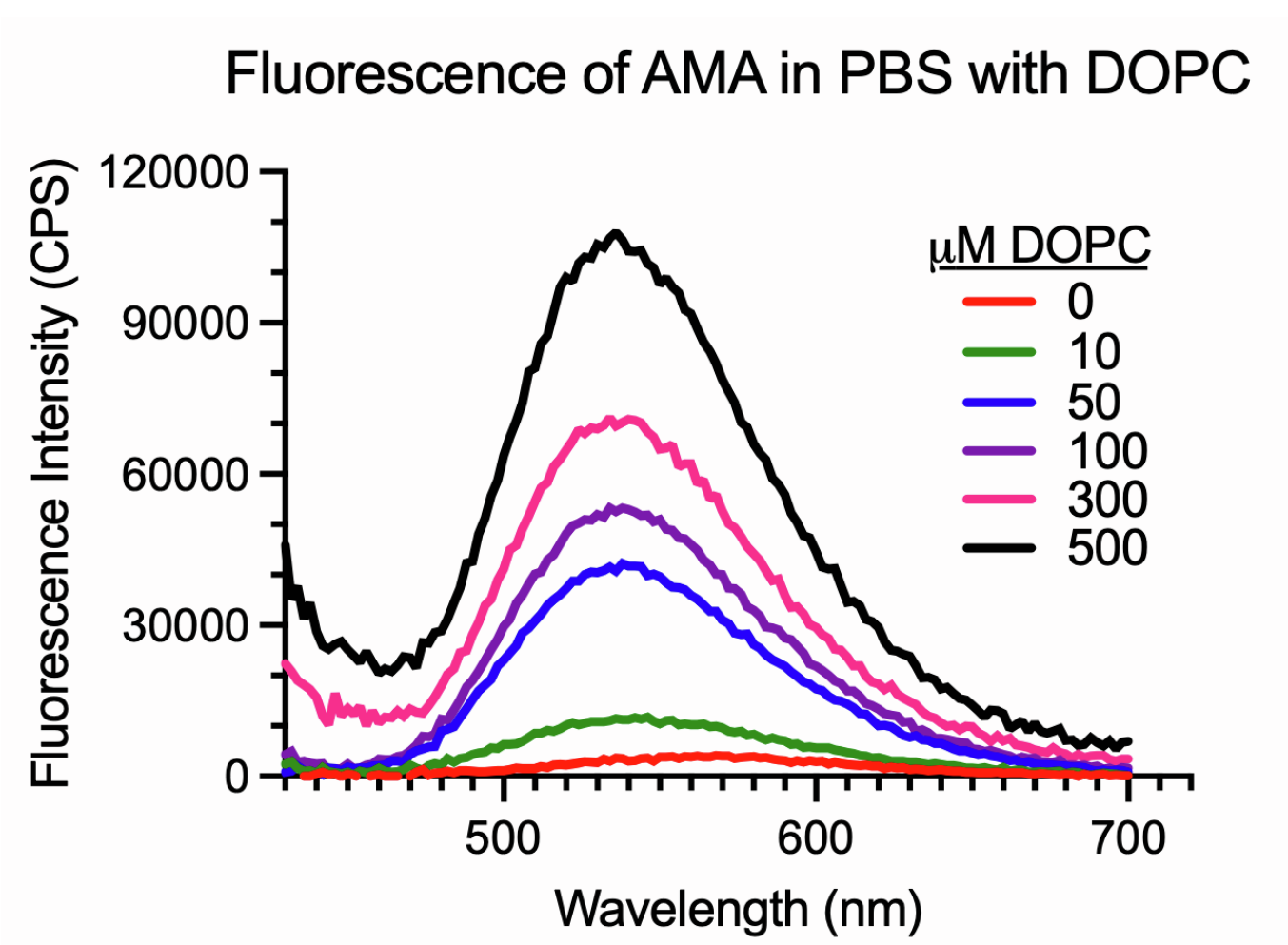

Figure S3. Anesthetics do not compete with AMA for ApoE or pyruvate kinase binding. (A) The fluorescence of 100 nM AMA is shown with 5 μM ApoE and the indicated ligands. (B) The fluorescence of 100 nM AMA is shown with 10 μM pyruvate kinase and the indicated ligands.

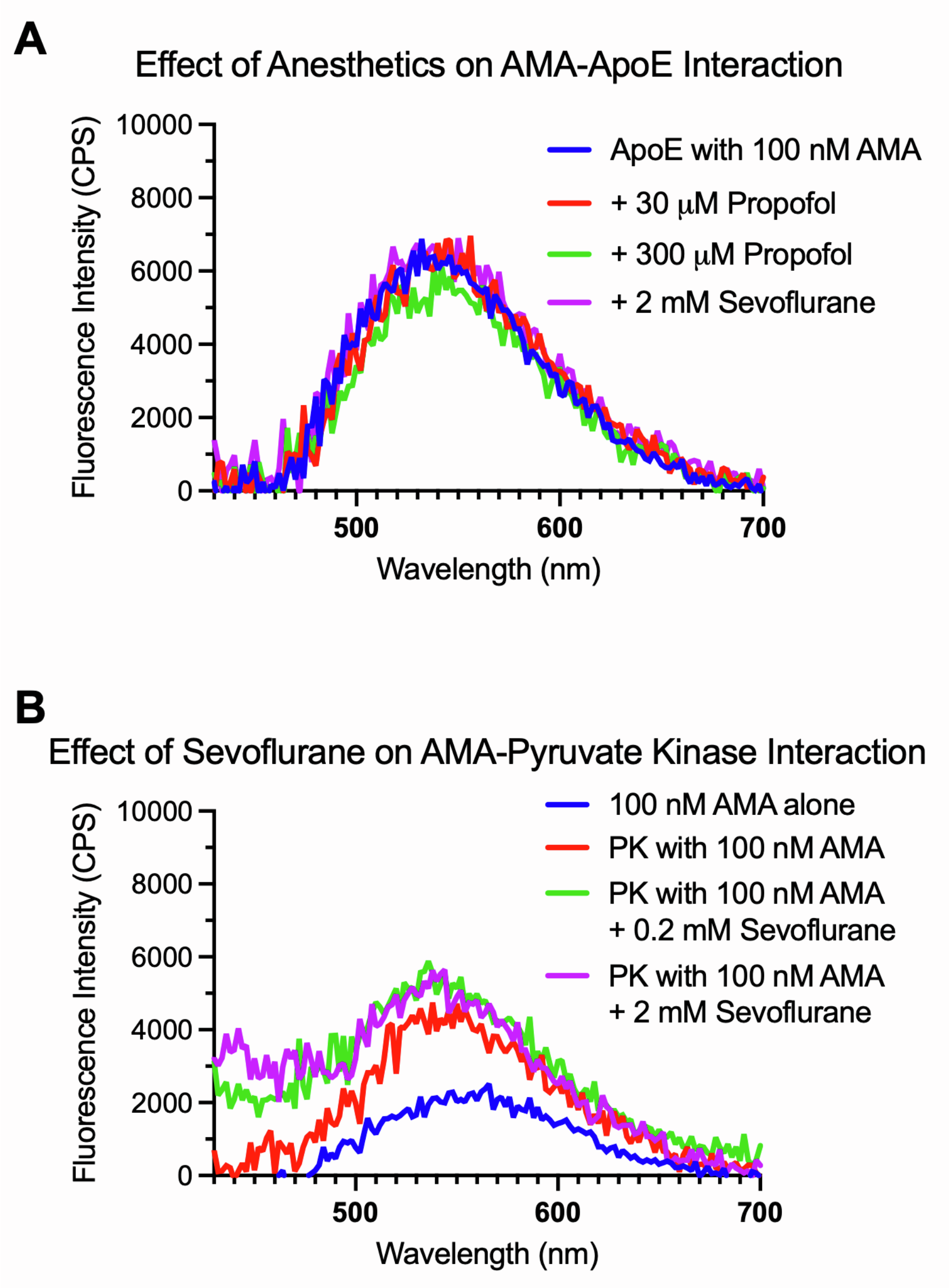

Figure S4. Mass spectra of peptides photolabeled by AziP*m*.

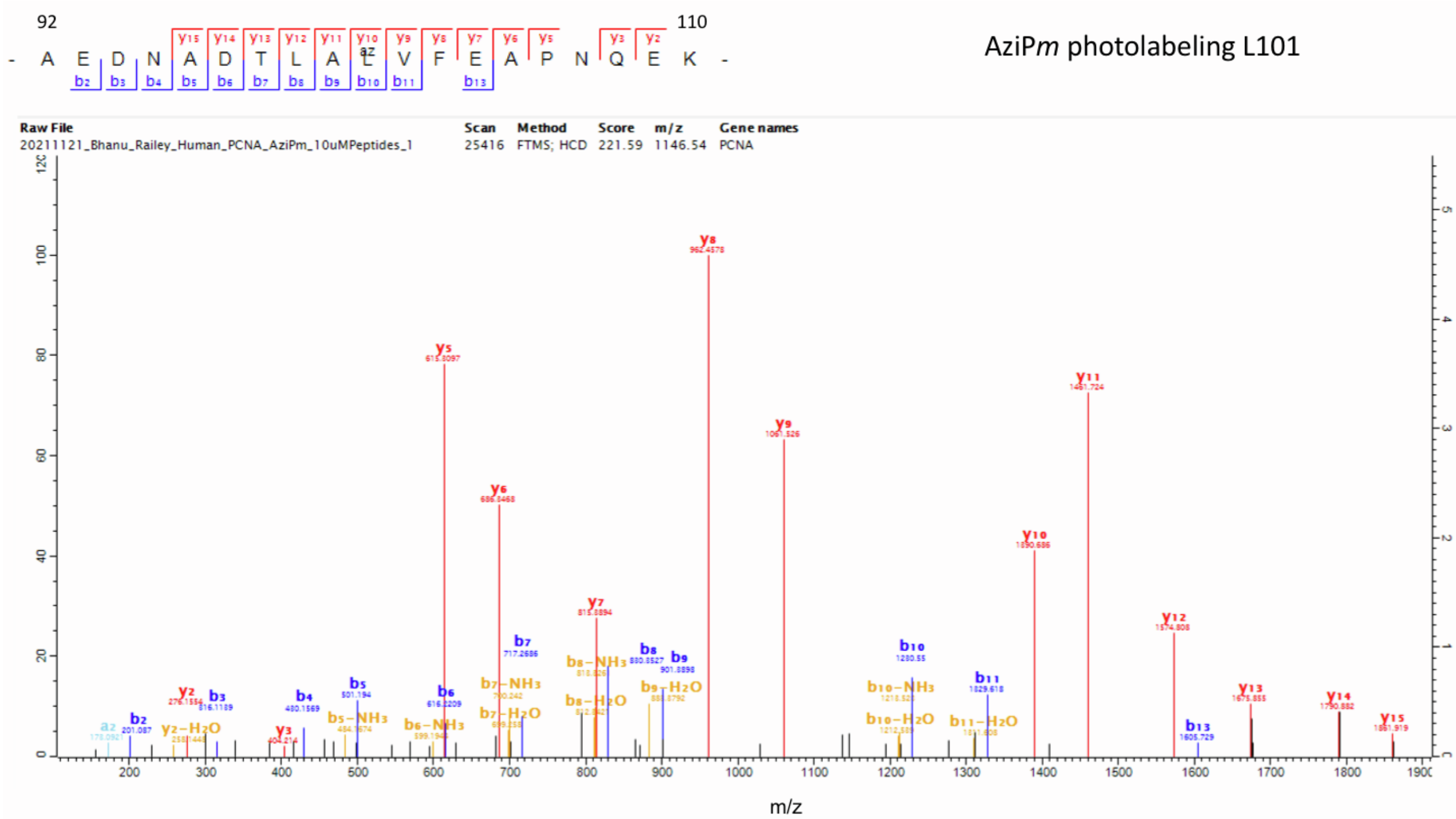

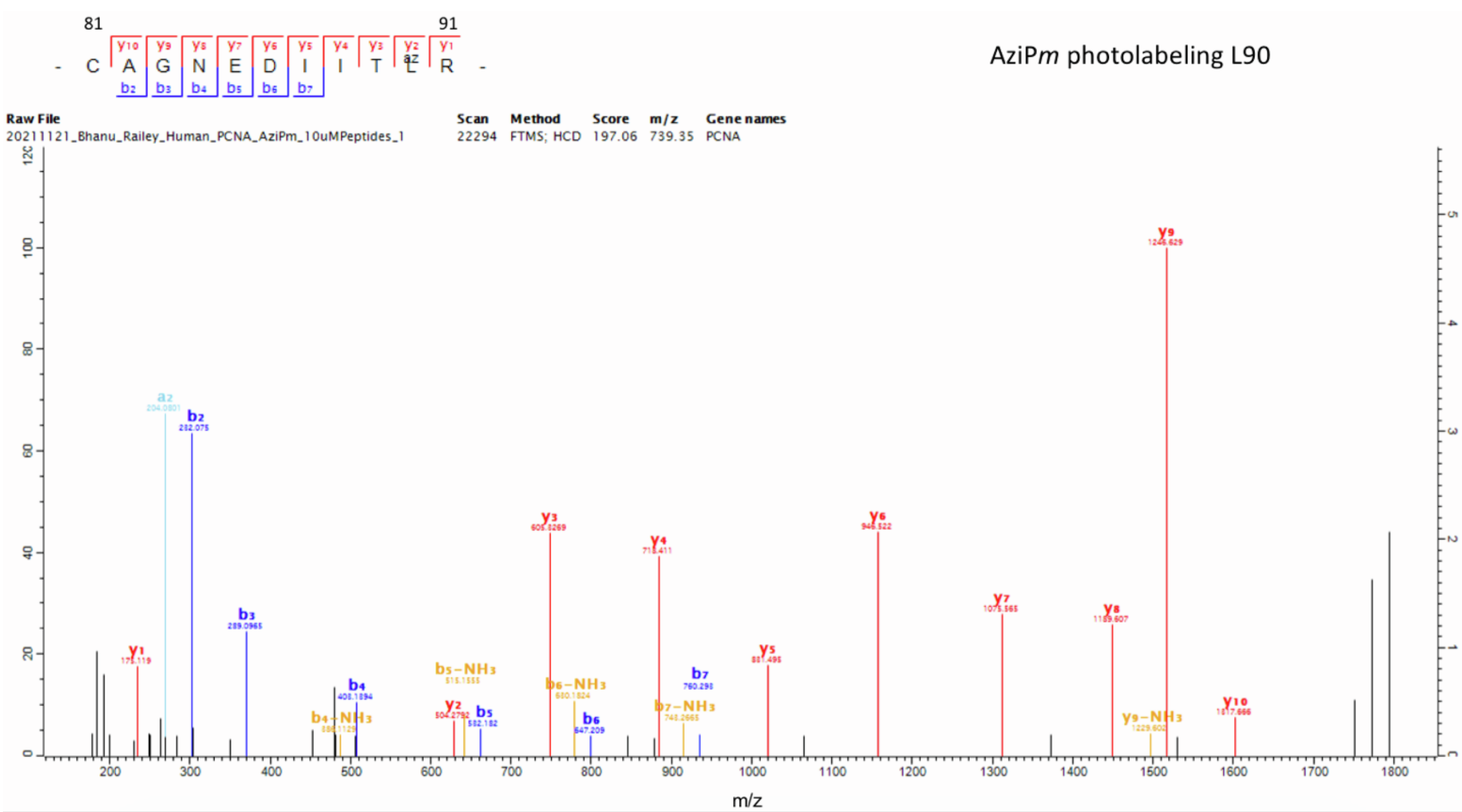

Figure S4. (continued)

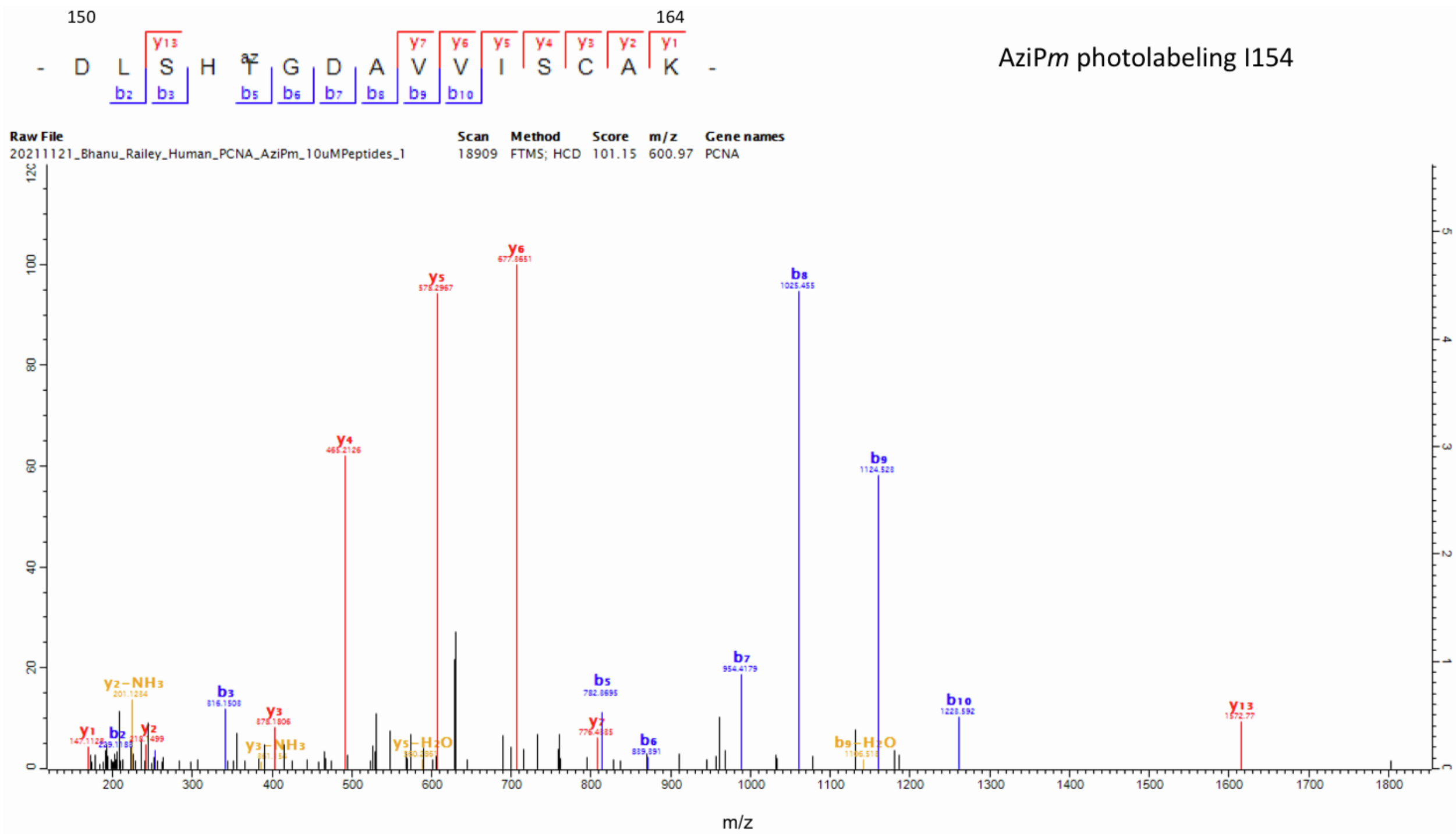

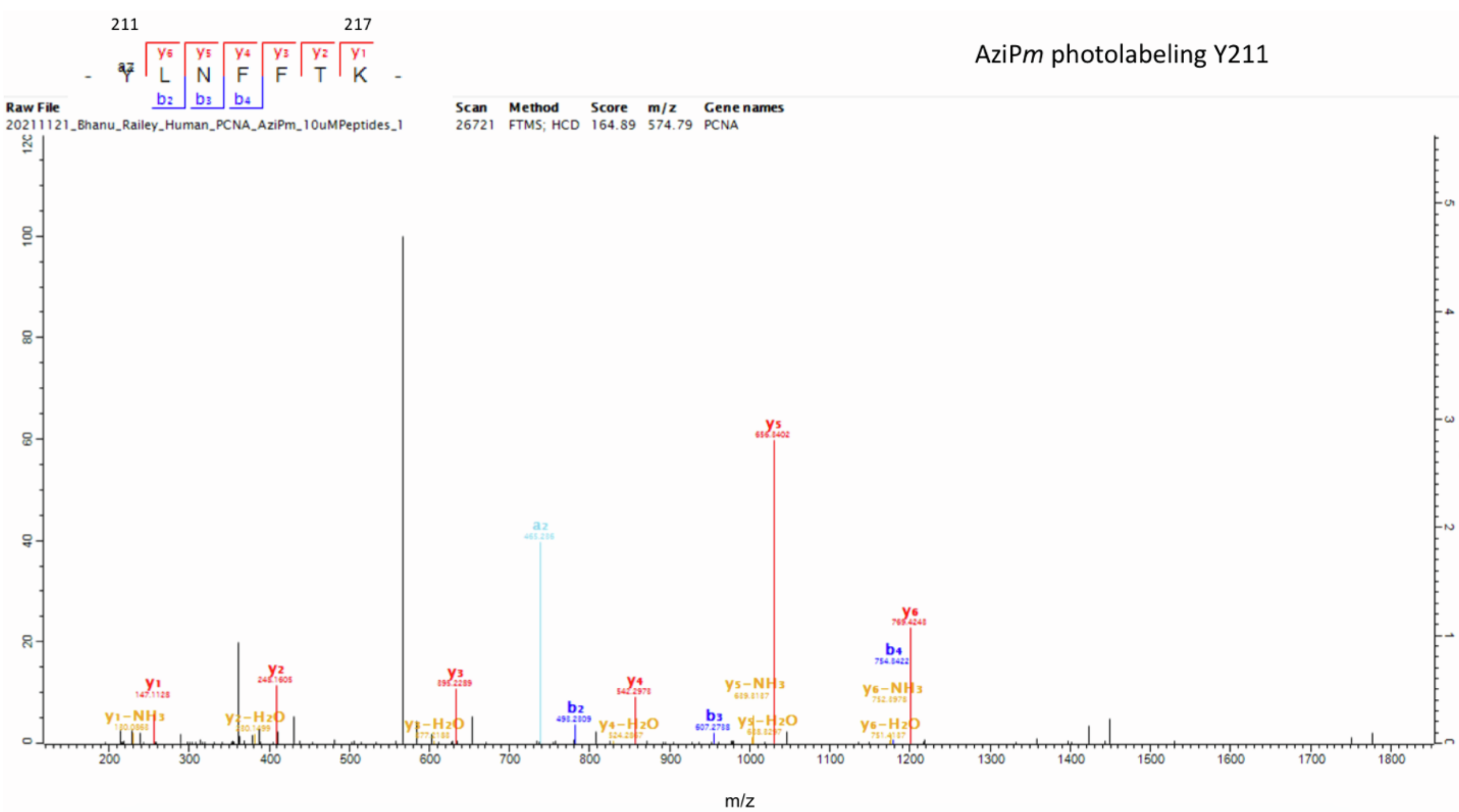

Figure S5. Surface cavity mutations that did not significantly affect AMA binding to PCNA. Y250 and V236 were in surface pockets identified by Fpocket as potential sites for small molecules, but their mutation did not significantly affect AMA binding or its emission peak wavelength.

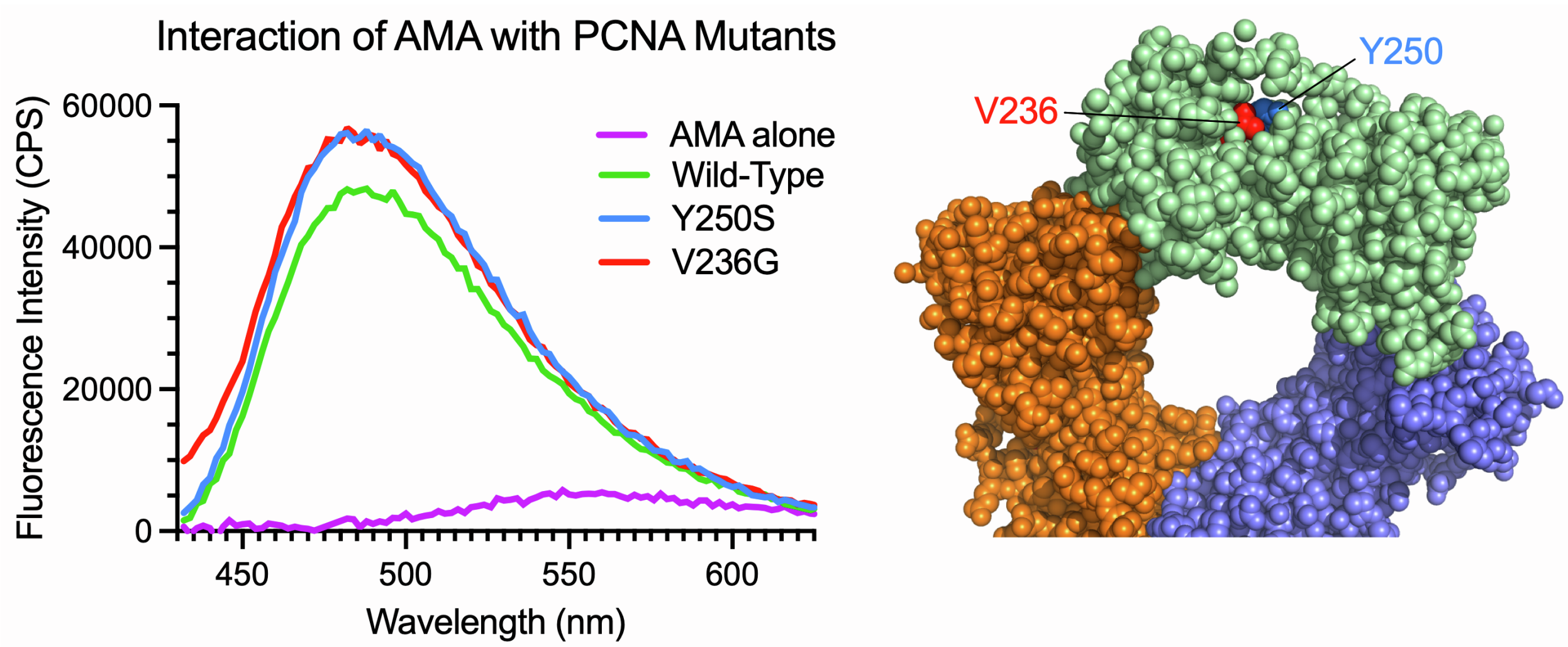

Figure S6. Fpocket analysis of the PCNA crystal structure PDB: 1VYM showing identified cavities in black and residues L90 and L101 as magenta and cyan sticks, respectively. No pockets or cavities were contacting these residues.

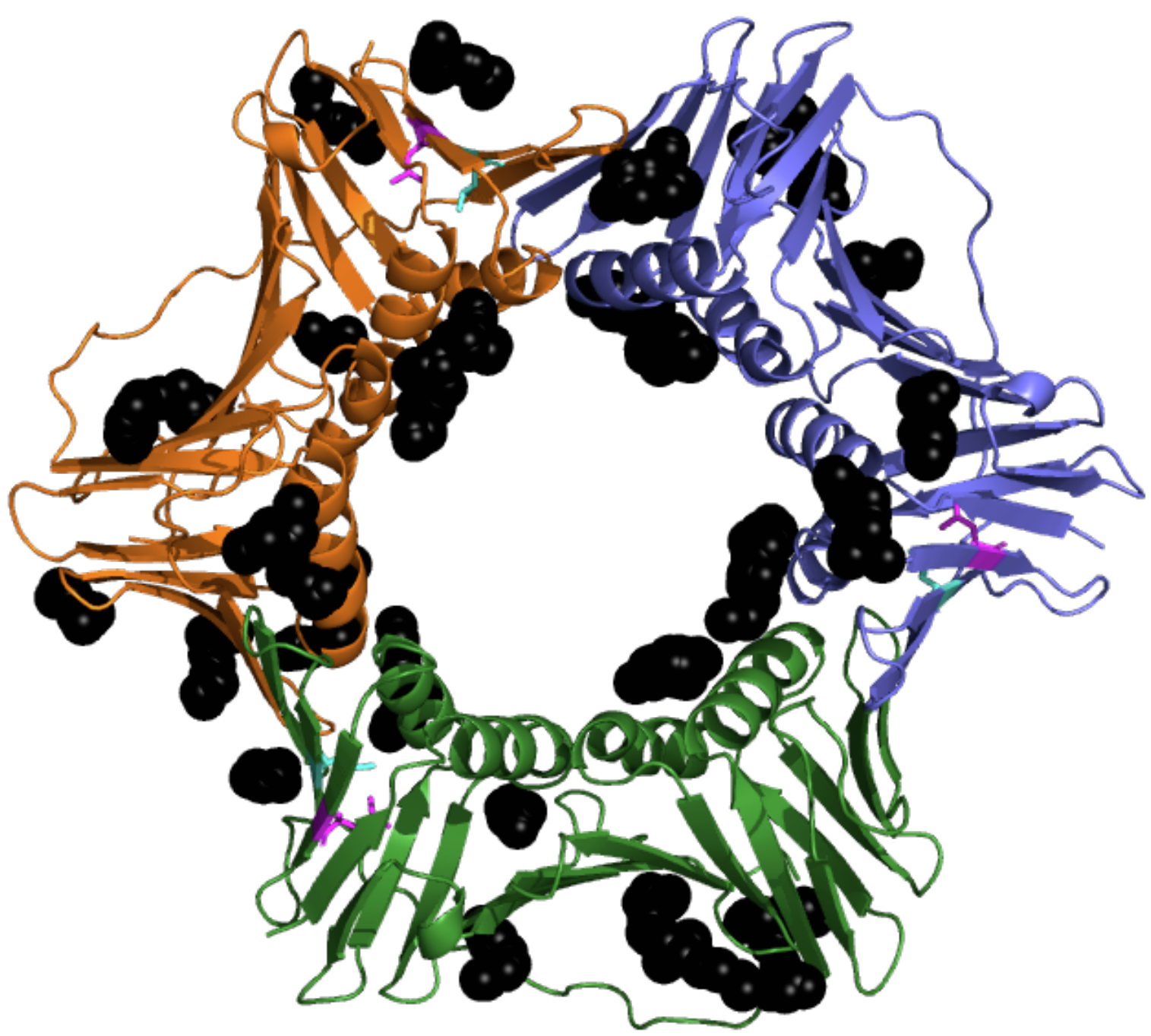

Figure S7. Root mean square deviation (RMSD) of protein backbone atoms throughout atomistic molecular dynamics simulations. The frames were referenced to the initial structure that resembled the crystal structure (PDB: 1VYM) for the 1 μs simulations, or AlphaFold-generated models for the 0.5 μs simulations.

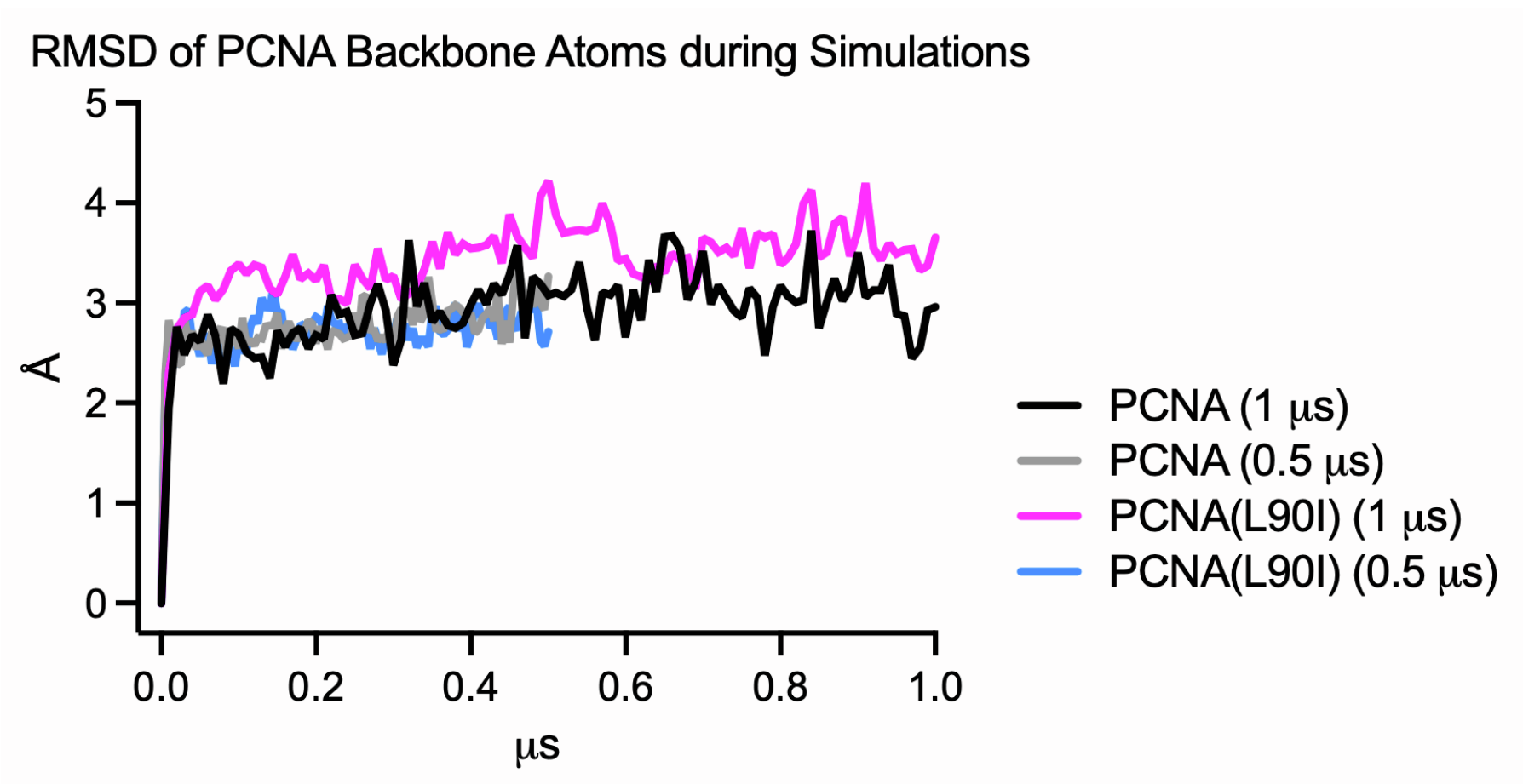

Table S2. Cryptic pocket analysis of wild-type PCNA and PCNA(L90I) from MD simulations. MDpocket analysis of 1 μs simulations was used to identify residues contributing to the cryptic site in subunits 1, 2, and 3 of the PCNA homotrimer. The list of pocket-lining residues were reported below.

Subunit	PCNA Variant	Cryptic Pocket Residues
1	WT	F2, A4, L6, L12, L16, W28, I30, V35, N36, L50, L52, F57, Y60, M68, V70, M75, L79, L90, L99, L101, L118
2	WT	F2, A4, L6, I11, L12, V15, L16, W28, I30, V35, L37, L50, L52, F57, M68, V70, M75, L79, I88, L90, R91, A92, L99, L101, L118
3	WT	F2, A4, L6, Q8, I11, L12, L16, W28, I30, V35, L50, L52, F57, Y60, M68, V70, M75, L79, I88, L90, R91, L99
1	L90I	F2, A4, L12, V15, L16, W28, I30, V35, L50, L52, F57, Y60, L66, M68, V70, L72, S74, M75, I78, L79, I88, I90, A92, L99, L101, M116, K117, L118, L175 from chain B
2	L90I	F2, A4, L6, L12, V15, L16, W28, D29, I30, S31, G34, V35, N36, L50, L52, F57, Y60, M68, V70, M75, L79, I88, I90, L99, L101, L118
3	L90I	F2, A4, L6, Q8, I11, L12, K13, V15, L16, L19, W28, I30, V35, N36, L37, L50, T51, L52, F57, Y60, M68, V70, L72, M75, L79, I88, T89, I90, A92, L99, L101, L118, L221, I241, M244

Figure S8. Pocket volumes in PCNA subunits over time and protein molecular interactions in the cryptic site. (A) Volume of the cryptic site in each of the three PCNA subunits throughout two independent MD simulations. (B) Volume of the cryptic site in each of the three PCNA(L90I) subunits throughout two independent MD simulations. Spearman rank correlations showed no relationship between pocket volumes in different subunits of the same PCNA homotrimer for both wild-type and PCNA(L90I) proteins. Spearman rank correlations were calculated in GraphPad Prism for two subunits at a time (data was non-parametric because pockets < ~25 $Å^3$ were not detected and assigned a volume of 0). (C) Volume of the cryptic site for every analyzed frame in MD simulations. The data for each of the three subunits were combined within the same simulation, and the black bar represents the median (*left* to *right*, 120.55, 136.06, 119.54, 137.57). (D) Top PCNA residue side chains that interacted with L90 or I90 in MD simulations of wild-type PCNA or PCNA(L90I). Each protein had three subunits. Values represent the percent of simulation time that each residue side chain was within 4 Å of L90 or I90. For statistical comparison, values were grouped by interacting residue (e.g., n = 6 per protein for residue 2). Data were analyzed using a two-way ANOVA followed by Sidak's multiple comparisons test in GraphPad Prism. Residues 2, 75, and 99 showed increased interaction with L90 compared to I90 (adjusted p value = 0.0001, 0.0064, and < 0.0001, respectively).

Figure S8. (continued)

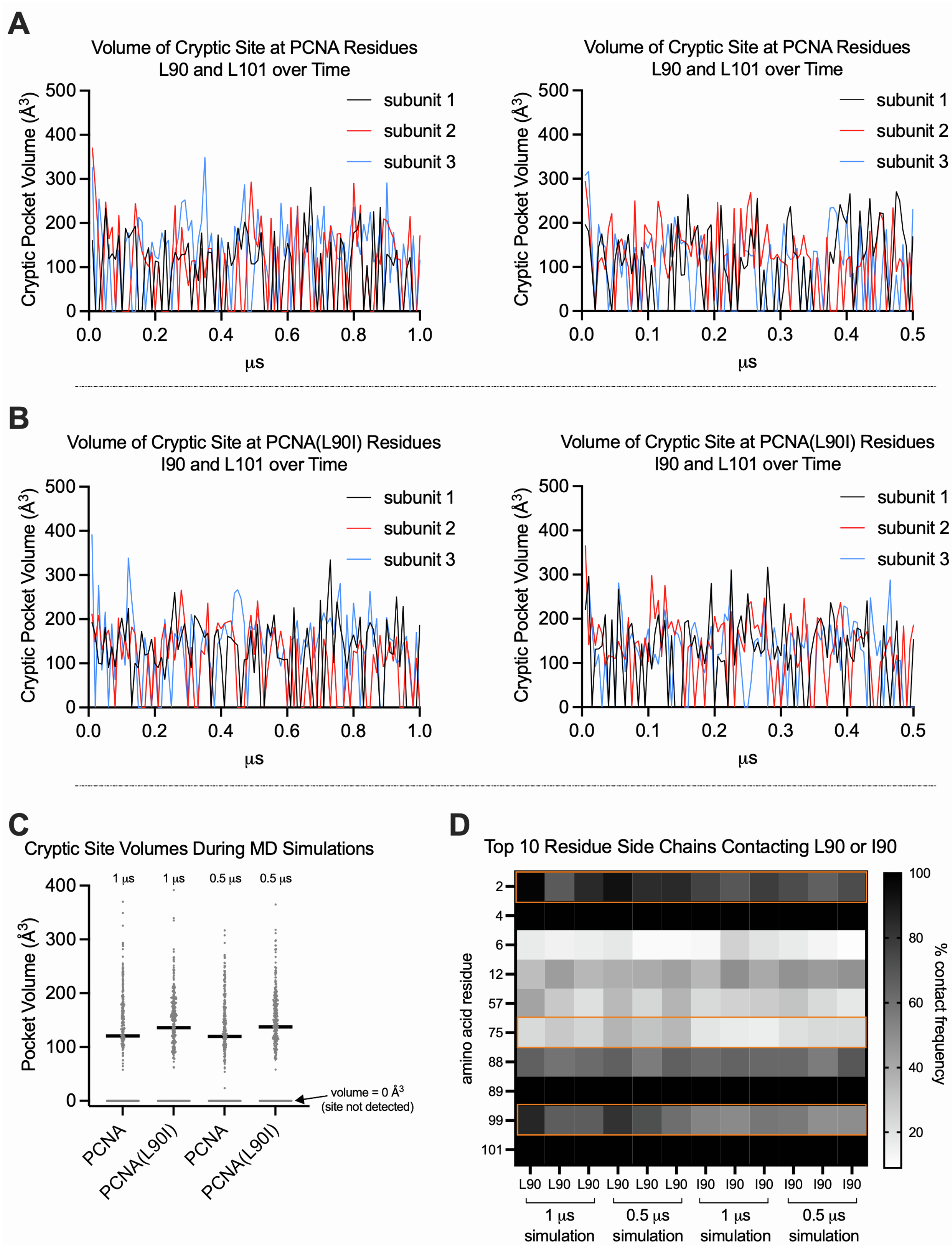

Figure S9. Ligand RMSD during MD simulations and snapshots of AMA unbinding PCNA(L90I). (A) RMSD of three sevoflurane molecules, each bound to a PCNA subunit for 1 μs. RMSD was calculated relative to the starting position of the ligand after aligning the protein backbone throughout the trajectory. Note that RMSD factors ligand translation, rotation, and internal conformational changes. Subunit 1 data is also shown in Video S1. (B) RMSD of three sevoflurane molecules, each bound to a PCNA(L90I) subunit for 1 μs. (C) RMSD of one AMA molecule bound to PCNA or PCNA(L90I) from three separate simulations. The PCNA data is also shown in Video S2. Snapshots from the AMA unbinding trajectory are shown in panel D. (D) Snapshots of AMA unbinding PCNA(L90I). Residues I90 and L101 in the cryptic site are shown as sticks along with residues V70, N71, S74, M116, K117, and M119 at the site exit point; residue L175 is also shown at the exit and is on a different PCNA subunit colored green. The gray blobs were pockets identified by MDpocket on the frames using default parameters. The third PCNA subunit was omitted from the background for clarity. Note that the ligand did not leave through the subunit interface, but instead through an intra-subunit tunnel that formed between I90/L90 and the indicated residues.

Figure S9. (continued)

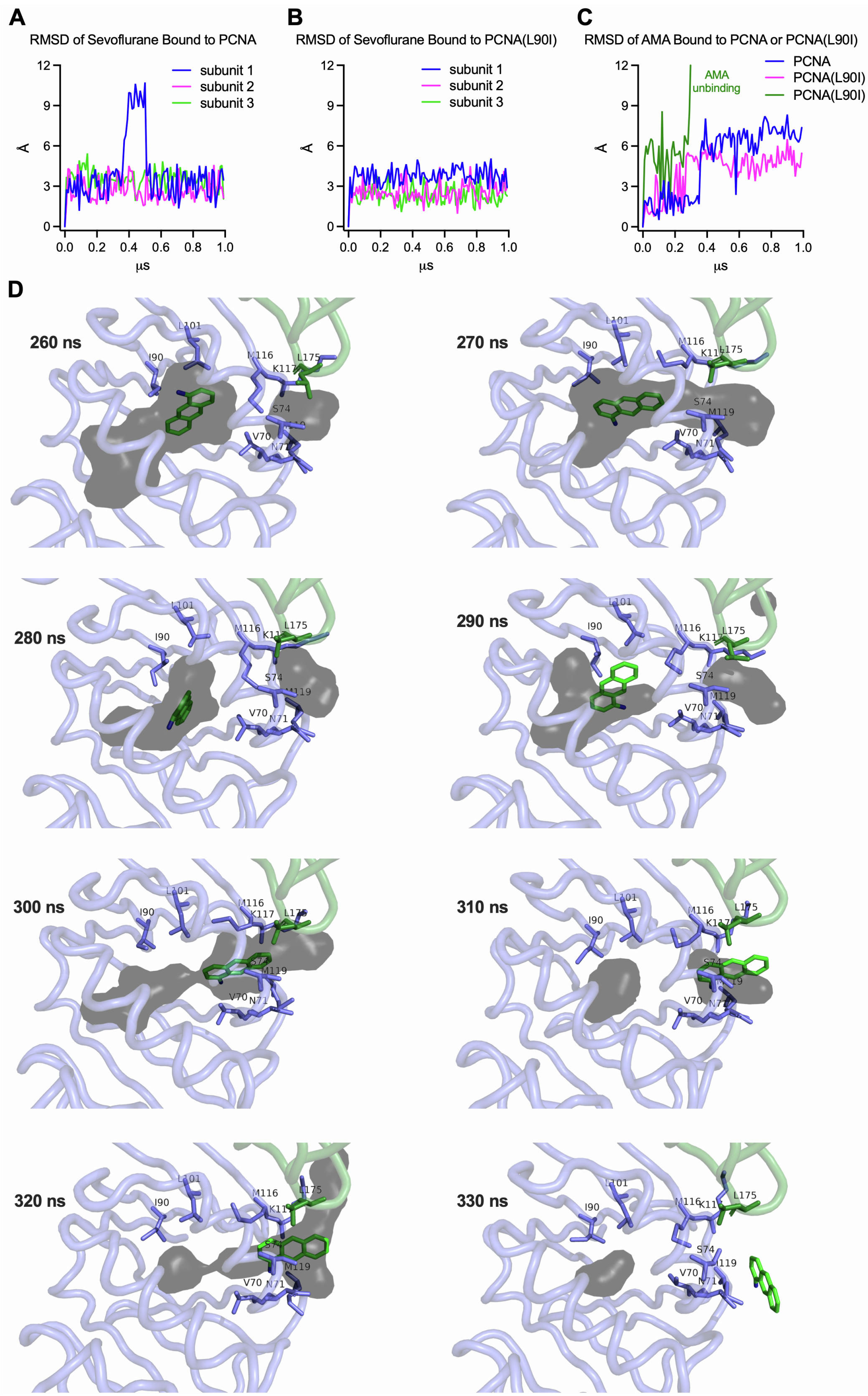

Figure S10. Thermostability of PCNA, PCNA(L90I), PCNA(C148S), or UNG2 in the absence and presence of sevoflurane. (A) The thermal unfolding of 3 µM PCNA protein was measured by recording its intrinsic tryptophan fluorescence at increasing temperatures (3 min holds every 2.5°C increment). When added, the sevoflurane concentration was 2.5 mM, and the DMSO vehicle was always 1%. The curves were fit using a four parameter sigmoidal function (see main text). (B) The thermal unfolding of 3 µM PCNA(L90I) conducted as in panel A. (C) The thermal unfolding of 3 µM UNG2 (uracil DNA glycosylase), which was conducted as above and fit with the four parameter function. For UNG2, we had to constrain the Y_{max} and Y_{min} parameters to 100 and 0, respectively, in order to determine the T_m values. (D) The thermal unfolding of 3 µM PCNA(C148S) conducted as in panel A. (E) Binding isotherm showing the interaction of AMA with recombinant PCNA(C148S). (F) Displacement of AMA from 10 µM PCNA(C148S) by sevoflurane. In this assay, 2 mM sevoflurane displaced 46% of AMA; as a close comparison, we determined that a concentration of 2.2 mM sevoflurane displaces 50% of AMA from wild-type PCNA (see Figure 3 in the main text).

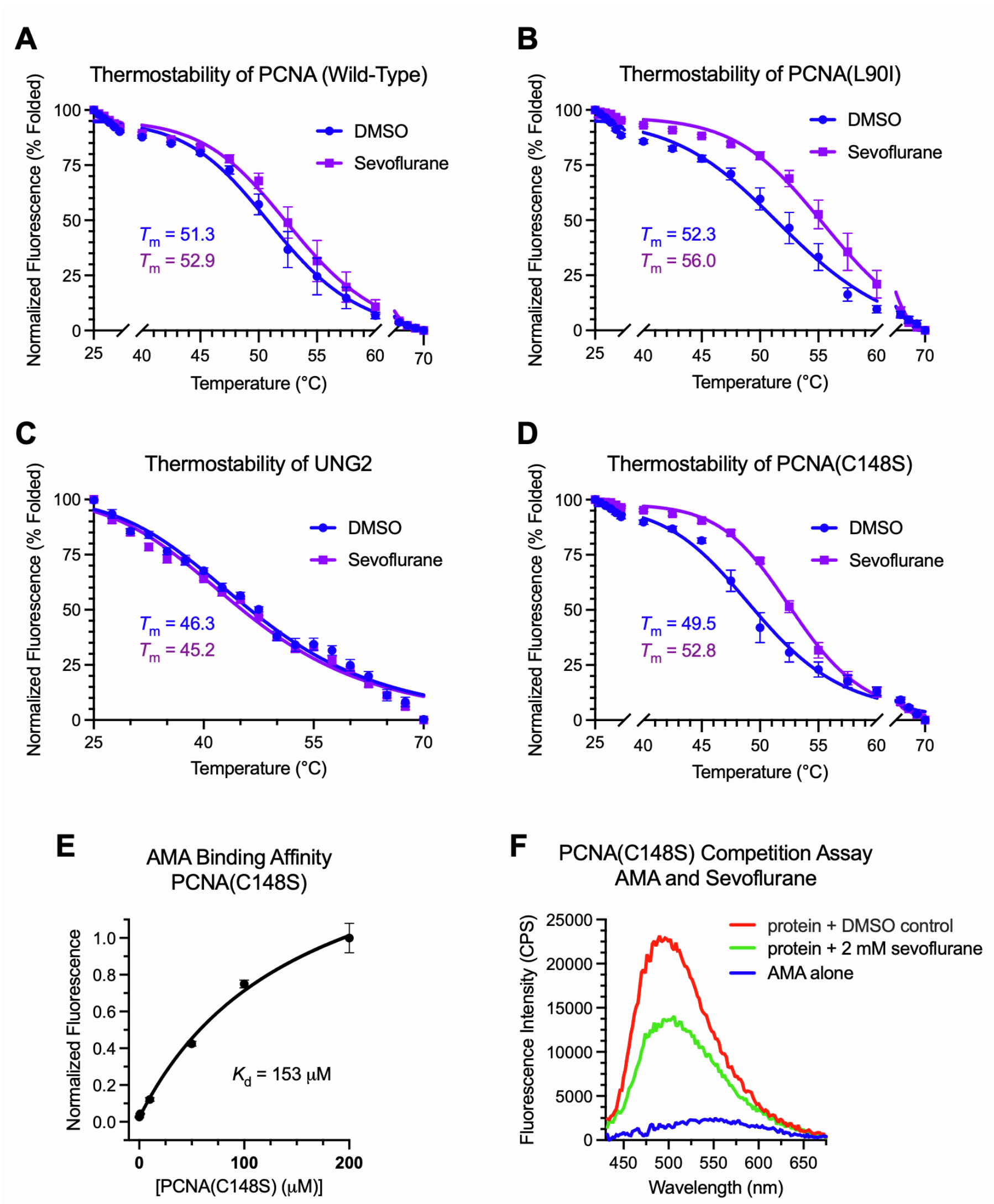

Figure S11. Anesthetic binding to the PCNA cryptic site does not affect interactions at the interdomain connecting loop (IDCL) site. (A) Sevoflurane did not significantly disrupt the interaction between PCNA and a fluorescein-labeled full-length UNG2 protein called UNG2(fluor) (27). In this assay, 50 nM UNG2(fluor) was complexed with 5 μM PCNA. If sevoflurane prevented their interaction, the fluorescence anisotropy of fluorescein-labeled UNG2 would reduce toward the free UNG2(fluor) baseline (27). (B) Propofol did not efficiently disrupt binding between PCNA and fluorescein-labeled UNG2. This experiment was conducted as in panel A. (C) The affinity (K_d) of PCNA for a fluorescein-labeled peptide called Pogo-Ligase was determined in the presence and absence of 100 μM propofol, which did not significantly affect the protein-peptide interaction. Methods for this assay can be found elsewhere (27, 28). (D) The affinity (K_d) of PCNA for a fluorescein-labeled p15 peptide was determined in the presence and absence of 2 mM sevoflurane, which did not significantly affect the protein-peptide interaction. This fluorescence anisotropy assay was performed with the same method as the Pogo-Ligase binding assay (27, 28), except that we used a peptide purchased from Biosynth with a p15 sequence instead (6FAM-dPEG4-AGGNPVCVRPTPKWQKGIGEFFRLS).

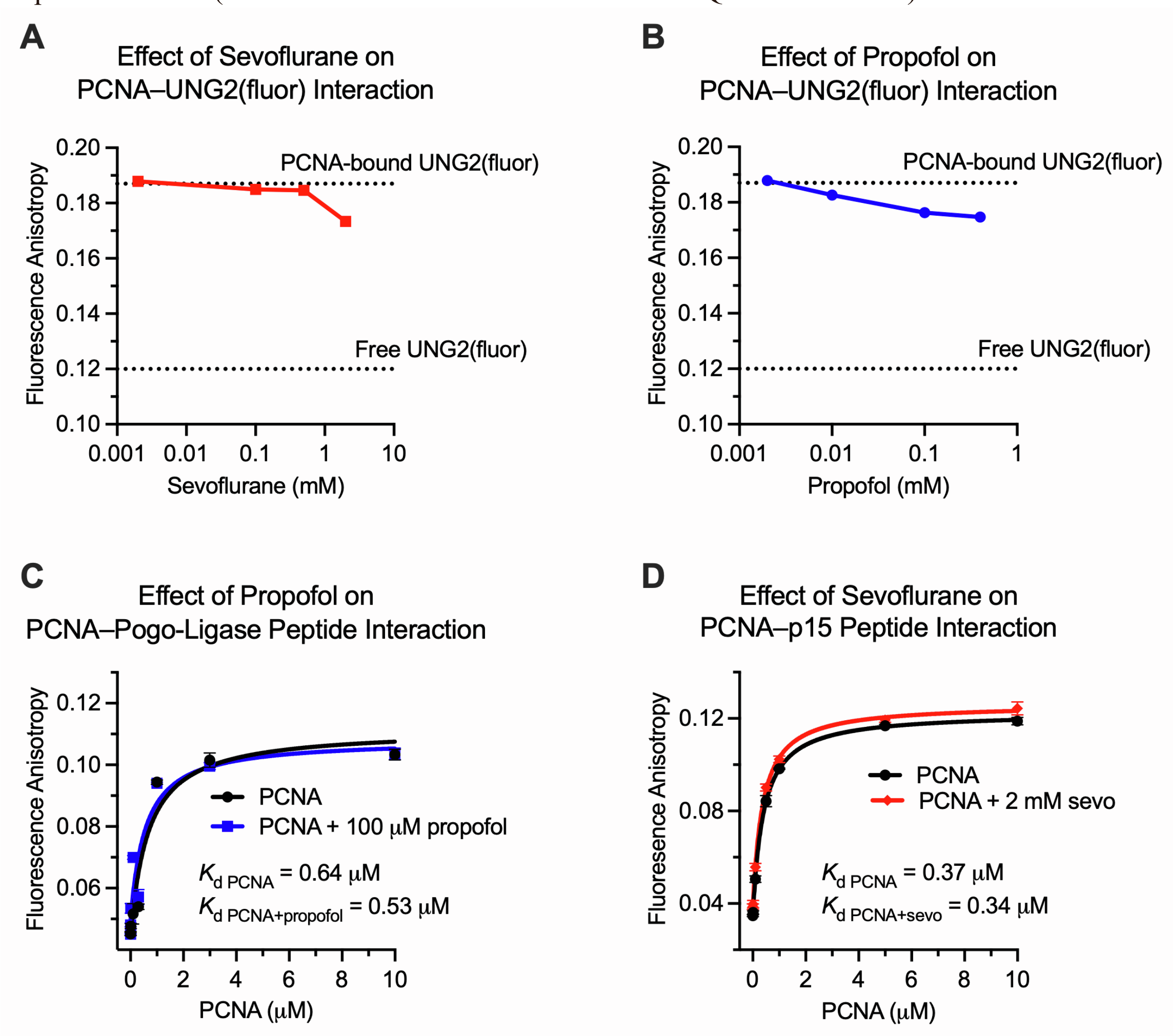

Figure S12. Sevoflurane did not affect loading of PCNA onto DNA by RFC. (A) Continuous timepoint fluorescence data from RFC-catalyzed ATP hydrolysis assays, which coupled ATP hydrolysis to NADH oxidation, which was observed by fluorescence (see Methods beneath the figure). 150 nM RFC was stimulated by 1 μM primer-template DNA, even more so in the presence of 1 μM PCNA, as previously reported (29). DMSO vehicle and 2.5 mM sevoflurane did not have a significant effect on ATP hydrolysis rate (i.e., the slope of the traces in panel A). (B) Rate constants (k_{obs}) for RFC activity from the ATP hydrolysis assays shown in panel A.

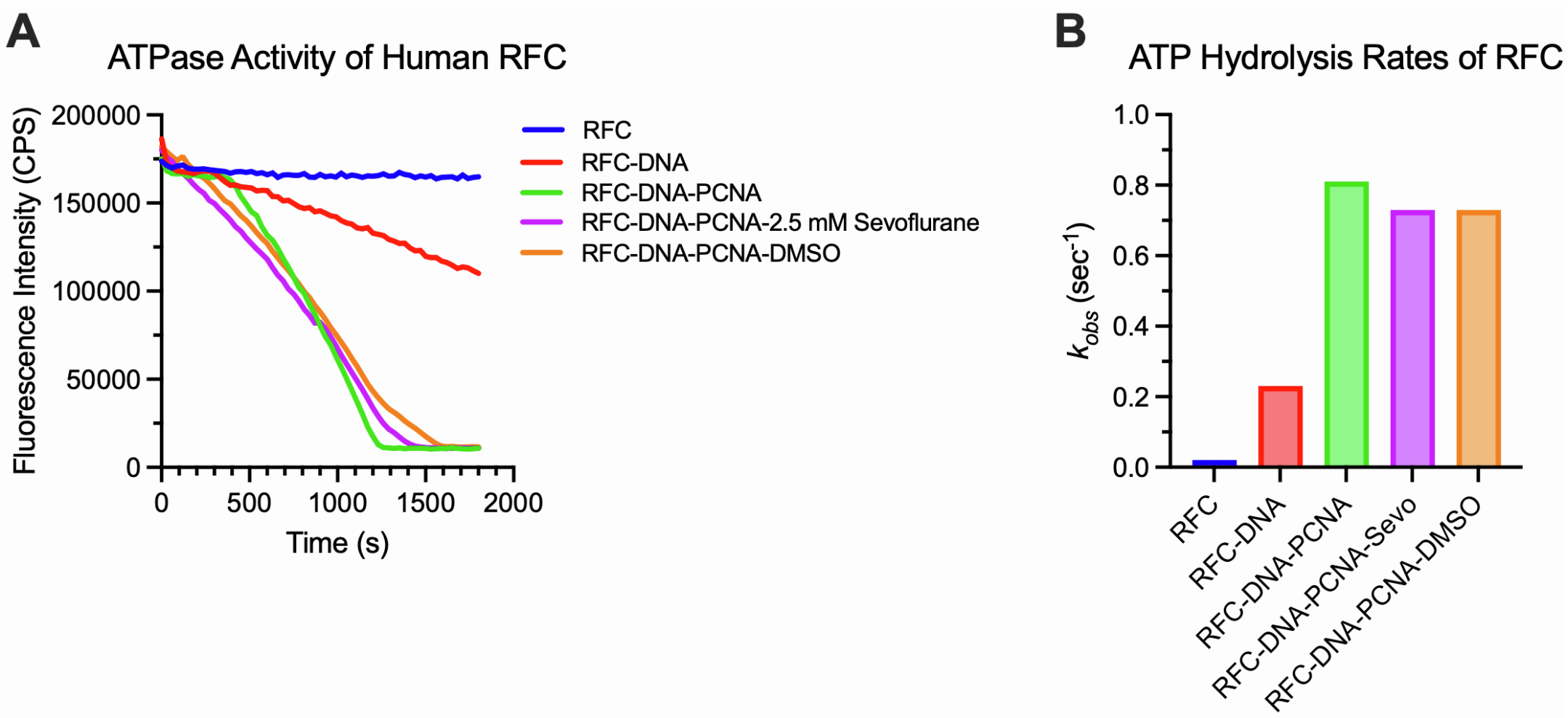

RFC-catalyzed ATP hydrolysis was monitored using an NADH-coupled enzyme assay that links ATP hydrolysis to NADH oxidation via pyruvate kinase and lactate dehydrogenase (29). Recombinant human ΔN555-RFC was a generous gift from Dr. Brian Kelch (University of Massachusetts). Reactions were performed in assay buffer containing 50 mM Tris (pH 7.5), 5 mM $MgCl_2$, 500 μM TCEP, 200 mM potassium glutamate, 1 mM ATP, 670 μM phosphoenolpyruvate, 170 μM NADH, 3 U/mL pyruvate kinase, and 3 U/mL lactate dehydrogenase. Reactions (200 μL, 23°C) containing 1 μM PCNA and 1 μM primer-template DNA oligonucleotide were initiated by addition of 150 nM RFC. NADH fluorescence was measured continuously (λ_{ex} = 350 nm, λ_{em} = 460 nm) using a Horiba Fluoromax-4 instrument. The decrease in NADH fluorescence corresponds stoichiometrically to ATP hydrolysis and was plotted against time (s) to calculate observed rates (K_{obs}) of RFC activity using the equations

$$Rate = \frac{m*[NADH]}{Y_{max}-Y_{min}} \qquad Rate\ Constant\ (k_{obs}) = \frac{Rate}{[RFC]}$$

where *m* was the slope obtained from a simple linear regression fit of the data in GraphPad Prism, [NADH] was the initial NADH concentration, Y_{max} was the maximum values of NADH fluorescence intensity in the absence of RFC, Y_{min} was the minimum plateau after complete ATP hydrolysis by RFC, and [RFC] was the RFC concentration in the assay.

<u>Primer-template oligonucleotide sequences (annealed for the assays)</u>
5'-ATCGAGCTGGACACCATCACTCCATATTGGTCACTTAGCTATCGATTCCCGCATGC TCTTAGCTATCGATTCACCTGAGACTAGTCA-3'

5'-TGACTAGTCTCAGGTGAATCGAUAGCTAAGAGCATGCGGGAATCGAU AGCTAAGT-3'

Figure S13. Relationship between pocket volumes at the L90/L101 site and the resolution of experimental data. (A) A correlation was observed between subunit pocket volumes and structural resolution for human PCNA structures. (B) No correlation was observed between subunit pocket volumes and structural resolution for yeast PCNA structures. Spearman rank correlations were calculated in GraphPad Prism.

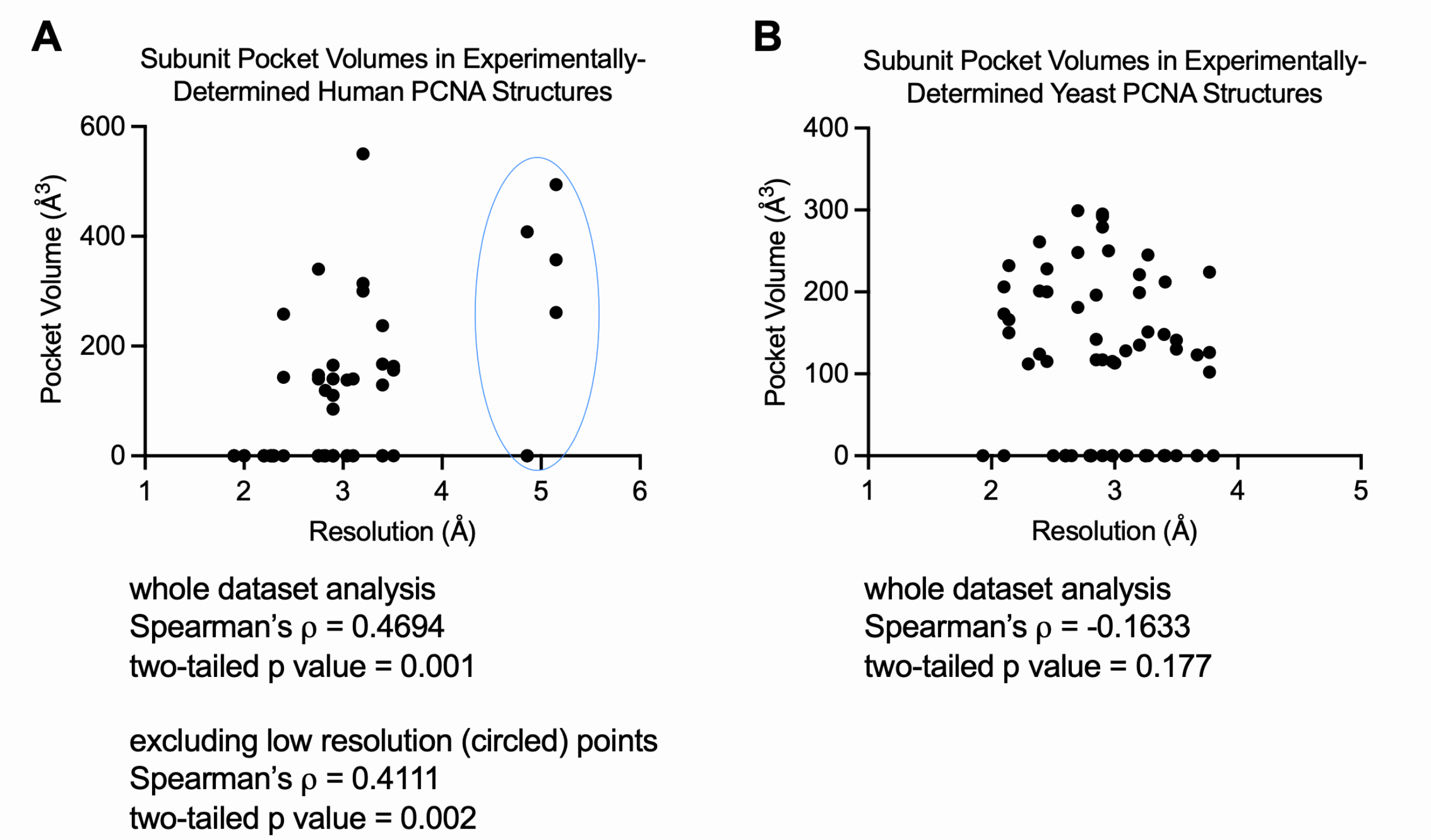

Table S3. Hydrophobic pocket volumes at sites analogous to the PCNA L90/L101 site in various PCNA and β clamp proteins.

PDB	Description	Chain A	Chain B	Chain C	Link
		**Pocket Volume (Angstroms^3)** (not detected assigned value of 0; blank = less than 3 subunits in asymmetric unit)			
**human structures**					
1VYM	Native human crystal; Xray; 2.3 angstroms; apo	0	0	0	https://doi.org/10.1073/pnas.0406540102
6GWS	Human PCNA bound to three p15 peptides; xray; 2.9 angstroms	110	0	0	https://doi.org/10.1093/nar/gky723
6EHT	Human PCNA bound to two p15 peptides and dsDNA; xray, 3.2 angstroms	300	550	314	https://doi.org/10.1093/nar/gky723

5MOM	Human PCNA-S228I ; mutant with IDCL impaired binding and impaired DNA repair; xray; 2.27 angstroms	0	0	0	https://doi.org/10.1016/j.dnarep.2016.12.003
6GIS	Human PCNA bound to dsDNA; xray, 2.82 angstroms	0	119	0	https://doi.org/10.1038/ncomms13935
3TBL	Human monoubiquinated PCNA ; 2 ubiquitins; xray; 2.9 angstroms	140	0	165	https://doi.org/10.4161/cc.20595
1UL1	Human PCNA bound to three FEN1 proteins; xray 2.9 angstroms	0	85	0	https://doi.org/10.1038/sj.emboj.7600519
3WGW	Human PCNA bound to small molecule IDCL inhibitor; xray 2.8	0			https://doi.org/10.1074/jbc.M113.520429
8E84.	Human PCNA C148S; xray 3.10	0	0	140	https://doi.org/10.1016/j.jbc.2023.104656
8UII	Cryo-EM of human clamp-clamp loader ATAD5-RFC-closed PCNA complex in intermediate state 1; cryo-EM 3.04	138	0	0	https://doi.org/10.1038/s41594-024-01332-4
6S1N	Human polymerase delta holoenzyme Conformer 2; cryo-EM 4.86	408	0	0	https://doi.org/10.1038/s41467-020-14898-6
6VVO	Structure of the human clamp loader (Replication Factor C, RFC) bound to the sliding clamp; cryo-EM 3.40	167	0	0	https://doi.org/10.1101/2020.02.18.953257
7KQ0	human PCNA bound to peptide mimetic; xray 2.40	143	258	0	https://doi.org/10.1016/j.jbc.2021.100773
8YJZ	Structure of the human endogenous PCNA-FEN1-RNase H2 complex - State D; cryo-EM 5.15	261	494	357	https://doi.org/10.1038/s44318-024-00296-x

6CBI	PCNA in complex with inhibitor; xray 2.75	0	0	0	https://doi.org/10.1002/chem.201801734
4RJF	Crystal structure of the human sliding clamp at xray 2.0 angstrom resolution	0	0	0	https://doi.org/10.1021/acs.biochem.5b00241
8UMV	human CTF18-RFC-PCNA-DNA ternary complex with narrow PCNA opening state I (state 5); cryo-EM 2.75	340	140	147	https://doi.org/10.1073/pnas.2319727121
5YCO	Complex structure of PCNA with UHRF2; xray 2.2	0	0	0	https://doi.org/10.1016/j.bbrc.2017.09.102
7NV0	Human Pol Kappa holoenzyme with wt PCNA; cryo-EM 3.4	237	0	129	https://doi.org/10.1038/s41467-021-26251-6
8F5Q	Crystal structure of human PCNA in complex with the PIP box of FBH1; xray 1.9	0	0	0	https://doi.org/10.1016/j.str.2023.03.004
8YJL	Structure of the human endogenous PCNA-FEN1 complex - State B; cryo-EM 3.51	163	156	0	https://doi.org/10.1038/s44318-024-00296-x
**yeast structures**					-
8GLA	Co-crystal structure of caPCNA bound to the AOH1996 derivative, AOH1996-1LE; xray 3.77	224	102	126	https://doi.org/10.1016/j.chembiol.2023.07.001
8GL9	Co-crystal structure of caPCNA bound to AOH1160 derivative 1LE; xray 2.81	0	0	0	https://doi.org/10.1016/j.chembiol.2023.07.001
1PLQ	apo yeast PCNA; xray 2.3	112			https://doi.org/10.1016/0092-8674(94)90014-0
2OD8	yeast PCNA bound to CDC9 PIP peptide; saccharomycese cerevisiae;xray 2.8	0			https://doi.org/10.1093/nar/gkm006
1SXJ	yeast PCNA bound to yeast RFC (no DNA), PCNA ring closed;	117	142	196	https://doi.org/10.1038/nature02585

	saccharomycese cerevisiae; xray 2.85				
7TFH	yeast PCNA bound to yeast RFC and DNA; PCNA ring closed; saccharomycese cerevisiae; cryo EM 3.09	0	0	128	https://doi.org/10.7554/eLife.77469
7TI8	yeast PCNA bound to RFC only, no DNA, ring open; saccharomycese cerevisiae; cryo EM 3.5	0	130	141	https://doi.org/10.7554/eLife.74175
7TIB	yeast PCNA ring open bound to RFC with primer-template DNA; saccharomycese cerevisiae; cryo EM 3.4	0	148	0	https://doi.org/10.7554/eLife.74175
8DQX	open state of RFC:PCNA bound to a 3' ss/dsDNA junction; saccharomycese cerevisiae; cryo EM 2.1	0	206	173	https://doi.org/10.7554/eLife.78253
3GPN	non-trimeric form of the yeast E113G PCNA mutant protein; saccharomycese cerevisiae; xray 2.5	0			https://doi.org/10.1107/S0907444909011329
3F1W	yeast PCNA mutant G168S; mutant blocks translesion synthesis; saccharomycese cerevisiae; xray 2.9	117			https://doi.org/10.1021/bi8017762
7TFI	yeast PCNA bound to yeast RFC and DNA; PCNA ring open; saccharomycese cerevisiae; cryo EM 3.41	0	212	0	https://doi.org/10.7554/eLife.77469
5V7M	PCNA mutant L126A/I128A Protein Defective in Gene Silencing; Saccharomyces cerevisiae S288C; xray 1.93	0			https://doi.org/10.1371/journal.pone.0193333

5T9D	Structure of PCNA acetylated on K20; Saccharomyces cerevisiae S288C; xray 3.27	245	151	0	https://doi.org/10.2210/pdb5T9D/pdb
3K4X	Saccharomyces cerevisiae Sliding Clamp PCNA Bound to DNA; xray 2.98	115	0	0	https://doi.org/10.1186/1472-6807-10-3
8THW	Cac1 PIP motif bound to Saccharomyces cerevisiae PCNA; xray 2.6	0	0	0	https://doi.org/10.1016/j.jmb.2024.168695
3V60	Structure of S. cerevisiae PCNA conjugated to SUMO on lysine 164; xray 2.6	0			https://doi.org/10.1038/nature10883
3L0W	Structure of split monoubiquitinated PCNA with ubiquitin in position two; xray 2.8	0			https://doi.org/10.1038/nsmb.1776
3V62	S. cerevisiae Srs2 C-terminal domain in complex with PCNA conjugated to SUMO on lysine 164; xray 2.9	0			https://doi.org/10.1038/nature10883
8THC	Structure of the Saccharomyces cerevisiae clamp unloader Elg1-RFC bound to a cracked PCNA; cryo-EM 3.67	123	0	0	https://doi.org/10.1126/sciadv.adl1739
8THD	Structure of the Saccharomyces cerevisiae clamp unloader Elg1-RFC bound to PCNA; cryo-EM 3.25	0	0	0	https://doi.org/10.1126/sciadv.adl1739
8TWB	Structure of S. cerevisiae Ctf18-RFC-PCNA-DNA complex; cryo-EM 3.2	199	221	135	https://doi.org/10.1126/science.adk5901
8DR1	Consensus closed state of RFC:PCNA bound to a 3' ss/dsDNA junction (DNA2); xray 2.14	232	150	166	https://doi.org/10.7554/eLife.78253

8DR4	Open state of RFC:PCNA bound to a 3' ss/dsDNA junction (DNA2) without NTD; cryo-EM 2.45	228	200	115	https://doi.org/10.7554/eLife.78253
8DR6	Closed state of RFC:PCNA bound to a nicked dsDNA; cryo-EM 2.39	124	201	261	https://doi.org/10.7554/eLife.78253
8DR7	Open state of RFC:PCNA bound to a nicked dsDNA; cryo-EM 2.7	181	248	299	https://doi.org/10.7554/eLife.78253
4YHR	Crystal Structure of Yeast Proliferating Cell Nuclear Antigen; Saccharomyces cerevisiae S288C; xray 2.95	250			https://doi.org/10.2210/pdb4YHR/pdb
6XC3	S179T Mutant of Saccharomyces cerevisiae S288C PCNA; xray 3.10	0			https://doi.org/10.1371/journal.pone.0157023
3L0X	Structure of split yeast PCNA; Saccharomyces cerevisiae; xray 3.0	113			ttps://doi.org/10.1038/nsmb.1776
6W9W	R80A PCNA mutant defective in BIR; Saccharomyces cerevisiae; xray 2.65	0			https://doi.org/10.2210/pdb6W9W/pdb
3GPM	Structure of the trimeric form of the E113G PCNA mutant protein; Saccharomyces cerevisiae; xray 3.8	0			https://doi.org/10.1107/S0907444909011329
6QH1	The structure of Schizosaccharomyces pombe PCNA in complex with an Spd1 derived peptide; xray 2.9	279	295	292	https://doi.org/10.1093/nar/gkae011
**prokaryotic B clamps**					
2HIK	apo archael heterotrimeric PCNA; Sulfolobus solfataricus; xray 3.3	0	0	0	https://doi.org/10.1016/j.molcel.2006.08.015

3IFV	Haloferax volcanii archael PCNA; xray 2.0	179	153	174	https://doi.org/10.1186/1472-6807-9-55
1RWZ	Archaeoglobus fulgidus archael PCNA; xray 1.8	315			https://doi.org/10.1016/s0092-8674(03)01036-5
1RXM	Archaeoglobus fulgidus archael PCNA bound to C-terminal region of FEN-1 peptide; xray 2.8	198			https://doi.org/10.1016/s0092-8674(03)01036-5
2NTI	Saccharolobus solfataricus P2 PCNA heterotrimer (archael); xray 2.5	171	0	0	https://doi.org/10.1107/S0907444908021665
3AIX	Sulfurisphaera tokodaii str. 7; xray 2.9; archael; PCNA2-PCNA3 complex from Sulfolobus tokodaii	72	0		https://doi.org/10.1016/j.jsb.2011.02.006
1RXZ	archael; xray 2.0; C-terminal region of A. fulgidus FEN-1 complexed with A. fulgidus PCNA	263			https://doi.org/10.1016/s0092-8674(03)01036-5
7RPX	archael; Archaeal DNA ligase and heterotrimeric PCNA in complex with end-joined DNA; cryo EM 4.2;	359	562	288	https://doi.org/10.1016/j.str.2021.11.002
1GE8	PROLIFERATING CELL NUCLEAR ANTIGEN (PCNA) HOMOLOG FROM PYROCOCCUS FURIOSUS; xray 2.1	281			https://doi.org/10.1110/ps.36401
8PPT	Pyrococcus abyssi DNA polymerase D (PolD) in editing mode bound to P/T substrate containing a mismatch; cryo-EM 2.9	309	0	0	https://doi.org/10.1038/s41467-023-44125-x
3LX1	Crystal Structure analysis of PCNA1 from Thermococcus kodakaraensis tk0535; xray 2.0	102			https://doi.org/10.1073/pnas.1019179108
2IX2	Crystal structure of the heterotrimeric	165	153	101	https://doi.org/10.1107/S1744309106034075

	PCNA from Sulfolobus solfataricus; xray 2.2				
7RPW	Archaeal DNA ligase and heterotrimeric PCNA in complex with adenylated DNA; cryo-EM 4.38	492	612	758	https://doi.org/10.1016/j.str.2021.11.002
5A6D	Proliferating Cell Nuclear Antigen, PCNA, from Thermococcus gammatolerans; xray 2.8	219	244		https://doi.org/10.1016/j.bbrep.2016.08.004
7N5M	PCNA from Thermococcus gammatolerans: crystal III, collection 1, 2.00 A, 1.91 MGy	198	199		https://doi.org/10.1002/prot.26346
6T7Y	Structure of PCNA bound to cPIP motif of DP2 from P. abyssi; xray 2.7	278			https://doi.org/10.1038/s41467-020-15392-9
6T8H	Structure of the DNA-bound PolD-PCNA processive complex from P. abyssi; cryo-EM 3.77	179	174	0	https://doi.org/10.1038/s41467-020-15392-9
4TSZ	bacterial beta-clamp; DNA polymerase sliding clamp from Pseudomonas aeruginosa with ligand; xray 2.0	139	117		https://doi.org/10.1021/jm500467a
6AP4	Crystal structure of the DNA polymerase III subunit beta from Acinetobacter baumannii; 2.95 angstroms	0	0		https://doi.org/10.1016/j.jsb.2018.10.008
7AZD	DNA polymerase sliding clamp from Escherichia coli with peptide 20 bound; xray 2.19	0	0		https://doi.org/10.1021/acs.jmedchem.1c00918
3BEP	E. coli beta clamp bound to DNA; xray 1.92	0	0		https://doi.org/10.1016/j.cell.2007.11.045

7EVP	Cryo-EM structure of the staph aureus Gp168-beta-clamp complex; cryo-em 3.2	0	0	https://doi.org/10.1093/nar/gkab875
8VAL	cryo em Structure of the E. coli clamp loader bound to the beta clamp in a Open-DNAp/t conformation; 3.7 angstroms	0	0	https://doi.org/10.1016/j.jbc.2024.107166
8VAQ	cryo em Structure of the E. coli clamp loader bound to the beta clamp in a Closed-DNA1 conformation; 3.8 angstroms	146	133	https://doi.org/10.1016/j.jbc.2024.107166
4N97	E. coli sliding clamp in complex with 5-nitroindole; xray 1.97	0	0	https://doi.org/10.1021/jm500122r
6FVM	Mutant DNA polymerase sliding clamp from Escherichia coli with bound P7 peptide; xray 1.631	0	0	https://doi.org/10.1021/acsinfecdis.9b00089
6AMS	Crystal structure of the DNA polymerase III subunit beta from Pseudomonas aeruginosa	182	233	https://doi.org/10.1016/j.jsb.2018.10.008
4TR7	Crystal structure of DNA polymerase sliding clamp from Mycobaterium tuberculosis; xray 2.29	208	127	https://doi.org/10.1021/jm500467a
3D1G	Structure of a small molecule inhibitor bound to a DNA sliding clamp; xray 1.64	106	161	https://doi.org/10.1073/pnas.0804754105
6E8E	Crystal structure of the Escherichia coli sliding clamp-MutL complex; xray 2.25	0	0	https://doi.org/10.1093/nar/gkz115
1UNN	Complex of beta-clamp E. coli processivity factor and little finger	0	0	https://doi.org/10.1093/emboj/cdg568

	domain of PolIV; xray 1.9				
8GIY	E. coli clamp loader with closed clamp; cryo-EM 3.7	283	291		https://doi.org/10.1038/s41467-024-52623-9
**AlphaFold3 structures**					
wild-type	PCNA model 1	102.918	0	102.735	
wild-type	PCNA model 2	0	0	0	
wild-type	PCNA model 3	0	0	0	
wild-type	PCNA model 4	0	0	0	
wild-type	PCNA model 5	0	0	0	
Mutant	PCNA(L90I) model 1	0	0	0	
Mutant	PCNA(L90I) model 2	0	0	0	
Mutant	PCNA(L90I) model 3	112.322	106.443	108.049	
Mutant	PCNA(L90I) model 4	111.586	125.478	105.367	
Mutant	PCNA(L90I) model 5	417.289	434.512	306.777	
**The structures below were not included in Figure 6D (main text) because of their unique or unusual hydrophobic site features which we did not characterize in detail (more than one pocket in the same pseudodomain, pockets in adjacent pseudodomains, etc., as described below)**					
6J8Y	Crystal structure of the human RAD9-HUS1-RAD1-RHINO complex, a PCNA-like DNA clamp; xray 2.4	104	409, 156 (on adjacent pseudodomains)	125	https://doi.org/10.1074/jbc.AC119.011816
9EOA	Human PCNA bound to dsDNA; xray, 2.82 angstroms	ND	two adjacent pockets; 250 angstroms closest to L90 and 164 angstroms closest to L101	248 angstroms	https://doi.org/10.1038/s41467-025-59324-x

9B8T	Human polymerase epsilon bound to PCNA and DNA in the nucleotide bound state; cryo-EM 2.95 angstroms	ND	240	two adjacent pockets near 90; 247 and 229 angstroms	https://doi.org/10.1038/s41467-024-52257-x
7TFI	yeast PCNA bound to yeast RFC and DNA; PCNA ring open; saccharomycese cerevisiae; cryo EM 3.41	ND	2 pockets; 114 and 98 angstroms	ND	https://doi.org/10.7554/eLife.77469
8UI8	Cryo-EM map of human clamp-clamp loader ATAD5-RFC-two PCNAs complex in intermediate state 3; cryo-EM 3.10	136, 146, 212 (PCNA dimer w. 3 adjacent internal pockets on one subunit)	0		https://doi.org/10.1038/s41594-024-01332-4
6TNY	Processive human polymerase delta holoenzyme; cryo-EM 3.08	238, 400 (on opposing pseudodomains)	0	0	https://doi.org/10.1038/s41467-020-14898-6
6TNZ	Human polymerase delta-FEN1-PCNA toolbelt; cryo-EM 4.05	0	238, 389 (adjacent pseudodomains)	0	https://doi.org/10.1038/s41467-020-14898-6
9NEA	Human POL epsilon bound to PCNA and DNA with a pre-existing mismatch in the blocked conformation II; cryo-EM 3.81	638,826 (adjacent pseudodomains)	148, 533 (adjacent pseudomains)	111, 322 (adjacent pseudodomains)	https://doi.org/10.1073/pnas.2507232122
9NE9	Human POL epsilon bound to PCNA and DNA with a pre-existing mismatch in the blocked conformation I; cryo-EM 3.88	764, 303 (adjacent pseudodomains)	134, 817 (adjacent pseudodomains)		https://doi.org/10.1073/pnas.2507232122
5E0V	Human PCNA variant (S228I) complexed with FEN1 at xray 2.1 Angstroms	125, 100 (adjacent in same pseudodomain near L90)		150, 223 (adjacent pseudodomains)	https://doi.org/10.1016/j.jmb.2015.11.029
7KC0	Saccharomyces cerevisiae replicative polymerase delta in complex with a primer/template and	120, 87 (adjacent pseudodomains)	0	0	https://doi.org/10.1073/pnas.2017637117

	the PCNA clamp; cryo-EM 3.2				
8PPU	Pyrococcus abyssi DNA polymerase D (PolD) in editing mode bound to P/T substrate containing three consecutive mismatches; cryo-EM 3.02	265	205, 61 (adjacent in same pseudodomain)	0	https://doi.org/10.1038/s41467-023-44125-x
6AMQ	Crystal structure of the DNA polymerase III subunit beta from Enterobacter cloacae; xray 2.67	146, 151 (adjacent pseudomains)	0		https://doi.org/10.1016/j.jsb.2018.10.008
8GIZ	E. coli clamp loader with open clamp; cryo-EM 2. 7	97, 544 (adjacent pseudodomains)	409		https://doi.org/10.1038/s41467-024-52623-9

Supporting Material References

1. Hong, J.Y., J. Cassel, J. Yang, H. Lin, and B.P. Weiser. 2021. High-Throughput Screening Identifies Ascorbyl Palmitate as a SIRT2 Deacetylase and Defatty-Acylase Inhibitor. *ChemMedChem* 16:3484–3494, doi: 10.1002/cmdc.202100343.

2. Kmiecik, D., and J.R. Albani. 2010. Effect of 1-aminoanthracene (1-AMA) binding on the structure of three lipocalin proteins, the dimeric β lactoglobulin, the dimeric odorant binding protein and the monomeric α1-acid glycoprotein. Fluorescence spectra and lifetimes studies. *J Fluoresc* 20:973–983, doi: 10.1007/s10895-010-0643-z.

3. Butts, C.A., J. Xi, G. Brannigan, A.A. Saad, S.P. Venkatachalan, R.A. Pearce, M.L. Klein, R.G. Eckenhoff, and I.J. Dmochowski. 2009. Identification of a fluorescent general anesthetic, 1-aminoanthracene. *Proc. Natl. Acad. Sci. U.S.A.* 106:6501–6506, doi: 10.1073/pnas.0810590106.

4. Woll, K.A., B.P. Weiser, Q. Liang, T. Meng, A. McKinstry-Wu, B. Pinch, W.P. Dailey, W.D. Gao, M. Covarrubias, and R.G. Eckenhoff. 2015. Role for the propofol hydroxyl in anesthetic protein target molecular recognition. *ACS Chem Neurosci* 6:927–935, doi: 10.1021/acschemneuro.5b00078.

5. Bu, W., R. Liu, J.C. Cheung-Lau, I.J. Dmochowski, P.J. Loll, and R.G. Eckenhoff. 2012. Ferritin couples iron and fatty acid metabolism. *FASEB J.* 26:2394–2400, doi: 10.1096/fj.11-198853.

6. Miller, P.S., and A.R. Aricescu. 2014. Crystal structure of a human $GABA_A$ receptor. *Nature* 512:270–275, doi: 10.1038/nature13293.

7. Miled, N., L. Berti-Dupuis, M. Riviere, F. Carrière, and R. Verger. 2003. In vitro lipolysis by human pancreatic lipase is specifically abolished by its inactive forms. *Biochim Biophys Acta* 1645:241–246, doi: 10.1016/s1570-9639(02)00537-x.

8. Roussel, A., N. Miled, L. Berti-Dupuis, M. Rivière, S. Spinelli, P. Berna, V. Gruber, R. Verger, and C. Cambillau. 2002. Crystal Structure of the Open Form of Dog Gastric Lipase in Complex with a Phosphonate Inhibitor*. *Journal of Biological Chemistry* 277:2266–2274, doi: 10.1074/jbc.M109484200.

9. Yuki, K., W. Bu, J. Xi, M. Shimaoka, and R. Eckenhoff. 2013. Propofol shares the binding site with isoflurane and sevoflurane on leukocyte function-associated antigen-1. *Anesth. Analg.* 117:803–811, doi: 10.1213/ANE.0b013e3182a00ae0.

10. Bu, W., L.M. Pereira, R.G. Eckenhoff, and K. Yuki. 2014. Stereoselectivity of Isoflurane in Adhesion Molecule Leukocyte Function-Associated Antigen-1. *PLOS ONE* 9:e96649, doi: 10.1371/journal.pone.0096649.

11. Gu, Y., Q. Liu, P. Chen, C. Guo, Y. Liu, Y. Zhao, Y. Zhang, and D. Lin. 2013. Characterization of the oligomerization and ligand-binding properties of recombinant rat lipocalin 11. *Biochim Biophys Acta* 1834:1–7, doi: 10.1016/j.bbapap.2012.08.018.

12. Paolini, S., F. Tanfani, C. Fini, E. Bertoli, and P. Pelosi. 1999. Porcine odorant-binding protein: structural stability and ligand affinities measured by fourier-transform infrared spectroscopy and fluorescence spectroscopy. *Biochim. Biophys. Acta* 1431:179–188.

13. Johansson, J.S., G.A. Manderson, R. Ramoni, S. Grolli, and R.G. Eckenhoff. 2005. Binding of the volatile general anesthetics halothane and isoflurane to a mammalian beta-barrel protein. *FEBS J.* 272:573–581, doi: 10.1111/j.1742-4658.2004.04500.x.

14. Meillour, P.N.-L., P. Lagant, J.-P. Cornard, F. Brimau, C. Le Danvic, G. Vergoten, and J.-C. Michalski. 2009. Phenylalanine 35 and tyrosine 82 are involved in the uptake and release of ligand by porcine odorant-binding protein. *Biochimica et Biophysica Acta (BBA) - Proteins and Proteomics* 1794:1142–1150, doi: 10.1016/j.bbapap.2009.04.012.

15. Löbel, D., J. Strotmann, M. Jacob, and H. Breer. 2001. Identification of a Third Rat Odorant-binding Protein (OBP3). *Chemical Senses* 26:673–680, doi: 10.1093/chemse/26.6.673.

16. Ramoni, R., F. Vincent, S. Grolli, V. Conti, C. Malosse, F.-D. Boyer, P. Nagnan-Le Meillour, S. Spinelli, C. Cambillau, and M. Tegoni. 2001. The Insect Attractant 1-Octen-3-ol Is the Natural Ligand of Bovine Odorant-binding Protein*. *Journal of Biological Chemistry* 276:7150–7155, doi: 10.1074/jbc.M010368200.

17. Vincent, F., R. Ramoni, S. Spinelli, S. Grolli, M. Tegoni, and C. Cambillau. 2004. Crystal structures of bovine odorant-binding protein in complex with odorant molecules. *European Journal of Biochemistry* 271:3832–3842, doi: 10.1111/j.1432-1033.2004.04315.x.

18. Ramoni, R., S. Bellucci, I. Grycznyski, Z. Grycznyski, S. Grolli, M. Staiano, G.D. Bellis, F. Micciulla, R. Pastore, A. Tiberia, V. Conti, E. Merli, A. Varriale, M. Rossi, and S. D'Auria. 2007. The protein scaffold of the lipocalin odorant-binding protein is suitable for the design of new biosensors for the detection of explosive components. *J. Phys.: Condens. Matter* 19:395012, doi: 10.1088/0953-8984/19/39/395012.

19. Campanacci, V., J. Krieger, S. Bette, J.N. Sturgis, A. Lartigue, C. Cambillau, H. Breer, and M. Tegoni. 2001. Revisiting the specificity of Mamestra brassicae and Antheraea polyphemus pheromone-binding proteins with a fluorescence binding assay. *J Biol Chem* 276:20078–20084, doi: 10.1074/jbc.M100713200.

20. Katre, U.V., S. Mazumder, R.K. Prusti, and S. Mohanty. 2009. Ligand Binding Turns Moth Pheromone-binding Protein into a pH Sensor. *J Biol Chem* 284:32167–32177, doi: 10.1074/jbc.M109.013383.

21. Loebel, D., A. Scaloni, S. Paolini, C. Fini, L. Ferrara, H. Breer, and P. Pelosi. 2000. Cloning, post-translational modifications, heterologous expression and ligand-binding of boar salivary lipocalin. *Biochem J* 350:369–379.

22. Bi, D., J. Yang, J.Y. Hong, P. Parikh, N. Hinds, J. Infanti, H. Lin, and B.P. Weiser. 2020. Substrate-Dependent Modulation of SIRT2 by a Fluorescent Probe, 1-Aminoanthracene. *Biochemistry* 59:3869–3878, doi: 10.1021/acs.biochem.0c00564.

23. Paliwal, A., and P.K. De. 2007. Purification, cloning and regulation of a novel acid-lipase-like protein of hamster expressed in lacrimal glands and tears during lactation. *Biochimica et Biophysica Acta (BBA) - Molecular and Cell Biology of Lipids* 1771:55–65, doi: 10.1016/j.bbalip.2006.10.002.

24. Emerson, D.J., B.P. Weiser, J. Psonis, Z. Liao, O. Taratula, A. Fiamengo, X. Wang, K. Sugasawa, A.B. Smith 3rd, R.G. Eckenhoff, and I.J. Dmochowski. 2013. Direct modulation of microtubule stability contributes to anthracene general anesthesia. *J. Am. Chem. Soc.* 135:5389–5398, doi: 10.1021/ja311171u.

25. Kannan, S., P. Mareeswaran, M. Krishnan, S. Achiraman, N. Spencer, and B.F. Nytingel. 2011. Properties of mouse vomeronasal receptor and assessment of its role in pheromone signalling. *Rapid Communications in Mass Spectrometry* 25:262–270, doi: 10.1002/rcm.4864.

26. Cui, L., K. Kittipongpittaya, D.J. McClements, and E.A. Decker. 2014. Impact of Phosphoethanolamine Reverse Micelles on Lipid Oxidation in Bulk Oils. *Journal of the American Oil Chemists' Society* 91:1931–1937, doi: 10.1007/s11746-014-2544-9.

27. Weiser, B.P., J.T. Stivers, and P.A. Cole. 2017. Investigation of N-Terminal Phospho-Regulation of Uracil DNA Glycosylase Using Protein Semisynthesis. *Biophys. J.* 113:393–401, doi: 10.1016/j.bpj.2017.06.016.

28. Greenwood, S.N., R.G. Belz, and B.P. Weiser. 2022. A Conserved Mechanism for Hormesis in Molecular Systems. *Dose Response* 20:15593258221109335, doi: 10.1177/15593258221109335.

29. Gaubitz, C., X. Liu, J. Magrino, N.P. Stone, J. Landeck, M. Hedglin, and B.A. Kelch. 2020. Structure of the human clamp loader reveals an autoinhibited conformation of a substrate-bound AAA+ switch. *Proc. Natl. Acad. Sci. U.S.A.* 117:23571–23580, doi: 10.1073/pnas.2007437117.
