# Supplementary material for: Biophysical insights into a cryptic ligand site in the hydrophobic core of human PCNA
Source: Biophys J. 2026 May 20;125(13):3471–88. doi: 10.1016/j.bpj.2026.05.027 (PMC13283471; doi:10.1016/j.bpj.2026.05.027)
Supplement: Supplementary file 2 [file mmc4.pdf]

Biophysical Journal
Article

Biophysical Society

Biophysical insights into a cryptic ligand site in the hydrophobic core of human PCNA

Alexis N. Dispensa,[1,2] Sharon N. Greenwood,[1,2] Jason Yang,[1,2] Daniel E. Logatto,[3] James Fusco,[4] E. Railey White,[5] Roderic G. Eckenhoff,[5] Zhiwei Liu,[6] and Brian P. Weiser[1,2,*]

[1]Department of Cell & Molecular Biology, Rowan-Virtua School of Osteopathic Medicine, Rowan University, Stratford, NJ, USA; [2]Department of Cell & Molecular Biology, Rowan-Virtua School of Translational Biomedical Engineering & Sciences, Rowan University, Stratford, NJ, USA; [3]Internal Medicine, Lehigh Valley Health Network, Allentown, PA, USA; [4]Department of Anesthesiology and Perioperative Medicine, Thomas Jefferson University Hospital, Philadelphia, PA, USA; [5]Department of Anesthesiology & Critical Care, University of Pennsylvania Perelman School of Medicine, Philadelphia, PA, USA; and [6]Department of Chemistry & Biochemistry, Rowan University College of Science & Mathematics, Glassboro, NJ, USA

ABSTRACT Proteins contain small pockets that form due to imperfections in residue packing or the rotational and conformational movement of amino acids. In this work, we used the fluorescent probe 1-aminoanthracene (AMA) to detect a small ligand pocket in the hydrophobic core of human proliferating cell nuclear antigen (PCNA), which is a critical protein for DNA replication and repair. Fluorescence measurements of AMA reported that the core of PCNA had a dielectric constant (ε) of 4, which was very apolar and similar to cyclohexane ($\varepsilon = 2$). Protein mutagenesis, photoaffinity labeling, and molecular dynamics simulations localized the binding site for AMA next to PCNA residues L90 and L101, which also interacted with general anesthetics (sevoflurane and propofol). The ligand binding site was cryptic, i.e., it formed transiently and was only detectable in certain structural states of PCNA. Ligand binding to the cryptic site on PCNA structurally stabilized the trimeric protein and reduced its ability to disassemble and reassemble its subunits. Thus, the cryptic site in PCNA's core serves to destabilize the assembled protein and promotes structural and oligomeric flexibility. Finally, the hydrophobic site is widely conserved among homologous β clamp proteins with a similar fold as PCNA. This work highlights how small fluorescent probes can reveal ligand sites within proteins, defines the chemical features of protein hydrophobic cores, and introduces a novel approach to modulate the oligomeric stability of PCNA.

SIGNIFICANCE Cryptic binding sites for small molecules appear and disappear on proteins as they sample different structural states. Many of these sites can be targeted for therapeutic purposes but are not well understood. In addition to surface binding sites, ligand pockets also form internally where hydrophobic residues are not tightly packed together. We used a fluorescent probe to detect these hydrophobic pockets on proteins. After a small screen, we identified a ligand binding site in the hydrophobic core of a human protein called PCNA. Clinically used general anesthetics also interacted with the site. We characterized physical, chemical, and functional features of the new ligand site on PCNA, which influenced the stability of the trimeric protein.

INTRODUCTION

We have long understood that the interior of soluble proteins consists of hydrophobic residues that pack together to avoid the aqueous environment and help stabilize protein structure. However, due to imperfections in the packing of residue side chains, it was reported that on average ~44% of the protein core is actually unoccupied volume, which can contribute to the formation of various cavities or tunnels inside proteins.[1] These spaces affect protein dynamics and conformational flexibility and can play critical roles in allosteric regulation or ligand migration.[2–4] These spaces may be transient or "cryptic," appearing only in certain structural states due to protein dynamics, including small or large atomic movements such as side chain rotations or conformational changes.[5–8] Cryptic and hydrophobic sites can be targeted with small molecules for therapeutic purposes, but there is much to understand about the formation of cryptic cavities, their persistence, and their physicochemical features.[5,6,9]

Submitted November 20, 2025, and accepted for publication May 15, 2026.

*Correspondence: weiser@rowan.edu

Editor: Gregory Bowman.

https://doi.org/10.1016/j.bpj.2026.05.027

© 2026 The Author(s). Published by Elsevier Inc. on behalf of Biophysical Society.
This is an open access article under the CC BY license (http://creativecommons.org/licenses/by/4.0/).

Techniques such as X-ray crystallography, NMR, and molecular dynamics (MD) simulations have allowed us to directly observe cavities in the protein core and calculate parameters such as their size, geometry, and polarizability.[3,6,7,10–13] Detecting these spaces with molecular probes is challenging because of their small sizes, structural plasticity, and hydrophobicity, which limit well-defined molecular contacts in the sites and reduce opportunities for strong ligand-protein interactions such as hydrogen bonding. However, we note that an entire class of lipophilic drugs—injectable and inhalable general anesthetics—bind weakly to hydrophobic protein sites (μM to mM K_d values), yet anesthetics can be surprisingly selective for their protein targets.[14–16] General anesthetics are small (<250 Da) and lipophilic compared with conventional therapeutics, but even these molecules are larger than the minimal chemical probes, such as benzene or xenon (itself an anesthetic), which are used to explore smaller internal protein cavities.[3,10,17–19]

In this work, we used fluorescence to probe hydrophobic protein sites using 1-aminoanthracene (AMA), which has fluorescence that "turns on" when the molecule is in apolar environments.[20–23] Low molecular weight fluorophores such as AMA can produce various changes in quantum yield and solvatochromism in response to their molecular surroundings, solvent polarity, or pH.[21,24–26] AMA also has general anesthetic properties[16,22] and has been reported to bind over a dozen structurally diverse proteins (Table S1). Known AMA binding sites on proteins frequently overlap with regions targeted by other anesthetics, lipids, and small lipophilic molecules such as pheromones and odorants (Table S1). Several AMA binding sites are known to be druggable and correspond to pockets or cavities bound by clinically used compounds.[16,22,27–30]

We used AMA to discover a novel cryptic site in the hydrophobic core of human proliferating cell nuclear antigen (PCNA), which is a ring-shaped protein composed of three identical subunits. In the cell, PCNA encircles dsDNA and acts as a scaffold for localizing eukaryotic replication and repair proteins to the replication fork.[31] PCNA is part of a large, evolutionarily conserved family of sliding clamps that are structurally and functionally similar, including prokaryotic β clamps, which enable processivity for proteins on DNA. PCNA is also a drug target for cancers because of its critical importance for cellular DNA replication.[32,33] We characterized structural and chemical features of the cryptic site on PCNA and identified other ligands that bind to the site (e.g., general anesthetics). The cryptic site exists as empty space inside PCNA's core that weakly destabilizes the trimeric protein and promotes its disassembly and reassembly, which spontaneously unloads PCNA from dsDNA.[34,35] Our work supports the idea that open cavities or space within the protein core inherently affect structural stability and can influence processes such as oligomerization or exchange in multi-subunit assemblies.

MATERIALS AND METHODS

Chemicals

Chemical and pharmacologic properties of relevant small molecules can be found in Figure 1. AMA was purchased from TCI America (Portland, Oregon), and propofol (2,6-diisopropylphenol) was purchased from Alfa Aesar (Ward Hill, Massachusetts). The synthesis of *meta*-azi-propofol (AziP*m*) has previously been reported.[36] Sevoflurane (fluoromethyl-1,1,1,3,3,3-hexafluoroisopropyl ether) was purchased from TCI America.

Proteins

Proteins that were purchased included human apolipoprotein E (ApoE) (amino acids 19–317 with a C-terminal 6xHis tag) (Bon Opus Biosciences, Shawnee, Kansas); pyruvate kinase from rabbit muscle (Roche, Basel, Switzerland); lactate dehydrogenase from rabbit muscle (Roche); *E. coli* single-stranded binding protein (Qiagen, Hilden, Germany); thermolysin (Promega, Madison, Wisconsin); streptavidin (Promega); and SpyCatcher3 (Bio-Rad, Hercules, California). Human ΔN555-Replication factor C (RFC), which was composed of five subunits, was a generous gift from Dr. Brian Kelch (University of Massachusetts).[39] 6xHis-PCNA was expressed and purified as described previously[40]; when relevant, standard QuikChange/site-directed mutagenesis was used to introduce mutations onto the plasmid encoding the protein. Expression and purification of full-length human uracil DNA glycosylase (UNG2) and *S. cerevisiae* ubiquitin-like protease 1 catalytic domain (amino acids 403–621) have been reported.[40] Human sirtuin isoform 2 (SIRT2) catalytic domain (amino acids 34–356) and human sirtuin isoform 3 (amino acids 120–399 with an N-terminal 6xHis tag) were also expressed and purified as previously described.[27,41]

Purified replication protein A-70N contained amino acids 1–120 of the human RPA1 gene with an N-terminal 8xHis tag. We originally inserted the 8xHis tag into the p11d-tRPA plasmid that encodes RPA1 along with other RPA subunits.[42] Then, we amplified the 8xHis tag and residues 1–120 flanked by NdeI and NotI sites for standard restriction enzyme cloning into a pET21a vector.[40] The tagged replication protein A-70N construct was expressed and purified with standard procedures for Ni^{2+} chromatography followed by size exclusion chromatography. The sequences of all plasmids used for protein expression were verified with Sanger sequencing.

Human FAM72a was a tag-free recombinant protein that was produced in the following manner. The human FAM72a gene was obtained from a plasmid that was a generous gift from Dr. Jessica Stewart (University of North Carolina). This gene was amplified and inserted into a pET21a vector using standard cloning methods such that the following

Ligand	Mass (g/mol)	van der Waals Volume (Å^3)	Octanol/Water Partition Coefficient (LogP)	Polar Surface Area (Å^2)	Tadpole Anesthesia EC_{50} (μM)	Type of Anesthetic
1-Aminoanthracene 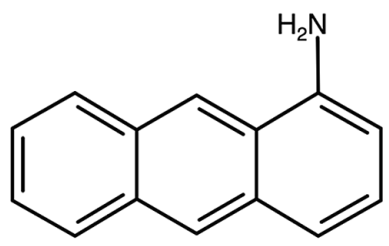	193	179	3.7	26	16	Fluorescent analog
Propofol 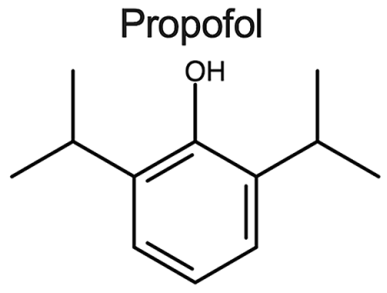	178	192	3.8	20	3	Clinical injectable
meta-Azi-Propofol 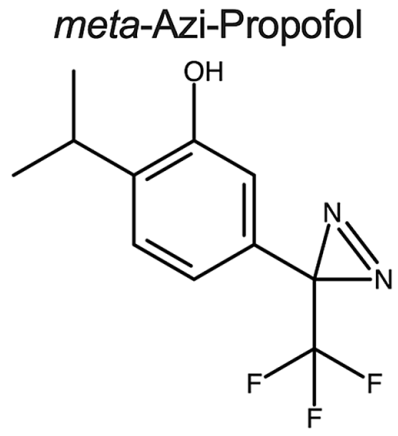	244	200	3.6	26	3	Photoactive propofol analog
Sevoflurane 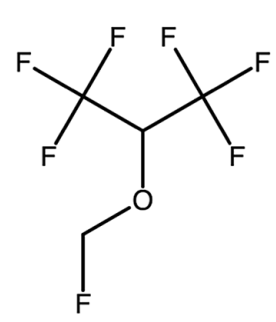	200	154	2.8	9.2	192	Clinical inhalable

Figure 1. Chemical structures and properties of anesthetic compounds used in this study. Values in the table were previously reported.[16,22,36–38]

construct was encoded: 8xHis-SUMO-FAM72a with a Gly-Ser-Gly-Ser-Gly linker between the 8xHis tag and SUMO domain.[40] The protein was expressed and purified with standard procedures, which included removal of the 8xHis-SUMO tag with the SUMO protease during purification.[40]

Untagged human PCNA and SpyTag-His-PCNA were expressed from the same pET21a vector as 6xHis-PCNA after truncating or enlarging the gene with site-directed mutagenesis.[40] Untagged PCNA was expressed as the wild-type protein sequence (UniProt: P12004). SpyTag-His-PCNA had the SpyTag-containing sequence MAHIVMVDAYK PTKGG upstream of an N-terminal 6xHis tag. After standard protein expression conditions,[40] untagged PCNA and SpyTag-His-PCNA were purified in the following manner. Cell pellets were resuspended in lysis buffer (20 mM Tris-HCl, pH 7.5, 30 mM NaCl, 1 mM DTT, protease inhibitor cocktail) and lysed with a microfluidizer. After centrifugation to remove cell debris, the lysate was flowed through SP-sepharose resin (Cytiva, Marlborough, Massachusetts), and then PCNA in the flow through was captured on Q-sepharose resin (Cytiva). Protein was eluted with a stepwise gradient of 100–650 mM NaCl dissolved in lysis buffer. PCNA-containing fractions were pooled and diluted 1:5 in Buffer A (20 mM Tris-HCl, pH 7.5, 30 mM NaCl, 1 mM DTT, 5% glycerol). The protein was further purified by fast protein liquid chromatography by loading the material onto a Bio-Rad ENrich Q-column and eluting with a linear gradient of Buffer B (20 mM Tris-HCl, pH 7.5, 1 M NaCl, 1 mM DTT, 5% glycerol).

Fluorescence assays with protein and AMA

All assays were performed at 23°C in PBS with 1 mM DTT as described elsewhere.[27] A 200-μL quartz microcuvette (3 mm path length) was used with a Horiba Fluoromax-4 fluorometer for all measurements. To measure background spectra, protein samples were diluted in PBS and excited with 410 nm while an emission scan was collected from 420 to 700 nm. AMA was then added to achieve a final concentration of 100 nM, and the sample was gently mixed by pipetting before repeating the fluorescence scan. Background spectra from protein alone were subtracted from the spectra of protein with AMA, and the resulting background-subtracted counts per second were plotted against emission wavelength.

To determine binding affinities between AMA and proteins, we measured the fluorescence of 100 nM AMA in the presence of increasing protein concentrations while

subtracting protein-only background fluorescence as described above. The peak fluorescence value at each protein concentration was extracted from the AMA spectra, and then, this value was normalized by dividing it by the maximum fluorescence observed in each assay. Normalized fluorescence was plotted as Y values against the protein concentration as X values. A curve was fit to the data to determine the K_d for AMA interacting with the protein using the quadratic binding equation:

$$Y = Y_{min} - \left[\frac{Y_{min} - Y_{max}}{2[AMA]}\right] \times \left[b - \sqrt{b^2 - 4\,[PROTEIN][AMA]}\right]$$

$$b = K_d + [PROTEIN] + [AMA]$$

Here, Y_{min} and Y_{max} were the minimum and maximum normalized intensities, [AMA] was the total AMA concentration, [PROTEIN] was the protein concentration, and K_d was the protein concentration at which half-maximal saturation occurred.

For AMA/ligand competition assays, 10 μM PCNA was equilibrated with the indicated concentration of competitor ligand (along with 0.5% DMSO as the vehicle), and a background emission scan was collected. AMA was then added to a final concentration of 100 nM, the sample was mixed by pipetting, and the fluorescence scan was repeated. Background spectra of PCNA with competitor ligand were subtracted from spectra with AMA as before. The fluorescence of AMA alone in the absence of protein was subtracted from all values because this was the theoretical minimum fluorescence that could occur in the competition assay. The fluorescence intensities were then normalized by dividing the values by the fluorescence of 10 μM PCNA with 100 nM AMA in the absence of competitor and multiplying by 100%. Normalized fluorescence intensities were plotted as Y values against the $\log_{10}$ of the competitor ligand concentration as X values, and the data were fit with a sigmoidal curve:

$$Y = Y_{min} + \frac{Y_{max} - Y_{min}}{1 + 10^{n*(log(IC_{50}) - X)}},$$

where Y_{min} and Y_{max} were set to 0 and 100, respectively, the IC_{50} was the X value at the halfway point between the two asymptotes, and n was the Hill slope. Under our assay conditions, the IC_{50} values for the displacement of AMA from PCNA proteins using sevoflurane/propofol were essentially equal to the K_d values for the interaction of sevoflurane/propofol with PCNA.[43] Fractional occupancies of PCNA bound to sevoflurane in specific assays were estimated using the equation

$$\%Occupancy = \frac{[L]}{K_d + [L]} \times 100,$$

where [L] was the concentration of sevoflurane, and K_d was the affinity of PCNA for sevoflurane.

To evaluate the relationship between residue hydrophobicity in the cryptic site and the fluorescence of AMA, we considered how the cryptic site contained leucine residues at positions 90 and 101 in the wild-type protein. All of the PCNA variants that we tested retained one leucine at 90 or 101 along with a second amino acid for the mutation; therefore, we examined the effect of the second (nonleucine) residue that was at position 90 or 101. Hydrophobicity scores for that second residue were obtained from Monera et al. who derived a hydrophobic scaling system that was also used by Fpocket.[44] The hydrophobicity scores for the second residues were plotted as X values against the peak emission wavelengths of AMA in the presence of the corresponding variants as Y values. A curve was fit to the data using the equation

$$Y = Y_{min} + (Y_0 - Y_{min}) \times \exp(-K * X),$$

where Y_{min} was the minimum Y value at the plateau, Y_0 was the initial Y value at the lowest X values, and K was a constant controlling the transition from Y_0 to Y_{min}.

Fluorescence measurements with AMA in solvents and calculation of protein dielectric constants

Background spectra of solvents were collected, and then, AMA (200 nM) was dissolved in the solvents and scanned with our fluorometer (excitation = 410 nm, emission = 420–700 nm). The background spectra were subtracted from the spectra of solvent with AMA. The peak emission wavelength of AMA in each solvent was then extracted from the background-subtracted spectra after smoothing the spectra using a second-order Savitzky-Golay polynomial filter (nine-point window) as implemented in GraphPad Prism v10.[45] The peak emission wavelengths were plotted as Y values against the solvent dielectric constants (ε) as X values. A hyperbolic curve was fit to the data using

$$Y = Y_{min} + \frac{(Y_{max} - Y_{min}) * X}{K + X},$$

where Y_{min} and Y_{max} were the minimum and maximum emission wavelengths (nm) of AMA, respectively, and K was the solvent polarity at which AMA's emission wavelength was halfway between its minimum and maximum. Subsequently, we used this as a standard curve to determine the dielectric environment of AMA binding sites by interpolation using the peak emission wavelength of AMA in the presence of individual proteins or DOPC; we also used the Savitzky-Golay smoothing method in determining peak emission wavelengths for these spectra.

PCNA photolabeling and mass spectrometry

Purified 6xHis-PCNA was diluted in PBS with 1 mM DTT, pH 7.4, to a final concentration of 0.47 mg/mL (30 μM PCNA monomer). AziP*m* was added to a final concentration of 10, 20, or 50 μM on ice for 25 min in the dark; every sample also contained 1% DMSO from preparing AziP*m* stock solutions. Samples were transferred to a 1-mm pathlength quartz cuvette and exposed to a 350-nm light source (Rayonet RPR-3500 lamp, Branford, Connecticut) for 25 min at a distance of <1 cm. For a control "sham" photolabeling experiment, 6xHis-PCNA was incubated with 1% DMSO and exposed to the same 350-nm light source without the addition of AziP*m*. After photolabeling, the protein was precipitated with acetone, resuspended in buffer containing DTT, and alkylated with iodoacetamide. 6xHis-PCNA was then digested in solution with trypsin, desalted with C18 StageTips,[46] and then analyzed with an Orbitrap Elite Hybrid Ion Trap-Orbitrap Mass Spectrometer (MS) coupled to an Easy-nanoLC 1000 system. Spectra of PCNA peptides were analyzed with MaxQuant version 2.1.4.0.[47] The analysis included the dynamic oxidation of methionine (+15.9949 m/z) and static alkylation of cysteine by iodoacetamide (+57.0215 m/z). PCNA amino acids were also searched for a possible mass adduct of +216.0762 m/z, which corresponded to a residue photolabeled with AziP*m*.[48,49] Up to two missed trypsin cleavages were allowed in the searches. The spectra showing an AziP*m* adduct on PCNA peptides were then manually reviewed for confirmation. Using the human PCNA sequence from the UniProt database (UniProt: P12004), the following sequence coverages were obtained for the photolabeling conditions: 10 μM AziP*m*, 83.1%; 20 μM AziP*m*, 83.1%; 50 μM AziP*m*, 83.1%; DMSO only, 95.8%. Further details on the photolabeling and mass spectrometry methods can be found elsewhere.[50]

MD simulations

1-μs MD simulations of wild-type PCNA and PCNA(L90I) were run using an available crystal structure as the starting structure (PDB: 1VYM).[51] Independent 500-ns simulations of PCNA and PCNA(L90I) were run using an AlphaFold model as the starting structure, downloaded from AlphaFold Protein Structure Database.[52] MD simulations were performed using the Amber suite of biomolecular simulation programs (version 22)[53] with the ff19SB force field for protein[54] and protonation states assigned according to pH 7.0. The system was solvated in a cubic box of OPC water molecules with periodic boundary conditions,[55] ensuring a minimum distance of 10 Å from the protein edge to the box boundary. To mimic physiological ionic strength, K^+ and Cl^- ions were added to neutralize the system and achieve a final salt concentration of 150 mM. The system was equilibrated following a standard protocol: (1) relaxing (by energy minimization) solvent and ions with protein constrained, (2) relaxing the entire system to remove bad contacts in initial structures, (3) heating to 300 K gradually, and (4) equilibration under constant pressure (NPT) at 1 atm for 1 ns to ensure density matching that of an aqueous environment. Production simulations were run in the NVT ensemble. Long-range electrostatics were treated using the particle mesh Ewald method. MD trajectories were saved every 10 ps for downstream analysis. All visualizations were generated with PyMol (v3.1.5.1) or VMD.[56]

Detection and analysis of cryptic binding sites

Putative ligand-binding pockets on human PCNA were detected using the Fpocket suite (version 4.0) using default parameters.[57] The pocket characteristics of PCNA and PCNA(L90I) during their MD trajectories were analyzed using MDpocket.[58] Specifically, the structure of PCNA at every 10 ns during the simulation was aligned to the starting (crystallographic or AlphaFold) structure based on root mean square fit of protein backbone atoms. Then, grid-based pocket detection identified all cavities along the trajectory. The density grid (.dx file) was loaded into VMD with the starting structure, and the iso-value was set to the lowest level that produced a pocket within 5 Å of residues 90 and 101 (i.e., we localized the analysis to the pocket at residues 90 and 101) (actual iso-values ranged from 0.1 to 0.5, depending on the simulation). The alpha spheres composing the site were selected, and MDpocket was re-run on the simulation using this pocket definition to calculate features of our site of interest, such as pocket volume. Frames containing pockets that overlap with alpha spheres from our selected sites, and which have specific features such as volumes large enough to accommodate our ligands, could then be identified and extracted as .pdb files. Detailed maps (.pdb files and .dx files) showing the shapes of the ligand site can be found in the Zenodo data sets that accompany this work.

Pockets on ∼100 structures of human and yeast PCNA, as well as various prokaryotic β clamps, were also detected using Fpocket with default parameters.[57] The protein structures with the output pockets were aligned to human PCNA (PDB: 1VYM) to identify internal hydrophobic sites on the surveyed proteins that were positioned analogously to human L90/L101. AlphaFold3 models of human PCNA and PCNA(L90I) were generated using AlphaFold Server, which defaults to provide five models per run, all of which were analyzed.[59]

Ligand docking for MD simulations

Ligand structures were prepared with LigPrep to generate low-energy 3D conformers with proper protonation states at physiological pH (7.0 ± 0.5) using Epik.[60,61] All possible tautomers and stereoisomers were generated, and energy minimization was performed using the OPLS4 force field.[62]

Protein structures for docking were extracted from the MD simulations where the cryptic sites were open. Receptor grids were generated using Glide[63–65] centered on the putative binding site, with the grid box size adjusted to fully encompass the pockets identified by MDpocket. Standard Precision (SP) Glide docking was performed with flexible ligand sampling, and the top-ranked poses were analyzed based on GlideScore and predicted binding modes. Key interactions were visualized and evaluated in Maestro (Schrödinger Release 2024-4), and the best-scoring poses were used as starting structures for subsequent MD simulations.

PCNA thermostability measurements with fluorescence

PCNA unfolding was assessed by measuring intrinsic tryptophan fluorescence as a function of temperature in the presence and absence of sevoflurane.[66] Experiments were conducted in PBS with PCNA at a final concentration of 3 μM. Sevoflurane was added from a freshly prepared stock in DMSO to a final concentration of 2.5 mM, and the same volume of DMSO (1%) was added to control samples. Samples (3.8 mL) were loaded into a synthetic quartz cuvette, sealed tightly and then subjected to a controlled temperature ramp from 25°C to 70°C with 3-min holds every 2.5°C increment using a Horiba Fluoromax-3 instrument equipped with a Peltier temperature controller. Tryptophan fluorescence was monitored with excitation at 280 nm and emission at 325 nm. For every experiment, fluorescence values were normalized such that the initial fluorescence of the folded protein at 25°C was set to 100, and the final fluorescence at 70°C was set to 0. The unfolding transition midpoint (T_m) was determined by plotting the normalized fluorescence intensities as Y values against the temperature as X values and fitting the data with a sigmoidal equation:

$$Y = Y_{min} + \frac{Y_{max} - Y_{min}}{1 + \left(\frac{T_m}{X}\right)^n},$$

where Y_{min} and Y_{max} were the normalized fluorescence intensities at the lower and upper asymptotes, T_m was the X value at the halfway point between the two asymptotes, and n was the Hill slope.

PCNA subunit exchange

Subunit exchange between PCNA trimers in the absence and presence of sevoflurane was measured as follows. Reactions (600 μL) were initiated by mixing equimolar concentrations (10 μM each) of untagged PCNA and SpyT-His-PCNA in Buffer C (10 mM Tris-HCl, pH 7.5, 100 mM NaCl, 1 mM TCEP). At defined time points (0.33, 15, 30, 60, 90, 120 min), 100-μL aliquots were withdrawn, applied to pre-equilibrated Ni^{2+}-NTA resin in a microcentrifuge tube, and gently mixed by pipetting to allow SpyT-His-PCNA to bind the resin. These were then centrifuged at 15,000 RPM for 1 min, washed with Buffer C, and then, SpyT-His-PCNA-containing trimers were eluted with Buffer D (10 mM Tris-HCl, pH 7.5, 100 mM NaCl, 1 mM TCEP, 300 mM imidazole). SpyCatcher3 (20 μM) (referred to as simply "SpyCatcher" in this work) was added to the eluate for 3 h to allow for its covalent reaction to the SpyTag of SpyT-His-PCNA. Samples were then separated by SDS-PAGE to resolve untagged PCNA from the SpyCatcher-SpyT-His-PCNA complex. After staining with Coomassie, band intensities for untagged PCNA and SpyCatcher-SpyT-His-PCNA were quantified using FIJI/ImageJ.[67]

To have the band intensities of untagged PCNA and SpyCatcher-SpyT-His-PCNA reflect their relative molar amounts, we had to correct for the darker staining of the latter protein per mol because of its additional mass. We multiplied the intensity of untagged PCNA by a correction factor (C_f). The C_f was determined by analyzing known, molar equivalent amounts of untagged PCNA and SpyCatcher-SpyT-His-PCNA (i.e., "tagged PCNA") on a Coomassie-stained SDS-PAGE gel and using the ratio

$$C_f = \frac{Intensity_{tagged\ PCNA}}{Intensity_{untagged\ PCNA}},$$

where intensity values came from FIJI/ImageJ quantification. Thus, the relative molar amount of untagged/tagged PCNA subunits in each lane could be determined. The molar ratio of untagged/tagged PCNA subunits was plotted on the Y axis against time on the X axis, and the single-phase exponential data sets were fit with a curve using

$$Y = (Y_{min} - Y_{max}) * exp(-k_{obs} * X) + Y_{max},$$

where Y_{min} was molar ratio at time 0 (which was constrained to 0), Y_{max} was molar ratio at the plateau at longer time points, and k_{obs} was a rate constant controlling the transition from Y_{min} to Y_{max}.

If all of the untagged and tagged PCNA subunits distributed completely, evenly, and unhindered during the course of the assay, then the expected molar ratio of each trimeric species before pull-down with the Ni^{2+} resin would be as follows: trimers composed of three untagged subunits would represent 1/8 of the total population; those with two untagged and one tagged subunit, 3/8; those with one untagged and two tagged subunits, 3/8; and trimers with three tagged subunits, 1/8. Only the trimer composed entirely of untagged subunits will fail to bind to the Ni^{2+} resin, while the remaining species are retained. Consider the total number of subunits within the retained population along with the fraction of each species. One trimer containing three tagged subunits contributes 3 tagged subunits. Three trimers composed of one untagged and two tagged subunits contribute a total of 3 untagged and 6 tagged subunits. Three

trimers composed of 2 untagged and 1 tagged subunit contribute a total of 6 untagged and 3 tagged subunits. The ideal untagged/tagged subunit ratio at equilibrium is therefore 9/12 or 3/4.

RESULTS

Fluorescence measurements of AMA in various protein and chemical environments

To explore biophysical, chemical, and pharmacologic features of hydrophobic protein sites, we aimed to identify new interaction partners of AMA and to characterize its binding sites by measuring how the ligand's fluorescence was affected by various proteins and chemical environments. In PBS buffer alone (pH 7.4), 100 nM AMA exhibited a peak emission wavelength of 560 nm with very weak fluorescence intensity (Figure 2A). The amine of AMA is neutral in PBS with a large dipole in the excited state, which promotes hydrogen bond interactions with the aqueous solvent and dielectric stabilization of the excited state, which contribute to nonradiative decay (i.e., loss of energy without light emission).[21,24,69–71] However, in the presence of certain proteins including 10 μM PCNA, ApoE, or SIRT2, the fluorescence intensity of AMA increased up to 17-fold, and its peak emission wavelength significantly blueshifted, which was indicative of AMA binding to hydrophobic regions on the proteins (Figures 2A; Table 1).[22,23] In the protein environment, the fluorophore was shielded from aqueous PBS, reducing polar solvent interactions that dampen AMA fluorescence.[21,24] In contrast, the fluorescence intensity of AMA was unchanged in the presence of other proteins (e.g., UNG2), which indicated a lack of AMA interaction with hydrophobic sites on those macromolecules (Figures 2A; Table 1). The ability of proteins to induce changes in AMA fluorescence was not related to protein size, indicating that there was some binding selectivity for AMA beyond bulk nonspecific interactions. We further determined a K_d of 84 μM for AMA's interaction with PCNA and a K_d of 15 μM for its interaction with ApoE (Figures 2B and 2C). We previously characterized the molecular interaction between AMA and SIRT2, which occurred with a K_d value of 37 μM.[27]

We were intrigued by the different emission wavelengths that AMA exhibited when bound to different proteins considering the experimental conditions were otherwise

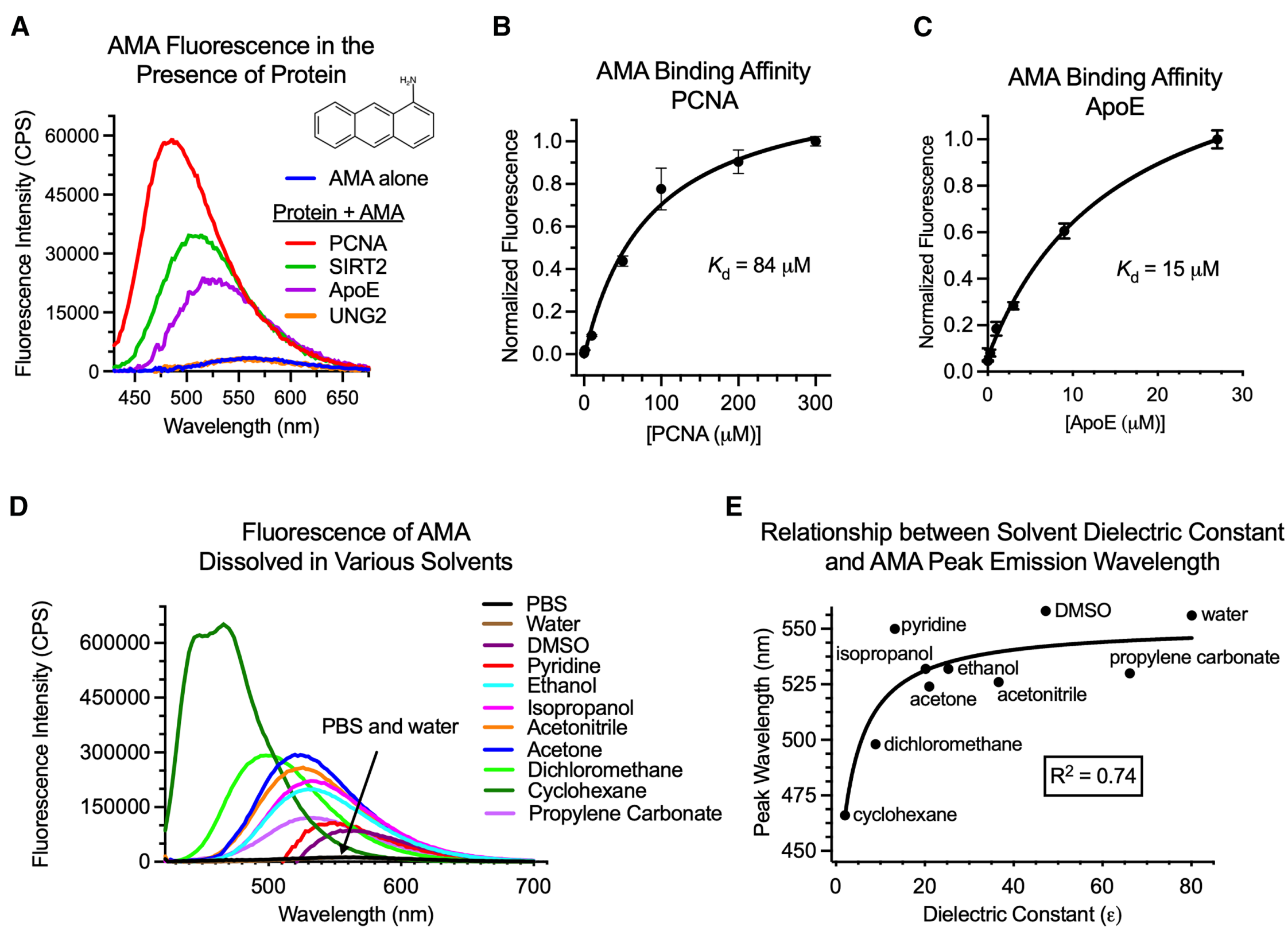

Figure 2. Fluorescence shifts of AMA when bound to proteins and in various solvents. (A) Fluorescence emission spectra of 100 nM AMA in PBS by itself or with 10 μM of the indicated proteins. (B) Binding isotherm showing the interaction of AMA with recombinant PCNA. Data points represent mean ± standard error. (C) Binding isotherm showing the interaction of AMA with recombinant ApoE. Data points represent mean ± standard error. (D) Fluorescence emission spectra of 200 nM AMA dissolved in the indicated solvents. (E) Nonlinear relationship between solvent ε and the peak emission wavelength of AMA from the data shown in (D). ε values were published elsewhere.[68]

Table 1. Fluorescence changes of 100 nM AMA in the presence of 10 μM protein

Protein	Molecular weight (kDa)[a]	Fold change in AMA fluorescence intensity	AMA peak emission wavelength (nm)	Site dielectric constant (ε)[b]
Proliferating cell nuclear antigen (PCNA) (*H. sapiens*)	29	17	486	4
Sirtuin isoform 2 (SIRT2) (*H. sapiens*)	37	10	506	7
Apolipoprotein E (ApoE) (*H. sapiens*)	34	7	526	15
Pyruvate kinase (*O. cuniculus*)	57	3	530	19
ΔN555-Replication factor C (RFC) (*H. sapiens*)	217	3	532	21
Sirtuin isoform 3 (*H. sapiens*)	31	2	532	N/D[c]
Lactate dehydrogenase (*O. cuniculus*)	37	2	538	N/D[c]
Single-stranded binding protein (*E. coli*)	19	2	560	N/D[c]
Thermolysin (*G. stearothermophilus*)	36	1	580	N/D[c]
Replication protein A-70N (*H. sapiens*)	13	1	560	N/D[c]
Uracil DNA glycosylase isoform 2 (UNG2) (*H. sapiens*)	35	1	550	N/D[c]
Ubiquitin-like protease 1 (*S. cerevisiae*)	25	1	552	N/D[c]
FAM72a (*H. sapiens*)	17	1	532	N/D[c]
Streptavidin (*S. avidinii*)	17	1	532	N/D[c]

[a]Molecular mass of the protein polypeptide that was expressed and/or purified. Purification tags such as poly-histidine were excluded from the calculation.
[b]Determined by interpolation from the standard curve in Figure 2E.
[c]N/D, not determined. The signal above background was weak and noisy.

the same. We reasoned that variations in the dielectric microenvironment of the binding sites affected AMA's emission. To demonstrate this, we measured the fluorescence of AMA dissolved in solvents covering a wide range of polarities in the absence of protein (Figure 2D). We observed a strong nonlinear correlation between the dielectric constant (ε) of the solvent and the peak emission wavelength of AMA (Figure 2E). The hyperbolic relationship has been seen for other solvatochromic fluorophores and reflects a nonlinear dependence of excited-state stabilization on solvent dielectric constant[72,73]; polarity effects increase steeply at low ε but tend to saturate at high ε due to limits in solvent reorientation around the excited fluorophore and specific solute-solvent interactions. The spectroscopic absorbance of AMA did not change as dramatically in the various solvents (Figure S1).

We determined ε values for the AMA binding sites on our proteins of interest by interpolation using the data in Figure 2E as a standard curve. The AMA site on PCNA had an ε of 4, which was very apolar and indicated a chemical environment within the protein that was similar to cyclohexane (Table 1). The AMA binding sites on SIRT2 and ApoE were more polar and had dielectric constants that were in line with dichloromethane or pyridine (Figures 2E; Table 1). As an additional point of reference for hydrophobicity, and to broaden the utility of AMA as a dielectric constant probe, we generated phospholipid bilayers in PBS using DOPC and measured the fluorescence of AMA after it partitioned into the membrane.[22] AMA reported a peak emission wavelength of 536 nm and an ε value of 27 at its depth inside the membrane (Figure S2). In conclusion, we identified several new protein binding partners of AMA and found that the ligand reported a broad range of dielectric environments inside protein hydrophobic sites.

Discovery of a highly apolar ligand site on PCNA

We began our search for AMA binding sites on several proteins that were not previously known to us as binding partners for AMA or chemically similar molecules including general anesthetics. We were unable to competitively displace AMA from its binding site on ApoE using propofol or sevoflurane (Figure S3). ApoE binds various lipids and fatty acyl chains that were incompatible with AMA competition assays because of their tendency to form bilayers or micelles that attract the fluorophore (Figure S2).[74] We also found that sevoflurane did not displace AMA from pyruvate kinase; the inability of sevoflurane to displace AMA from ApoE or pyruvate kinase may indicate nonsaturable or nonconserved ligand interactions. In contrast, AMA was competitively displaced from PCNA by propofol and sevoflurane (Figures 3A–3D), with displacement from a shared binding site reducing AMA's fluorescence as it returned to the aqueous environment. Propofol displaced AMA from PCNA with an IC_{50} of 259 μM, and sevoflurane displaced AMA with an IC_{50} of 2.2 mM (Figures 3B and 3D). These results suggested a specific binding site on PCNA that could be saturated with ligand. Coupled with the compelling hydrophobicity of the ligand site (Table 1) and the central role of PCNA in cell physiology, we selected it for further study.

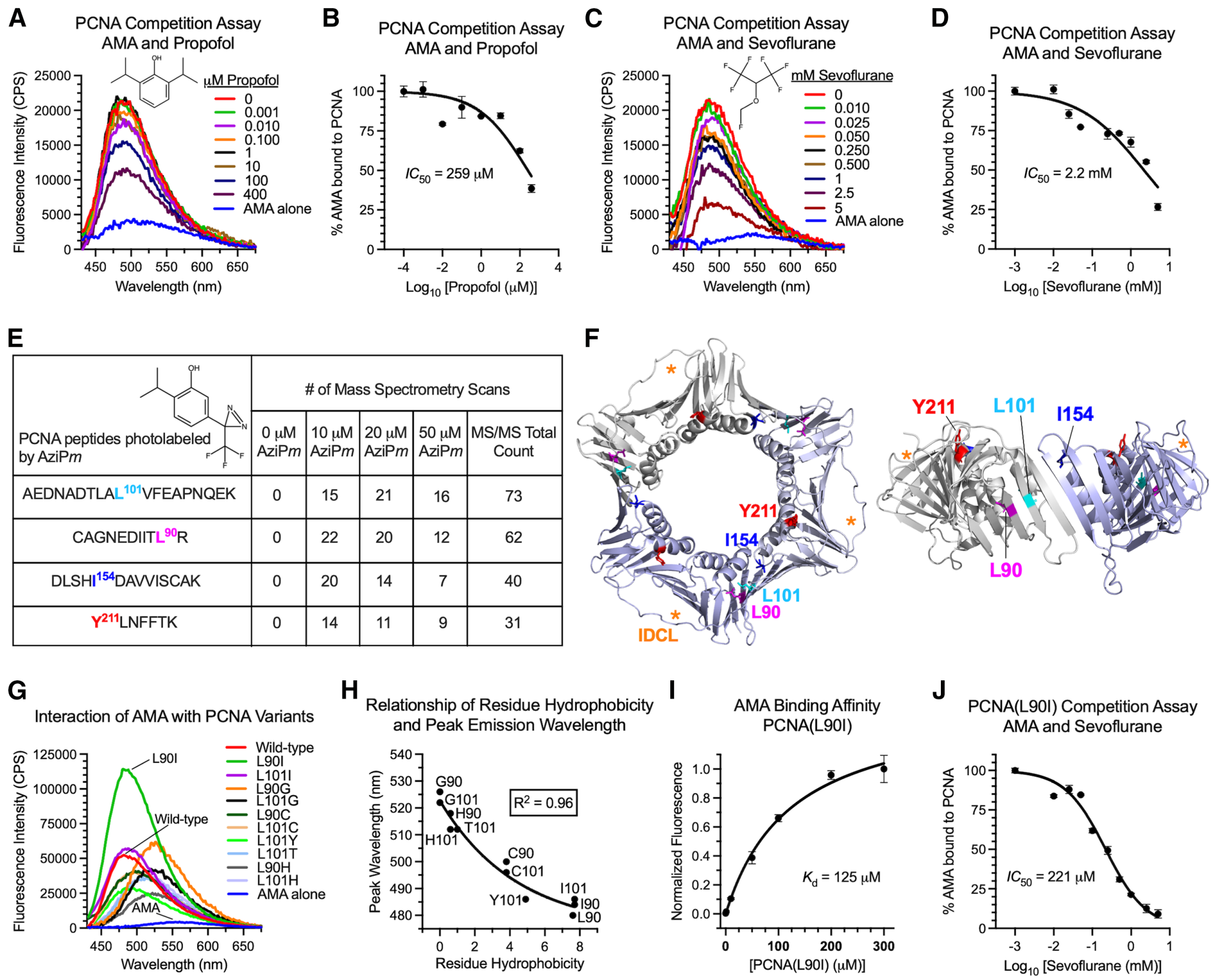

PCNA peptides photolabeled by AziP*m*	0 μM AziP*m*	10 μM AziP*m*	20 μM AziP*m*	50 μM AziP*m*	MS/MS Total Count
AEDNADTLAL^{101}VFEAPNQEK	0	15	21	16	73
CAGNEDIITL^{90}R	0	22	20	12	62
DLSHI^{154}DAVVISCAK	0	20	14	7	40
Y^{211}LNFFTK	0	14	11	9	31

Figure 3. Interaction of AMA and anesthetics at a hydrophobic site near L90 and L101. (A) Emission spectra of 100 nM AMA equilibrated with 10 μM PCNA and the indicated propofol concentrations. Propofol caused a dose-dependent decrease in AMA fluorescence, which indicated AMA unbinding PCNA and returning to the aqueous solution. (B) Displacement of AMA from PCNA by propofol, as determined from the data in (A). The peak fluorescence intensity of AMA at each propofol concentration was normalized to the intensity of AMA bound to PCNA in the absence of competitor. Data points represent mean ± standard error. (C) Emission spectra of 100 nM AMA equilibrated with 10 μM PCNA and the indicated sevoflurane concentrations. (D) Displacement of AMA from PCNA by sevoflurane, as determined from the data in (C). (E) Table of peptides that were photolabeled during experiments with different concentrations of AziP*m*. Spectra can be found in Figure S4. (F) Left: location of AziP*m*-photolabeled residues on a PCNA trimer with the interdomain connecting loops (IDCL) also indicated as orange asterisks. Right: a side view of the structure (only two subunits are shown for clarity). (G) Emission spectra of 100 nM AMA equilibrated with 10 μM of the indicated PCNA variants. (H) Relationship between the peak emission wavelength of AMA when bound to the PCNA variants in (G) and the hydrophobicity of the residue substituted at position 90 or 101. Residue hydrophobicity scores were reported elsewhere.[44] (I) Binding isotherm showing the interaction of AMA with recombinant PCNA(L90I). (J) Displacement of AMA from PCNA(L90I) by sevoflurane.

To gain insight into the location of the AMA binding site on PCNA, we performed photoaffinity labeling using AziP*m*, a photoactivatable analog of propofol with similar physicochemical properties (Figure 1).[36] The diazirine of AziP*m* undergoes photolysis upon exposure to long-wave UV to yield a reactive carbene that covalently attaches the ligand to its protein binding sites, which reliably overlap with sites for propofol and AMA.[27,36,49,75] After photolabeling PCNA with AziP*m*, LC-MS/MS was used to identify four amino acids on PCNA that interacted with the anesthetic ligand (Figure 3E). This was evident because the photolabeled residues contained 216-Da mass shifts from an AziP*m* adduct (Figure S4). PCNA residues L90 and L101 were the most heavily labeled by AziP*m* (Figure 3E). These residues sit adjacent to each other in the folded protein within the same PCNA domain, and the leucine side chains were buried deep in the protein's hydrophobic core (Figure 3F). Photolabeled residues I154 and Y211 were more exposed to the solvent, with Y211 also serving as a phosphorylation site (Figure 3F).[76]

We hypothesized that mutating amino acids located within the AMA binding site would alter the dielectric environment of the site, thereby changing AMA's emission peak to report on the polarity of the site. We started by mutating residues L90 and L101 on recombinant PCNA proteins because of their strong interaction with the propofol photolabel. We found that the hydrophobicity of the amino acid substituted at position L90 or L101 determined the degree of AMA's blueshift, which supported the idea that the AMA binding site was being manipulated (Figures 3G and

3H). PCNA tolerated the incorporation of slightly less apolar residues at L90 and L101, but incorporation of charged or polar residues produced misfolded PCNA aggregates, as observed during size exclusion chromatography (e.g., lysine, serine, or glutamine mutations). Alanine substitutions at I154 and Y211 also resulted in misfolded protein; we cannot rule out that these photolabeled residues contributed to secondary sites for the small molecules, but we did not pursue them further. We also mutated residues in surface pockets on PCNA that yielded properly folded protein with the AMA binding signal unaffected (Figure S5), although hydrophilic AMA sites may not produce measurable changes in fluorescence. Nonetheless, this mutational analysis was consistent with the photolabeling results and indicated that AMA and the anesthetics interacted with L90 and L101 on PCNA.

Finally, we noted a peculiar PCNA variant from the above assays with an isomeric substitution in the putative AMA site (L90I) where the hydrophobicity of the side chain was marginally altered. The peak emission wavelength of AMA did not change when bound to PCNA(L90I), yet the fluorescence intensity was significantly elevated (Figure 3G). We determined a K_d of 125 μM for the interaction of AMA with PCNA(L90I), which was similar to wild-type PCNA (K_d = 84 μM) (Figure 3I). We displaced AMA from PCNA(L90I) using sevoflurane and determined an IC_{50} of 221 μM. Sevoflurane had 10-fold higher affinity for PCNA(L90I) compared with wild-type PCNA where it displaced AMA with an IC_{50} of 2.2 mM (Figure 3D). These changes in binding that resulted from subtle mutation of L90 confirmed this as the location of a highly apolar ligand site on PCNA.

Structure and dynamics of the cryptic PCNA binding site and ligand interactions

There are dozens of high-resolution PCNA structures where the homotrimer is bound to various polypeptides or ligands at a conserved surface pocket called the interdomain connecting loop (IDCL), through which PCNA orchestrates DNA metabolism.[31] The IDCL site was rather distant (>30 Å) from the anesthetic site at L90 and L101 (Figure 3F). Initially, we analyzed the crystal structure of apo-PCNA to mimic the conditions of our ligand binding assays (PDB: 1VYM),[51] and Fpocket identified >20 solvent-exposed surface cavities that were theoretically large enough to accommodate AMA, which has a molecular volume of 179 $Å^3$ (Figure S6). However, no binding pocket was identified near residues L90 and L101, which were packed with other hydrophobic side chains in the core of the protein (Figure 4A). We considered how protein movement can transiently open and close small cryptic sites that otherwise may not appear in static structures.[5,6,9,77,78] We ran two atomistic MD simulations of PCNA in water (1.5 μs total, Figure S7), and indeed, a space that was large enough to accommodate our ligands transiently appeared near L90 and L101 (Figure 4B). In part, this pocket was formed by the rotation of side chains out of the hydrophobic space (e.g., W28 and M68) (Figures 4A and 4B).

We examined the structure and dynamics of the cryptic anesthetic binding site that transiently appeared inside the hydrophobic core of PCNA and included residues L90 and L101 (Figure 4B). The site formed and disappeared in each subunit of the homotrimer independently and without correlation to other subunits (Figure S8). On average, a subunit contained a pocket that was large enough to bind anesthetics (>180 $Å^3$) for 20% of the simulations (Figure 4C). The site was lined with side chains from hydrophobic residues that packed into the protein core (Table S2), and we could detect no ions or water molecules entering the site. We also simulated PCNA(L90I) for 1.5 μs. The physicochemical features of the hydrophobic site were similar on PCNA(L90I), except that the anesthetic pocket was open more often than wild-type protein (25% of the simulation) and had a larger average volume (median pocket volume = 120 $Å^3$ for wild-type and 137 $Å^3$ for PCNA(L90I) (Figure 4C; Figure S8)). The L90 side chain frequently contacted more distant residues than the I90 side chain, which positions a terminal methyl group one carbon closer to the protein backbone (Figure S8).

To evaluate ligand binding to PCNA's cryptic site, we extracted structural snapshots from the simulations where the site was open in one or more subunits, and then, we docked AMA or sevoflurane onto wild-type PCNA or PCNA(L90I). AMA and sevoflurane could typically be docked into the internal pocket if its volume was sufficiently large with calculated binding free energies of ~6 kcal/mol (Figures 4D–4G). The predicted poses for AMA and sevoflurane were heterogeneous when docked to the PCNA proteins, and the shape of the pocket was also heterogeneous. Ligand-protein binding was mediated by hydrophobic or van der Waals interactions. We then simulated PCNA and PCNA(L90I) with each of the proteins bound to three molecules of sevoflurane (one molecule per subunit). Sevoflurane remained bound to all of the cryptic sites during 1-μs simulations, although the molecules frequently reoriented within the site (Figure S9 and Video S1). Two additional simulations of PCNA or PCNA(L90I) bound to a single AMA molecule produced similar behavior in which AMA remained in the site with occasional reorientation (Figure S9 and Video S2). Furthermore, another simulation with AMA initially docked to a different PCNA(L90I) starting structure showed AMA unbinding after ~310 ns and returning to the aqueous solvent, thereby providing a plausible entry/exit route to the protein's core (Figure S9).

Biophysical effects of ligand binding to the cryptic site

We considered how ligand binding to the cryptic site could affect PCNA stability, dynamics, and activity. We focused

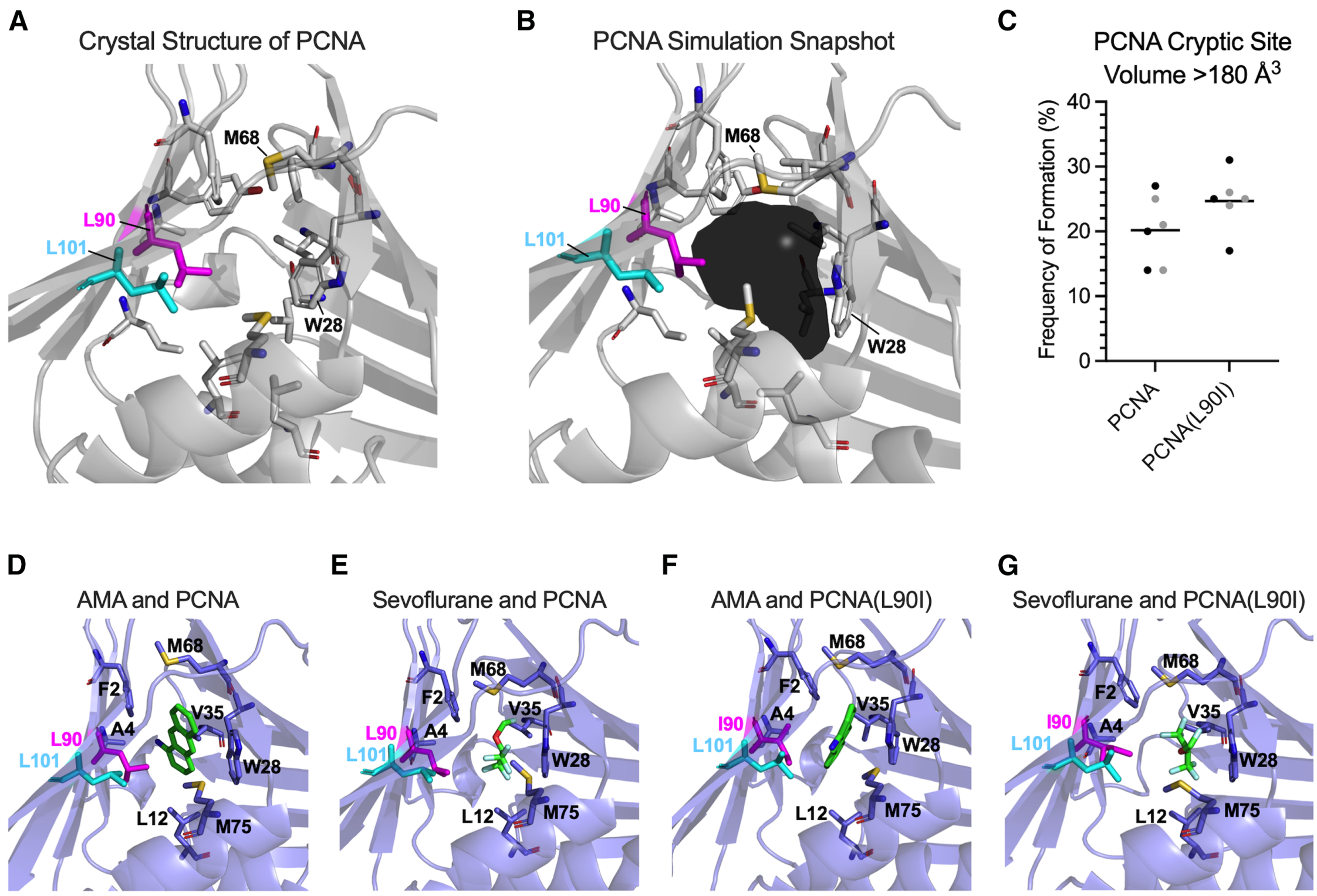

Figure 4. A cryptic binding site for small molecules in the hydrophobic core of PCNA. (A) View of residues near L90 and L101 in the crystal structure of apo-PCNA (PDB: 1VYM). (B) View of residues near L90 and L101 from a simulation snapshot of PCNA with the Fpocket-identified internal cavity shown in black. (C) Percent of MD simulation that each pocket formed near residues L90 and L101 that was large enough to accommodate general anesthetic ligands (black dots, three subunits from 1-μs simulation; gray dots, three subunits from 0.5-μs simulation; black bar, mean). The analysis was performed with MDpocket. (D) Highest scoring pose of AMA docked to PCNA. (E) Highest scoring pose of sevoflurane docked to PCNA. (F) Highest scoring pose of AMA docked to PCNA(L90I). The plane of the ligand is perpendicular to the plane of panel. (G) Highest scoring pose of sevoflurane docked to PCNA(L90I).

our studies on sevoflurane because we could achieve high occupancy of PCNA sites due to its higher solubility than AMA, which was limited to only ∼33 μM.[22] We equilibrated wild-type PCNA or PCNA(L90I) with 2.5 mM sevoflurane to assess how the anesthetic affected the thermostability of the protein. This ligand concentration was sufficient to occupy ∼53% of available cryptic sites in the wild-type protein and ∼92% in the mutant based on their different affinities for sevoflurane, and thermostability was assessed by monitoring the change in PCNA's intrinsic fluorescence upon denaturation. The melting temperature (T_m) of wild-type PCNA increased 1.6°C in the presence of sevoflurane, and the T_m of PCNA(L90I) increased by 3.7°C when bound to the ligand (Figure 5A; Figure S10). As a negative control, we found that sevoflurane reduced the T_m of UNG2, which lacks a lipophilic binding site for AMA (Figures 2A; Table 1), by ∼1°C, indicating weak destabilization of its folded structure (Figure S10).

Interestingly, a naturally occurring genetic variation in human PCNA yields the protein PCNA(C148S), which was reported to be a thermolabile homotrimer and the cause of human disorders that are characterized by impaired DNA replication and repair.[66] The destabilizing C148S mutation and the cryptic site are sterically separated and distant at ∼21 Å apart; nonetheless, we hypothesized that ligand interactions in the cryptic site could reverse the thermosensitive phenotype of PCNA(C148S). We confirmed that the T_m of PCNA(C148S) was 1.8°C lower than wild-type PCNA (Figure 5A; Figure S10). PCNA(C148S) interacted with AMA and sevoflurane with a similar affinity as wild-type PCNA (Figure S10), and the dielectric constant of its cryptic binding site was unaltered by the mutation ($\varepsilon = 4$). Sevoflurane binding increased the T_m of PCNA(C148S) by 3.3°C, almost matching the T_m of wild-type PCNA bound to the ligand (Figure 5A; Figure S10). This demonstrated that ligand interaction in the cryptic site can overcome a long-range destabilizing protein mutation, although it is unclear whether stabilization and destabilization arise through the same mechanistic pathway.

Although PCNA functions as a homotrimer, its subunits can dissociate and reassociate in solution with a K_d value of ∼21 nM.[79,80] Because sevoflurane stabilized the protein against unfolding, we thought that the ligand might enhance the association of its individual subunits within the

homotrimer. We developed a strategy to measure subunit exchange between different PCNA trimers. Briefly, untagged recombinant PCNA was mixed with a tagged PCNA variant that contained a 6xHis tag and SpyTag on its N terminus ("SpyT-His-PCNA") (Figure 5B).[81] Over time, untagged PCNA subunits exchanged into assemblies with SpyT-His-PCNA (Figure 5B). We quantified the level of untagged PCNA that pulled down with SpyT-His-PCNA on Ni^{2+} resin; before analysis by SDS-PAGE, we covalently reacted the SpyTag with SpyCatcher to improve the resolution of the protein bands on the gel (Figure 5C).[81] If untagged PCNA and SpyT-His-PCNA mixed and distributed evenly to an equilibrium during the course of the assay, then the molar ratio of untagged/tagged PCNA in the final pull-down would be 3/4 or 0.75 (see materials and methods).

For wild-type PCNA, the rate of subunit exchange followed first-order kinetics ($k_{obs} = 0.05\ min^{-1}$) and was within range of a rate determined for the exchange of yeast PCNA subunits under slightly different conditions.[34] It is probable that single subunit exchanges dominated in the assay.[34,79] For assays conducted at 37°C, the molar ratio of untagged/tagged PCNA in the final pull-down plateaued at 0.86 after ~90 min (Figures 5D and 5E, blue Y_{max} value), which was close to the ideal value of 0.75, indicating complete mixing of the PCNA subunits. We determined that sevoflurane reduced the level of subunit exchange that occurred between untagged PCNA and SpyT-His-PCNA (Figures 5D and 5E, purple Y_{max} value). This implied that the pool of PCNA that was exchanging subunits was reduced in the presence of sevoflurane. As a positive control, we conducted experiments on ice where the homotrimer was very stable and the pool of exchanging subunits declined further (Figures 5D and 5E, green Y_{max} value), which was consistent with a previous report.[79] The PCNA(L90I) variant behaved similarly to wild-type PCNA at 37°C (Figures 5F and 5G). Sevoflurane reduced the rate and level of subunit exchange that occurred in the mutant to a greater degree than wild-type PCNA, likely due to greater occupancy of the cryptic sites (Figures 5F and 5G).

Finally, we confirmed that the cryptic binding site was functionally independent from the IDCL site where PCNA binds to other proteins. We used fluorescence anisotropy-based binding assays to determine that sevoflurane and propofol did not affect the interaction of PCNA with full-length UNG2 (Figure S11).[40] We found that propofol did not affect PCNA binding to a peptide called Pogo-Ligase that binds to the IDCL site (Figure S11).[40,82,83] We found that sevoflurane did not affect the interaction of PCNA with a p15 peptide that binds to the same IDCL site, but with extended interactions (Figure S11).[84] Last, we examined whether sevoflurane binding interfered with the loading of PCNA onto DNA by RFC, which is driven by ATP hydrolysis and is also mediated through IDCL site interactions.[85,86] Sevoflurane did not affect the activity of RFC or its ability to load PCNA onto DNA (Figure S12).

Conservation of internal hydrophobic sites in diverse sliding clamps

We analyzed ~100 homologous PCNA and β clamp structures from humans, yeast, and prokaryotic organisms to test the hypothesis that the hydrophobic pocket is a conserved structural feature of the proteins, along with its variable volume. Many of these PCNA/β clamp proteins were bound to proteins, ligands, or DNA or may have contained point mutations, but nonetheless, they shared a homologous fold. Indeed, the hydrophobic pocket could be observed within the cores of various experimental protein structures from both X-ray crystallography and cryoelectron microscopy (Figures 6A–6D; Table S3). Some β clamp proteins, including human PCNA, occasionally featured internal pockets in adjacent pseudodomains (Figure 6C; Table S3). Even AlphaFold models of PCNA and PCNA(L90I) exhibited variable pocket volumes within subunits of the same homotrimer, and in the case of wild-type PCNA, the AlphaFold-predicted pockets were too small in size for the ligands examined in this study (Figure 6D). This analysis supports the idea that a localized hydrophobic space, dynamic in volume, is a conserved feature of this protein family. Additionally, the presence or detectability of the site may be dependent on many factors. As an example, for the human experimental structures, we found a significant relationship where lower resolution structures tended to provide larger pocket volumes (Figure S13). How the sites were affected by other factors such as DNA or protein interactions (including crystal contacts), rotameric conformations in the final model,[87,88] and user-defined algorithm thresholds (e.g., pocket detection cutoffs) remains to be systematically evaluated.

DISCUSSION

Using AMA as a small fluorescent probe, we identified a cryptic site in the core of human PCNA that can accommodate general anesthetics and other ligands with similar chemical features. The site forms because of packing defects among hydrophobic side chains and because of their spontaneous movement within the pocket. More than a dozen residues contributed to the formation of the site, which continually changed its shape and size during MD simulations, yet the site was sensitive to subtle perturbations like point mutations. We found that the site existed transiently as empty space in the protein core that weakened the strength of the PCNA oligomer; ligand binding to the site stabilized the PCNA trimer and protected the protein from thermal denaturation. Interestingly, after conducting DNA replication and repair activities at replication forks, some fraction of trimeric PCNA spontaneously dissociates to unload itself from dsDNA,[34,35] which might be tuned or influenced through this binding site. Finally, homologous β clamp proteins from yeast and prokaryotes have a similar

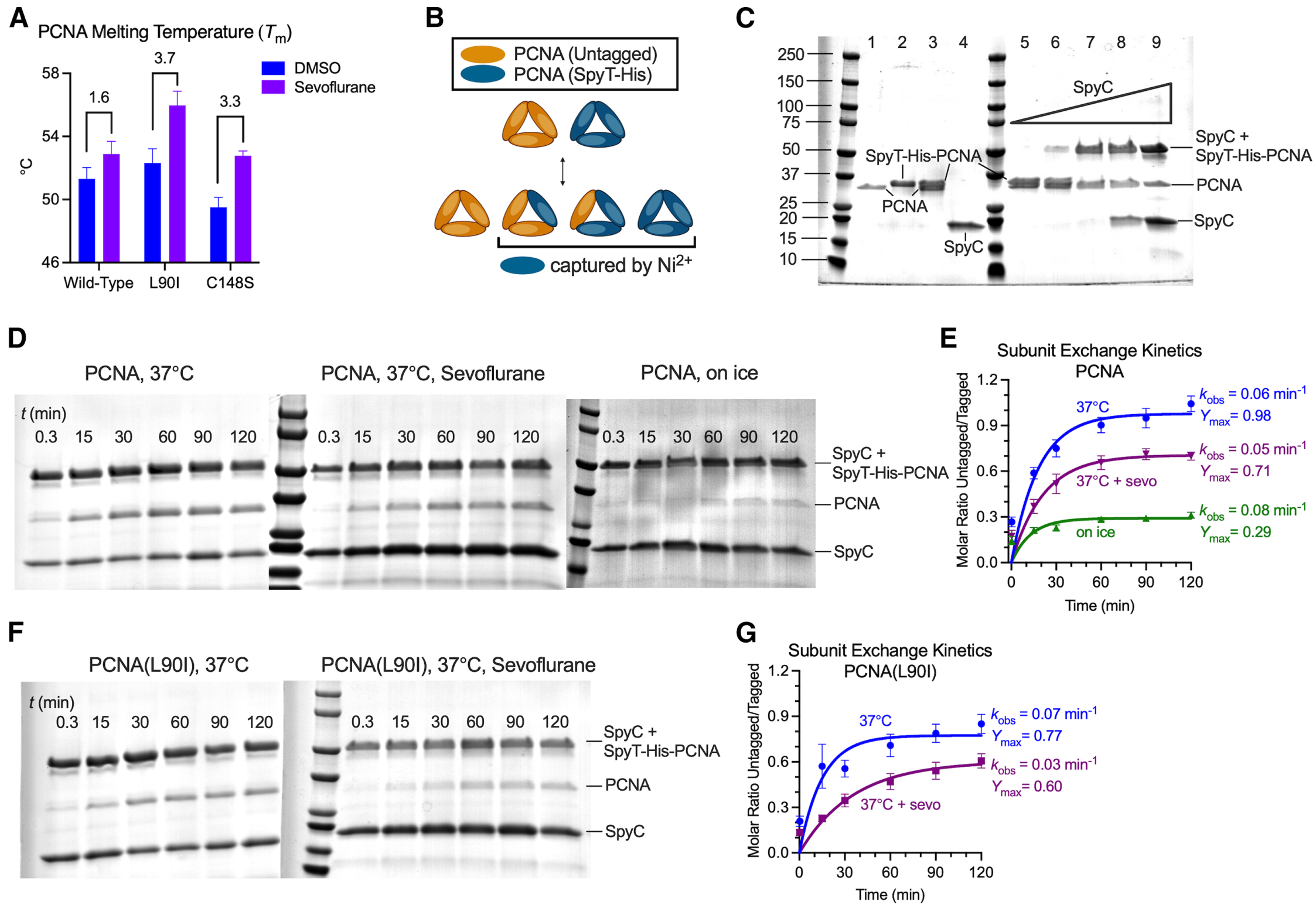

Figure 5. Cryptic site occupancy stabilizes the structure of the PCNA trimer. (A) Melting temperatures of recombinant PCNA proteins increased in the presence of 2.5 mM sevoflurane. (B) Scheme for subunit exchange assays where untagged PCNA exchanges subunits with a recombinant PCNA variant containing a SpyTag and 6xHis tag (SpyT-His), which can be captured on Ni^{2+} resin with accompanying subunits. (C) Coomassie-stained SDS-PAGE gel showing components of PCNA subunit exchange assays and the reaction of SpyT-His-PCNA with SpyCatcher. Lane 1, 5 μM (untagged) PCNA; lane 2, 5 μM SpyT-His-PCNA; lane 3, 5 μM PCNA +5 μM SpyT-His-PCNA; lane 4, 20 μM SpyCatcher; lanes 5–9 all contained 5 μM PCNA +5 μM SpyT-His-PCNA along with the following SpyCatcher concentrations: lane 5, 0 μM; lane 6, 1 μM; lane 7, 5 μM; lane 8, 10 μM; lane 9, 20 μM. (D) Representative Coomassie-stained gels from subunit exchange assays using PCNA at different temperatures with and without 2.5 mM sevoflurane. Over time, increasing amounts of untagged PCNA associated with SpyT-His-PCNA, which were captured together on Ni^{2+} resin before being eluted. The eluate was then reacted with SpyCatcher and run on the gel. (E) Kinetics of PCNA subunit exchange for the assay conditions shown in (D). Every data point represents mean ± standard error from three to five independent assays. (F) Coomassie-stained gels from subunit exchange assays using PCNA(L90I) with and without 2.5 mM sevoflurane. (G) Kinetics of PCNA subunit exchange for the assay conditions shown in (F). Every data point represents mean ± standard error from three to five independent assays.

pocket that forms in their hydrophobic core, indicating that this feature is conserved across the fold.

Related to cryptic site mutagenesis, the isomeric variant PCNA(L90I) had physical changes in pocket architecture and pharmacology compared with wild-type PCNA. Using BLAST and other tools,[89] we were unable to find a naturally occurring vertebrate PCNA with an isoleucine at that position, suggesting a selective pressure to retain the leucine. Notably, the emergence of isoleucine at that position in several invertebrates was accompanied by additional substitutions in neighboring pocket residues (e.g., *Daphnia pulex* or *Littorina saxatilis*). Together, these observations support the idea that the cryptic site of PCNA evolved to tune the activity of the DNA binding protein, rather than being a passive by-product of protein folding. A more refined framework is needed for understanding the evolutionary conservation and divergence of site features with regard to protein sequence, structure, and function. Our biochemical assays showed limited crosstalk between the cryptic site and the highly conserved IDCL site. However, examining the context dependence of internal pockets in proteins from various organisms must be approached cautiously with static experimental and *de novo* models that do not capture the full range of structural sampling observed in solution or simulations or that may bias certain states due to experimental parameters or training data sets.

Our fluorescence experiments measured the dielectric constant of AMA binding sites on proteins including its hydrophobic site in the core of PCNA (ε = 4), which was almost as apolar as cyclohexane (ε = 2). Dielectric constant is a parameter of polarizability, which is critical for calibrating computational models of electrostatics, solvation, and pK_a calculations.[11] Because the dielectric environment governs how charges and dipoles interact within a protein, it

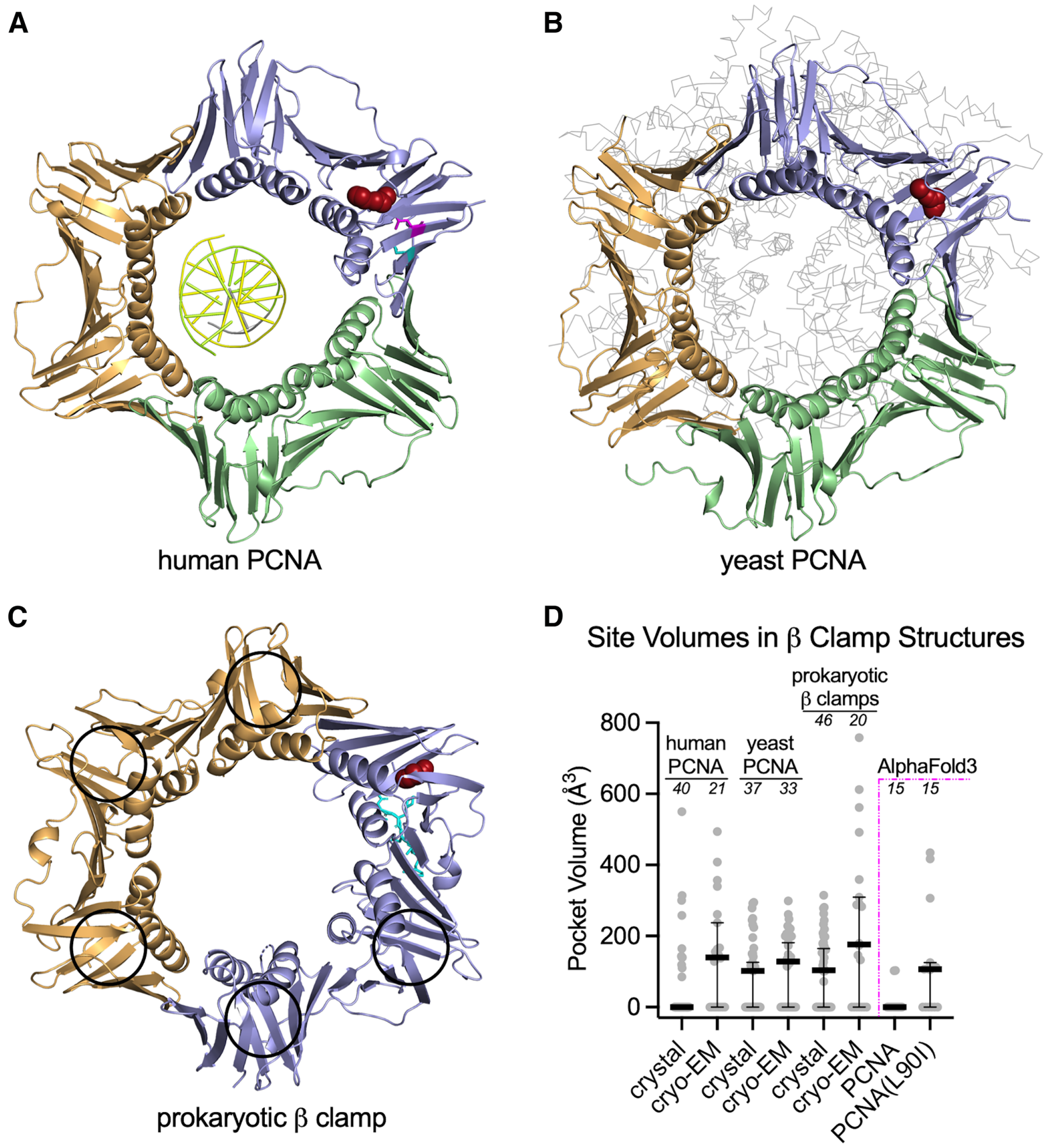

Figure 6. The hydrophobic site is conserved across the PCNA/β clamp fold. (A–C) Representative structures where we identified internal hydrophobic sites with variable volumes at analogous locations. Identified pockets are colored deep red spheres in the images. (A) X-ray crystal structure of human PCNA bound to DNA with L90 and L101 colored magenta and cyan, respectively (PDB: 6GIS). (B) X-ray crystal structure of yeast PCNA bound to replication factor C, which is shown as gray ribbons bound to the other side of the protein (PDB: 1SXJ). (C) X-ray crystal structure of the dimeric sliding clamp from the prokaryote *Pseudomonas aeruginosa* bound to a synthetic peptide molecule, colored cyan (PDB: 4TSZ). In panel (C), we also circled the five other pseudodomain locations in the β clamp fold where internal pockets may exist on select proteins (Table S3). (D) Internal hydrophobic site volumes for every subunit in 85 experimental PCNA or β clamp structures, along with data for five human PCNA or PCNA(L90I) AlphaFold3 models. Every gray point represents a single subunit, and the total number of analyzed subunits is written in italics above each data column. The black bar represents the median with the 95% confidence interval as error bars.

directly influences folding stability, conformational transitions, and ligand recognition.[90–92] Previously, an environmentally sensitive fluorescent amino acid, which was site specifically incorporated on the model protein GB1, reported an ε of 6 in a buried hydrophobic domain and a range of ε values at other sites, which were consistent with our measurements.[72] The small size, structural plasticity, and hydrophobicity of our site reduced the possibility for high-affinity ligand interactions. Nonetheless, general anesthetics are evidence that these sites can be targeted for therapeutic value, and even weak binding ligands can offset destabilizing protein mutations by lowering the free energy of their folded state.[93] The finding that sevoflurane had 10-fold higher affinity for PCNA(L90I) compared with wild-type PCNA, without an accompanying change in the dielectric environment of the site, also indicated that ε values alone were not sufficient to predict relative binding affinities for lipophilic ligands. Finally, the L90I substitution increased the fluorescence of AMA bound to the site compared with wild type. This could occur if the mutation increased the availability of the site, which was measured to a limited extent, but we also detected different interactions among residue side chains in the mutated cryptic site (Figure 4C; Figure S8). To some degree, ligand-protein interactions should change in the mutant cryptic site, supported by the change in sevoflurane affinity for PCNA(L90I), and changes in pocket geometry, ligand rigidity, or ligand orientation relative to nearby residues may affect AMA fluorescence intensity.

In conclusion, we used AMA as a fluorescence reporter of hydrophobic ligand sites to identify a cryptic binding site deep in the core of human PCNA. Ligands with similar physical and chemical features interact with PCNA at the site and alter its stability and dynamics. Although not explored here, it is possible that an endogenous ligand from the cell could bind to the site in humans or β clamp proteins from other organisms. As a general approach, we expect that screening or systematically probing proteins with fluorophores tuned to detect sites of specific sizes, shapes, or polarities could significantly expand the number of druggable sites in the proteome and uncover novel strategies to alter protein activities.

DATA AND CODE AVAILABILITY

The experimental data sets underlying this study are openly available on Zenodo at https://zenodo.org/records/19630221 or https://doi.org/10.5281/zenodo.19630221.

ACKNOWLEDGMENTS

This work was supported in part by National Institutes of Health grant R01GM135152 and New Jersey Health Foundation grant PC 215-24 awarded to B.P.W. Additional support came from National Institutes of Health grant R01GM135633 awarded to R.G.E. and a Foundation for Anesthesia Education and Research grant awarded to E.R.W. The authors thank Drs. Benjamin Garcia and Natarajan Bhanu for assistance with mass spectrometry experiments. The authors acknowledge the use of BioRender to prepare the online graphical abstract.

AUTHOR CONTRIBUTIONS

A.N.D. designed research, performed research, analyzed data, and wrote the manuscript; S.N.G. performed research; J.Y. performed research; D.E.J. performed research; J.F. performed research; E.R.W. performed research; R.G.E. contributed research tools; Z.L. contributed research tools and provided supervision; B.P.W. designed research, analyzed data, provided supervision, and wrote the manuscript. All authors reviewed the final manuscript.

DECLARATION OF INTERESTS

The authors declare no competing interests.

SUPPORTING CITATIONS

Refs. 94–111 appear only in the supplemental information.

SUPPORTING INFORMATION

Supplemental information can be found online at https://doi.org/10.1016/j.bpj.2026.05.027.

REFERENCES

1. Gaines, J. C., W. W. Smith, …, C. S. O'Hern. 2016. Random close packing in protein cores. *Phys. Rev. E.* 93:032415. https://doi.org/10.1103/PhysRevE.93.032415.
2. He, J., and I. J. Dmochowski. 2023. Local Xenon–Protein Interaction Produces Global Conformational Change and Allosteric Inhibition in Lysozyme. *Biochemistry.* 62:1659–1669. https://doi.org/10.1021/acs.biochem.3c00046.
3. Zhao, Z., N. A. Rudman, …, I. J. Dmochowski. 2022. Programming xenon diffusion in maltose-binding protein. *Biophys. J.* 121:4635–4643. https://doi.org/10.1016/j.bpj.2022.10.025.
4. Brunori, M., and Q. H. Gibson. 2001. Cavities and packing defects in the structural dynamics of myoglobin. *EMBO Rep.* 2:674–679. https://doi.org/10.1093/embo-reports/kve159.
5. Beglov, D., D. R. Hall, …, S. Vajda. 2018. Exploring the structural origins of cryptic sites on proteins. *Proc. Natl. Acad. Sci. USA.* 115:E3416–E3425. https://doi.org/10.1073/pnas.1711490115.
6. Cimermancic, P., P. Weinkam, …, A. Sali. 2016. CryptoSite: Expanding the Druggable Proteome by Characterization and Prediction of Cryptic Binding Sites. *J. Mol. Biol.* 428:709–719. https://doi.org/10.1016/j.jmb.2016.01.029.
7. Mizukoshi, Y., K. Takeuchi, …, I. Shimada. 2020. Targeting the cryptic sites: NMR-based strategy to improve protein druggability by controlling the conformational equilibrium. *Sci. Adv.* 6:eabd0480. https://doi.org/10.1126/sciadv.abd0480.
8. Frembgen-Kesner, T., and A. H. Elcock. 2006. Computational sampling of a cryptic drug binding site in a protein receptor: explicit solvent molecular dynamics and inhibitor docking to p38 MAP kinase. *J. Mol. Biol.* 359:202–214. https://doi.org/10.1016/j.jmb.2006.03.021.
9. Shan, Y., V. P. Mysore, …, D. E. Shaw. 2022. How does a small molecule bind at a cryptic binding site? *PLoS Comput. Biol.* 18:e1009817. https://doi.org/10.1371/journal.pcbi.1009817.
10. Prangé, T., M. Schiltz, …, R. Fourme. 1998. Exploring hydrophobic sites in proteins with xenon or krypton. *Proteins.* 30:61–73.
11. Li, L., C. Li, …, E. Alexov. 2013. On the Dielectric "Constant" of Proteins: Smooth Dielectric Function for Macromolecular Modeling and Its Implementation in DelPhi. *J. Chem. Theory Comput.* 9:2126–2136. https://doi.org/10.1021/ct400065j.
12. Sun, Z., A. E. Wakefield, …, S. Vajda. 2020. Structure-Based Analysis of Cryptic-Site Opening. *Structure.* 28:223–235.e2. https://doi.org/10.1016/j.str.2019.11.007.
13. Ludlow, R. F., M. L. Verdonk, …, H. Jhoti. 2015. Detection of secondary binding sites in proteins using fragment screening. *Proc. Natl. Acad. Sci. USA.* 112:15910–15915. https://doi.org/10.1073/pnas.1518946112.
14. Woll, K. A., S. Murlidaran, …, R. G. Eckenhoff. 2016. A Novel Bifunctional Alkylphenol Anesthetic Allows Characterization of γ-Aminobutyric Acid, Type A (GABAA), Receptor Subunit Binding Selectivity in Synaptosomes. *J. Biol. Chem.* 291:20473–20486. https://doi.org/10.1074/jbc.M116.736975.
15. Weiser, B. P., M. B. Kelz, and R. G. Eckenhoff. 2013. In vivo activation of azipropofol prolongs anesthesia and reveals synaptic targets. *J. Biol. Chem.* 288:1279–1285. https://doi.org/10.1074/jbc.M112.413989.
16. Emerson, D. J., B. P. Weiser, …, I. J. Dmochowski. 2013. Direct modulation of microtubule stability contributes to anthracene general anesthesia. *J. Am. Chem. Soc.* 135:5389–5398. https://doi.org/10.1021/ja311171u.
17. Zuzic, L., J. K. Marzinek, …, P. J. Bond. 2020. A Benzene-Mapping Approach for Uncovering Cryptic Pockets in Membrane-Bound Proteins. *J. Chem. Theory Comput.* 16:5948–5959. https://doi.org/10.1021/acs.jctc.0c00370.
18. Kimura, S. R., H. P. Hu, …, A. D. Favia. 2017. Deciphering Cryptic Binding Sites on Proteins by Mixed-Solvent Molecular Dynamics. *J. Chem. Inf. Model.* 57:1388–1401. https://doi.org/10.1021/acs.jcim.6b00623.
19. Wiebelhaus, N., N. Singh, …, M. C. Fitzgerald. 2022. Discovery of the Xenon-Protein Interactome Using Large-Scale Measurements of Protein Folding and Stability. *J. Am. Chem. Soc.* 144:3925–3938. https://doi.org/10.1021/jacs.1c11900.
20. Odom, O. W., B. B. Craig, and B. A. Hardesty. 1978. The conformation of the anticodon loop of yeast tRNAPhe in solution and on ribosomes. *Biopolymers.* 17:2909–2931. https://doi.org/10.1002/bip.1978.360171212.
21. Rotkiewicz, K., and Z. R. Grabowski. 1969. Excited states of aminoanthracenes. An experimental approach to electron density distribution. *Trans. Faraday Soc.* 65:3263–3278. https://doi.org/10.1039/TF9696503263.
22. Butts, C. A., J. Xi, …, I. J. Dmochowski. 2009. Identification of a fluorescent general anesthetic, 1-aminoanthracene. *Proc. Natl. Acad. Sci. USA.* 106:6501–6506. https://doi.org/10.1073/pnas.0810590106.
23. Paolini, S., F. Tanfani, …, P. Pelosi. 1999. Porcine odorant-binding protein: structural stability and ligand affinities measured by fourier-transform infrared spectroscopy and fluorescence spectroscopy. *Biochim. Biophys. Acta.* 1431:179–188.
24. Watanabe, S., K. i. Kumagai, …, T. Hoshi. 2000. Hydrogen Bond Formations of 1-Aminoanthracene in the Ground and Excited Electronic States with Protic Solvent Molecules. *BCSJ.* 73:1783–1789. https://doi.org/10.1246/bcsj.73.1783.
25. Wang, X., S. Wang, and H. Ma. 2008. Characterization of local polarity and structure of Cys121 domain in beta-lactoglobulin with a new thiol-specific fluorescent probe. *Analyst.* 133:478–484. https://doi.org/10.1039/b717230c.

26. Hu, J., L. Jiang, …, W.-H. Chen. 2025. Environmentally sensitive fluorescent probes for the visualization of selective proteins: Recent progress and biomedical applications. *Eur. J. Med. Chem.* 300:118158. https://doi.org/10.1016/j.ejmech.2025.118158.

27. Bi, D., J. Yang, …, B. P. Weiser. 2020. Substrate-Dependent Modulation of SIRT2 by a Fluorescent Probe, 1-Aminoanthracene. *Biochemistry.* 59:3869–3878. https://doi.org/10.1021/acs.biochem.0c00564.

28. Miller, P. S., and A. R. Aricescu. 2014. Crystal structure of a human $GABA_A$ receptor. *Nature.* 512:270–275. https://doi.org/10.1038/nature13293.

29. Yuki, K., W. Bu, …, R. Eckenhoff. 2013. Propofol shares the binding site with isoflurane and sevoflurane on leukocyte function-associated antigen-1. *Anesth. Analg.* 117:803–811. https://doi.org/10.1213/ANE.0b013e3182a00ae0.

30. Bu, W., L. M. Pereira, …, K. Yuki. 2014. Stereoselectivity of Isoflurane in Adhesion Molecule Leukocyte Function-Associated Antigen-1. *PLoS One.* 9:e96649. https://doi.org/10.1371/journal.pone.0096649.

31. Moldovan, G.-L., B. Pfander, and S. Jentsch. 2007. PCNA, the Maestro of the Replication Fork. *Cell.* 129:665–679. https://doi.org/10.1016/j.cell.2007.05.003.

32. Wendel, S. O., J. A. Snow, …, N. A. Wallace. 2023. The potential of PCNA inhibition as a therapeutic strategy in cervical cancer. *J. Med. Virol.* 95:e29244. https://doi.org/10.1002/jmv.29244.

33. Søgaard, C. K., and M. Otterlei. 2024. Targeting proliferating cell nuclear antigen (PCNA) for cancer therapy. *Adv. Pharmacol.* 100:209–246. https://doi.org/10.1016/bs.apha.2024.04.002.

34. Kumar, R., V. C. Nashine, …, T.-H. Lee. 2010. Stepwise loading of yeast clamp revealed by ensemble and single-molecule studies. *Proc. Natl. Acad. Sci. USA.* 107:19736–19741. https://doi.org/10.1073/pnas.1014139107.

35. Hedglin, M., S. K. Perumal, …, S. Benkovic. 2013. Stepwise assembly of the human replicative polymerase holoenzyme. *eLife.* 2:e00278. https://doi.org/10.7554/eLife.00278.

36. Hall, M. A., J. Xi, …, R. G. Eckenhoff. 2010. m-Azipropofol (AziPm) a photoactive analogue of the intravenous general anesthetic propofol. *J. Med. Chem.* 53:5667–5675. https://doi.org/10.1021/jm1004072.

37. Woll, K. A., W. Peng, …, R. G. Eckenhoff. 2017. Photoaffinity Ligand for the Inhalational Anesthetic Sevoflurane Allows Mechanistic Insight into Potassium Channel Modulation. *ACS Chem. Biol.* 12:1353–1362. https://doi.org/10.1021/acschembio.7b00222.

38. Torres-Cartas, S., Y. Martín-Biosca, …, M. J. Medina-Hernández. 2007. Biopartitioning micellar chromatography to predict mutagenicity of aromatic amines. *Eur. J. Med. Chem.* 42:1396–1402. https://doi.org/10.1016/j.ejmech.2007.02.022.

39. Gaubitz, C., X. Liu, …, B. A. Kelch. 2020. Structure of the human clamp loader reveals an autoinhibited conformation of a substrate-bound AAA+ switch. *Proc. Natl. Acad. Sci. USA.* 117:23571–23580. https://doi.org/10.1073/pnas.2007437117.

40. Weiser, B. P., J. T. Stivers, and P. A. Cole. 2017. Investigation of N-Terminal Phospho-Regulation of Uracil DNA Glycosylase Using Protein Semisynthesis. *Biophys. J.* 113:393–401. https://doi.org/10.1016/j.bpj.2017.06.016.

41. Hong, J. Y., J. Cassel, …, B. P. Weiser. 2021. High-Throughput Screening Identifies Ascorbyl Palmitate as a SIRT2 Deacetylase and Defatty-Acylase Inhibitor. *ChemMedChem.* 16:3484–3494. https://doi.org/10.1002/cmdc.202100343.

42. Henricksen, L. A., C. B. Umbricht, and M. S. Wold. 1994. Recombinant replication protein A: expression, complex formation, and functional characterization. *J. Biol. Chem.* 269:11121–11132.

43. Nikolovska-Coleska, Z., R. Wang, …, S. Wang. 2004. Development and optimization of a binding assay for the XIAP BIR3 domain using fluorescence polarization. *Anal. Biochem.* 332:261–273. https://doi.org/10.1016/j.ab.2004.05.055.

44. Monera, O. D., T. J. Sereda, …, R. S. Hodges. 1995. Relationship of sidechain hydrophobicity and alpha-helical propensity on the stability of the single-stranded amphipathic alpha-helix. *J. Pept. Sci.* 1:319–329. https://doi.org/10.1002/psc.310010507.

45. Savitzky, A., and M. J. E. Golay. 1964. Smoothing and Differentiation of Data by Simplified Least Squares Procedures. *Anal. Chem.* 36:1627–1639. https://doi.org/10.1021/ac60214a047.

46. Rappsilber, J., M. Mann, and Y. Ishihama. 2007. Protocol for micro-purification, enrichment, pre-fractionation and storage of peptides for proteomics using StageTips. *Nat. Protoc.* 2:1896–1906. https://doi.org/10.1038/nprot.2007.261.

47. Tyanova, S., T. Temu, and J. Cox. 2016. The MaxQuant computational platform for mass spectrometry-based shotgun proteomics. *Nat. Protoc.* 11:2301–2319. https://doi.org/10.1038/nprot.2016.136.

48. Weiser, B. P., W. Bu, …, R. G. Eckenhoff. 2014. Sites and functional consequence of VDAC–alkylphenol anesthetic interactions. *FEBS (Fed. Eur. Biochem. Soc.) Lett.* 588:4398–4403. https://doi.org/10.1016/j.febslet.2014.10.009.

49. Weiser, B. P., and R. G. Eckenhoff. 2015. Propofol inhibits SIRT2 deacetylase through a conformation-specific, allosteric site. *J. Biol. Chem.* 290:8559–8568. https://doi.org/10.1074/jbc.M114.620732.

50. White, E. R., D. M. Leace, …, R. G. Eckenhoff. 2021. Synthesis and Characterization of a Diazirine-Based Photolabel of the Nonanesthetic Fropofol. *ACS Chem. Neurosci.* 12:176–183. https://doi.org/10.1021/acschemneuro.0c00667.

51. Kontopidis, G., S.-Y. Wu, …, M. D. Walkinshaw. 2005. Structural and biochemical studies of human proliferating cell nuclear antigen complexes provide a rationale for cyclin association and inhibitor design. *Proc. Natl. Acad. Sci. USA.* 102:1871–1876. https://doi.org/10.1073/pnas.0406540102.

52. Jumper, J., R. Evans, …, D. Hassabis. 2021. Highly accurate protein structure prediction with AlphaFold. *Nature.* 596:583–589. https://doi.org/10.1038/s41586-021-03819-2.

53. Case, D. A., H. M. Aktulga, …, K. M. Merz, Jr. 2023. AmberTools. *J. Chem. Inf. Model.* 63:6183–6191. https://doi.org/10.1021/acs.jcim.3c01153.

54. Tian, C., K. Kasavajhala, …, C. Simmerling. 2020. ff19SB: Amino-Acid-Specific Protein Backbone Parameters Trained against Quantum Mechanics Energy Surfaces in Solution. *J. Chem. Theory Comput.* 16:528–552. https://doi.org/10.1021/acs.jctc.9b00591.

55. Izadi, S., R. Anandakrishnan, and A. V. Onufriev. 2014. Building Water Models: A Different Approach. *J. Phys. Chem. Lett.* 5:3863–3871. https://doi.org/10.1021/jz501780a.

56. Humphrey, W., A. Dalke, and K. Schulten. 1996. VMD: Visual molecular dynamics. *J. Mol. Graph.* 14:33–38. https://doi.org/10.1016/0263-7855(96)00018-5.

57. Le Guilloux, V., P. Schmidtke, and P. Tuffery. 2009. Fpocket: An open source platform for ligand pocket detection. *BMC Bioinf.* 10:168. https://doi.org/10.1186/1471-2105-10-168.

58. Schmidtke, P., A. Bidon-Chanal, …, X. Barril. 2011. MDpocket: open-source cavity detection and characterization on molecular dynamics trajectories. *Bioinformatics.* 27:3276–3285. https://doi.org/10.1093/bioinformatics/btr550.

59. Abramson, J., J. Adler, …, J. M. Jumper. 2024. Accurate structure prediction of biomolecular interactions with AlphaFold 3. *Nature.* 630:493–500. https://doi.org/10.1038/s41586-024-07487-w.

60. Johnston, R. C., K. Yao, …, J. C. Shelley. 2023. Epik: pKa and Protonation State Prediction through Machine Learning. *J. Chem. Theory Comput.* 19:2380–2388. https://doi.org/10.1021/acs.jctc.3c00044.

61. Madhavi Sastry, G., M. Adzhigirey, …, W. Sherman. 2013. Protein and ligand preparation: parameters, protocols, and influence on virtual screening enrichments. *J. Comput. Aided Mol. Des.* 27:221–234. https://doi.org/10.1007/s10822-013-9644-8.

62. Lu, C., C. Wu, …, E. D. Harder. 2021. OPLS4: Improving Force Field Accuracy on Challenging Regimes of Chemical Space. *J. Chem. Theory Comput.* 17:4291–4300. https://doi.org/10.1021/acs.jctc.1c00302.

63. Yang, Y., K. Yao, …, S. V. Jerome. 2021. Efficient Exploration of Chemical Space with Docking and Deep Learning. *J. Chem. Theory Comput.* 17:7106–7119. https://doi.org/10.1021/acs.jctc.1c00810.

64. Friesner, R. A., J. L. Banks, …, P. S. Shenkin. 2004. Glide: a new approach for rapid, accurate docking and scoring. 1. Method and assessment of docking accuracy. *J. Med. Chem.* 47:1739–1749. https://doi.org/10.1021/jm0306430.

65. Friesner, R. A., R. B. Murphy, …, D. T. Mainz. 2006. Extra precision glide: docking and scoring incorporating a model of hydrophobic enclosure for protein-ligand complexes. *J. Med. Chem.* 49:6177–6196. https://doi.org/10.1021/jm051256o.

66. Magrino, J., V. Munford, …, B. A. Kelch. 2023. A thermosensitive PCNA allele underlies an ataxia-telangiectasia-like disorder. *J. Biol. Chem.* 299:104656. https://doi.org/10.1016/j.jbc.2023.104656.

67. Schindelin, J., I. Arganda-Carreras, …, A. Cardona. 2012. Fiji: an open-source platform for biological-image analysis. *Nat. Methods.* 9:676–682. https://doi.org/10.1038/nmeth.2019.

68. Lide, D. R. 2005. CRC Handbook of Chemistry and Physics, Internet Version 2005. CRC Press, Boca Raton, FL.

69. Schulman, S. G., P. J. Kovi, …, D. Carter. 1973. Electronic Spectra and Electronic Structures of Aminoanthracenes. *J. Pharmaceut. Sci.* 62:1823–1826. https://doi.org/10.1002/jps.2600621118.

70. Martynov, I. Y., A. B. Demyashkevich, …, M. G. Kuz'min. 1977. Proton Transfer Reactions in the Excited Electronic States of Aromatic-Molecules. *Russ. Chem. Rev.* 46:1–15. https://doi.org/10.1070/RC1977v046n01ABEH002116.

71. Vander Donckt, E., and G. Porter. 1968. Acidity constants of anthracene derivatives in singlet and triplet excited states. *Trans. Faraday Soc.* 64:3218–3223. https://doi.org/10.1039/TF9686403218.

72. Cohen, B. E., T. B. McAnaney, …, L. Y. Jan. 2002. Probing Protein Electrostatics with a Synthetic Fluorescent Amino Acid. *Science.* 296:1700–1703. https://doi.org/10.1126/science.1069346.

73. Nath, S., B. Bhattacharya, …, T. S. Singh. 2022. Solvent Effects on the Photophysical Properties of a Donor–acceptor Based Schiff Base. *J. Fluoresc.* 32:1321–1336. https://doi.org/10.1007/s10895-022-02905-6.

74. Larsen, A. H., N. T. Johansen, …, S. R. Midtgaard. 2021. Lipid-bound ApoE3 self-assemble into elliptical disc-shaped particles. *Biochim. Biophys. Acta Biomembr.* 1863:183495. https://doi.org/10.1016/j.bbamem.2020.183495.

75. Weiser, B. P., K. A. Woll, …, R. G. Eckenhoff. 2014. Mechanisms revealed through general anesthetic photolabeling. *Curr. Anesthesiol. Rep.* 4:57–66. https://doi.org/10.1007/s40140-013-0040-7.

76. Wang, S.-C., Y. Nakajima, …, M.-C. Hung. 2006. Tyrosine phosphorylation controls PCNA function through protein stability. *Nat. Cell Biol.* 8:1359–1368. https://doi.org/10.1038/ncb1501.

77. Haloi, N., E. Karlsson, …, E. Lindahl. 2025. Discovering cryptic pocket opening and binding of a stimulant derivative in a vestibular site of the 5-HT3A receptor. *Sci. Adv.* 11:eadr0797. https://doi.org/10.1126/sciadv.adr0797.

78. Meller, A., M. Ward, …, G. R. Bowman. 2023. Predicting locations of cryptic pockets from single protein structures using the PocketMiner graph neural network. *Nat. Commun.* 14:1177. https://doi.org/10.1038/s41467-023-36699-3.

79. Jónsson, Z. O., V. N. Podust, …, U. Hübscher. 1995. Tyrosine 114 is essential for the trimeric structure and the functional activities of human proliferating cell nuclear antigen. *EMBO J.* 14:5745–5751. https://doi.org/10.1002/j.1460-2075.1995.tb00261.x.

80. Yao, N., J. Turner, …, M. O'Donnell. 1996. Clamp loading, unloading and intrinsic stability of the PCNA, beta and gp45 sliding clamps of human, E. coli and T4 replicases. *Genes Cells.* 1:101–113. https://doi.org/10.1046/j.1365-2443.1996.07007.x.

81. Zakeri, B., J. O. Fierer, …, M. Howarth. 2012. Peptide tag forming a rapid covalent bond to a protein, through engineering a bacterial adhesin. *Proc. Natl. Acad. Sci. USA.* 109:E690–E697. https://doi.org/10.1073/pnas.1115485109.

82. Pedley, A. M., M. A. Lill, and V. J. Davisson. 2014. Flexibility of PCNA-protein interface accommodates differential binding partners. *PLoS One.* 9:e102481. https://doi.org/10.1371/journal.pone.0102481.

83. Greenwood, S. N., R. G. Belz, and B. P. Weiser. 2022. A Conserved Mechanism for Hormesis in Molecular Systems. *Dose Response.* 20:15593258221109335. https://doi.org/10.1177/15593258221109335.

84. De Biasio, A., A. I. de Opakua, …, F. J. Blanco. 2015. Structure of p15PAF–PCNA complex and implications for clamp sliding during DNA replication and repair. *Nat. Commun.* 6:6439. https://doi.org/10.1038/ncomms7439.

85. Bowman, G. D., M. O'Donnell, and J. Kuriyan. 2004. Structural analysis of a eukaryotic sliding DNA clamp-clamp loader complex. *Nature.* 429:724–730. https://doi.org/10.1038/nature02585.

86. Tsurimoto, T., and B. Stillman. 1989. Purification of a cellular replication factor, RF-C, that is required for coordinated synthesis of leading and lagging strands during simian virus 40 DNA replication in vitro. *Mol. Cell Biol.* 9:609–619. https://doi.org/10.1128/mcb.9.2.609-619.1989.

87. Maisuradze, G. G., A. Thakur, …, R. M. Levy. 2025. Predicting side chain conformations in folded proteins by AlphaFold: Perspective and challenges. *Biophys. J.* 124:3755–3771. https://doi.org/10.1016/j.bpj.2025.09.030.

88. Meller, A., S. Bhakat, …, G. R. Bowman. 2023. Accelerating Cryptic Pocket Discovery Using AlphaFold. *J. Chem. Theory Comput.* 19:4355–4363. https://doi.org/10.1021/acs.jctc.2c01189.

89. Camacho, C., G. Coulouris, …, T. L. Madden. 2009. BLAST+: architecture and applications. *BMC Bioinf.* 10:421. https://doi.org/10.1186/1471-2105-10-421.

90. Affleck, R., C. A. Haynes, and D. S. Clark. 1992. Solvent dielectric effects on protein dynamics. *Proc. Natl. Acad. Sci. USA.* 89:5167–5170. https://doi.org/10.1073/pnas.89.11.5167.

91. Sato, T., J. Ohnuki, and M. Takano. 2016. Dielectric Allostery of Protein: Response of Myosin to ATP Binding. *J. Phys. Chem. B.* 120:13047–13055. https://doi.org/10.1021/acs.jpcb.6b10003.

92. Pitera, J. W., M. Falta, and W. F. van Gunsteren. 2001. Dielectric properties of proteins from simulation: the effects of solvent, ligands, pH, and temperature. *Biophys. J.* 80:2546–2555. https://doi.org/10.1016/S0006-3495(01)76226-1.

93. Boeckler, F. M., A. C. Joerger, …, A. R. Fersht. 2008. Targeted rescue of a destabilized mutant of p53 by an in silico screened drug. *Proc. Natl. Acad. Sci. USA.* 105:10360–10365. https://doi.org/10.1073/pnas.0805326105.

94. Kmiecik, D., and J. R. Albani. 2010. Effect of 1-aminoanthracene (1-AMA) binding on the structure of three lipocalin proteins, the dimeric β lactoglobulin, the dimeric odorant binding protein and the monomeric α1-acid glycoprotein. Fluorescence spectra and lifetimes studies. *J. Fluoresc.* 20:973–983. https://doi.org/10.1007/s10895-010-0643-z.

95. Woll, K. A., B. P. Weiser, …, R. G. Eckenhoff. 2015. Role for the propofol hydroxyl in anesthetic protein target molecular recognition. *ACS Chem. Neurosci.* 6:927–935. https://doi.org/10.1021/acschemneuro.5b00078.

96. Bu, W., R. Liu, …, R. G. Eckenhoff. 2012. Ferritin couples iron and fatty acid metabolism. *FASEB J.* 26:2394–2400. https://doi.org/10.1096/fj.11-198853.

97. Miled, N., L. Berti-Dupuis, …, R. Verger. 2003. In vitro lipolysis by human pancreatic lipase is specifically abolished by its inactive forms. *Biochim. Biophys. Acta.* 1645:241–246. https://doi.org/10.1016/s1570-9639(02)00537-x.

98. Roussel, A., N. Miled, …, C. Cambillau. 2002. Crystal Structure of the Open Form of Dog Gastric Lipase in Complex with a Phosphonate Inhibitor. *J. Biol. Chem.* 277:2266–2274. https://doi.org/10.1074/jbc.M109484200.

99. Gu, Y., Q. Liu, …, D. Lin. 2013. Characterization of the oligomerization and ligand-binding properties of recombinant rat lipocalin 11. *Biochim. Biophys. Acta.* 1834:1–7. https://doi.org/10.1016/j.bbapap.2012.08.018.

100. Johansson, J. S., G. A. Manderson, …, R. G. Eckenhoff. 2005. Binding of the volatile general anesthetics halothane and isoflurane to a mammalian beta-barrel protein. *FEBS J.* 272:573–581. https://doi.org/10.1111/j.1742-4658.2004.04500.x.

101. Meillour, P. N.-L., P. Lagant, …, J.-C. Michalski. 2009. Phenylalanine 35 and tyrosine 82 are involved in the uptake and release of ligand by porcine odorant-binding protein. *Biochim. Biophys. Acta, Proteins Proteomics.* 1794:1142–1150. https://doi.org/10.1016/j.bbapap.2009.04.012.

102. Löbel, D., J. Strotmann, …, H. Breer. 2001. Identification of a Third Rat Odorant-binding Protein (OBP3). *Chem. Senses.* 26:673–680. https://doi.org/10.1093/chemse/26.6.673.

103. Ramoni, R., F. Vincent, …, M. Tegoni. 2001. The Insect Attractant 1-Octen-3-ol Is the Natural Ligand of Bovine Odorant-binding Protein. *J. Biol. Chem.* 276:7150–7155. https://doi.org/10.1074/jbc.M010368200.

104. Vincent, F., R. Ramoni, …, C. Cambillau. 2004. Crystal structures of bovine odorant-binding protein in complex with odorant molecules. *Eur. J. Biochem.* 271:3832–3842. https://doi.org/10.1111/j.1432-1033.2004.04315.x.

105. Ramoni, R., S. Bellucci, …, S. D'Auria. 2007. The protein scaffold of the lipocalin odorant-binding protein is suitable for the design of new biosensors for the detection of explosive components. *J. Phys. Condens. Matter.* 19:395012. https://doi.org/10.1088/0953-8984/19/39/395012.

106. Campanacci, V., J. Krieger, …, M. Tegoni. 2001. Revisiting the specificity of Mamestra brassicae and Antheraea polyphemus pheromone-binding proteins with a fluorescence binding assay. *J. Biol. Chem.* 276:20078–20084. https://doi.org/10.1074/jbc.M100713200.

107. Katre, U. V., S. Mazumder, …, S. Mohanty. 2009. Ligand Binding Turns Moth Pheromone-binding Protein into a pH Sensor. *J. Biol. Chem.* 284:32167–32177. https://doi.org/10.1074/jbc.M109.013383.

108. Loebel, D., A. Scaloni, …, P. Pelosi. 2000. Cloning, post-translational modifications, heterologous expression and ligand-binding of boar salivary lipocalin. *Biochem. J.* 350:369–379.

109. Paliwal, A., and P. K. De. 2007. Purification, cloning and regulation of a novel acid-lipase-like protein of hamster expressed in lacrimal glands and tears during lactation. *Biochim. Biophys. Acta Mol. Cell Biol. Lipids.* 1771:55–65. https://doi.org/10.1016/j.bbalip.2006.10.002.

110. Kannan, S., P. Mareeswaran, …, B. F. Nytingel. 2011. Properties of mouse vomeronasal receptor and assessment of its role in pheromone signalling. *Rapid Commun. Mass Spectrom.* 25:262–270. https://doi.org/10.1002/rcm.4864.

111. Cui, L., K. Kittipongpittaya, …, E. A. Decker. 2014. Impact of Phosphoethanolamine Reverse Micelles on Lipid Oxidation in Bulk Oils. *J. Americ. Oil Chem. Soc.* 91:1931–1937. https://doi.org/10.1007/s11746-014-2544-9.

Biophysical Journal, Volume *125*

Supplemental information

Biophysical insights into a cryptic ligand site in the hydrophobic core of human PCNA

Alexis N. Dispensa, Sharon N. Greenwood, Jason Yang, Daniel E. Logatto, James Fusco, E. Railey White, Roderic G. Eckenhoff, Zhiwei Liu, and Brian P. Weiser

Table S1. Previously reported proteins that interact with 1-aminoanthracene (AMA). ND = not determined.

Protein	K_d for AMA binding	AMA Binding Site Information	References
Serum albumin (*B. taurus*)	18 µM	ND	(1)
α-Acid glycoprotein (*H. sapiens*)	6 µM	ND	(2)
Apo-ferritin (*E. caballus*)	70-96 µM	Binding site shared with general anesthetics and lipids	(3–5)
β-lactoglobulin (*B. taurus*)	3 µM	ND	(2)
$GABA_A$ receptors (*H. sapiens*)	ND	Binding site shared with etomidate (β3 homopentamer); AMA potentiates GABA response (α1β2γ2L)	(3, 6)
Gastric triacylglycerol lipase (*C. familiaris*; *H. sapiens*)	8 µM	Binding site shared with β-octyl glucoside and is conformation-specific	(7, 8)
Leukocyte function-associated antigen-1 (*H. sapiens*)	ND	Binding site shared with lovastatin and general anesthetics	(9, 10)
Lipocalin 11 (*R. norvegicus*)	2 µM	ND	(11)
Odorant binding proteins (*S. scrofa*; *R. norvegicus*)	1-5 µM	Binding site shared with odorants, small lipophilic molecules, and general anesthetics	(2, 12–18)
Pheromone binding proteins (*M. brassicae*; *A. Polyphemus*)	1-5 µM	Binding site shared with pheromones, small lipophilic molecules, and fatty acids	(19, 20)
Salivary lipocalin (*S. scrofa*)	9-12 µM	Binding site shared with odorants and small molecules	(21)
Sirtuin isoform 2 (*H. sapiens*)	4-37 µM	Binding site shared with propofol and lipid-like molecules; affinity is conformation-dependent	(22)
Tear acid lipase-like protein (*M. auratus*)	12 µM	ND	(23)
Tubulin (*B. taurus*)	ND	AMA binds colchicine site and inhibits tubulin polymerization	(24)
Vomeronasal type 2 receptor (*M. musculus*)	ND	Binding site shared with pheromonal odorant 2-octanamine	(25)

Figure S1. UV-Vis absorbance spectra of AMA dissolved in various solvents. These spectra were collected using 1 cm pathlength cuvettes using a ThermoFisher nanodrop instrument in cuvette mode. The solvent alone was recorded as the background and subtracted from the 30 µM AMA measurement.

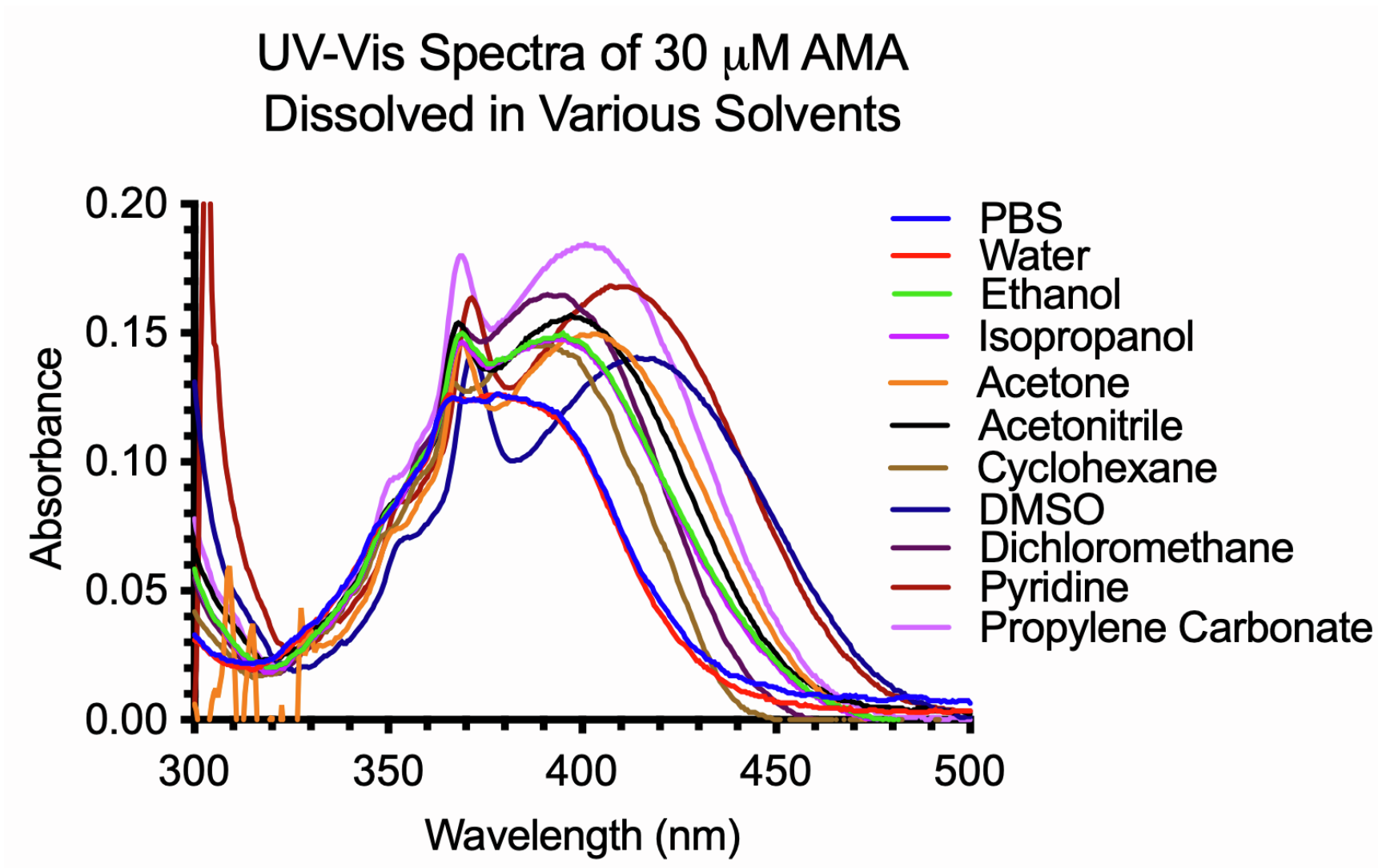

Figure S2. Fluorescence of 100 nM AMA in PBS with different concentrations of DOPC. DOPC was originally dissolved in ethanol, and the final concentration of ethanol in the cuvette was always 0.5%. For reference, the concentration of DOPC where it forms bilayers is ~50 µM (analogous to its critical micellar concentration) (26).

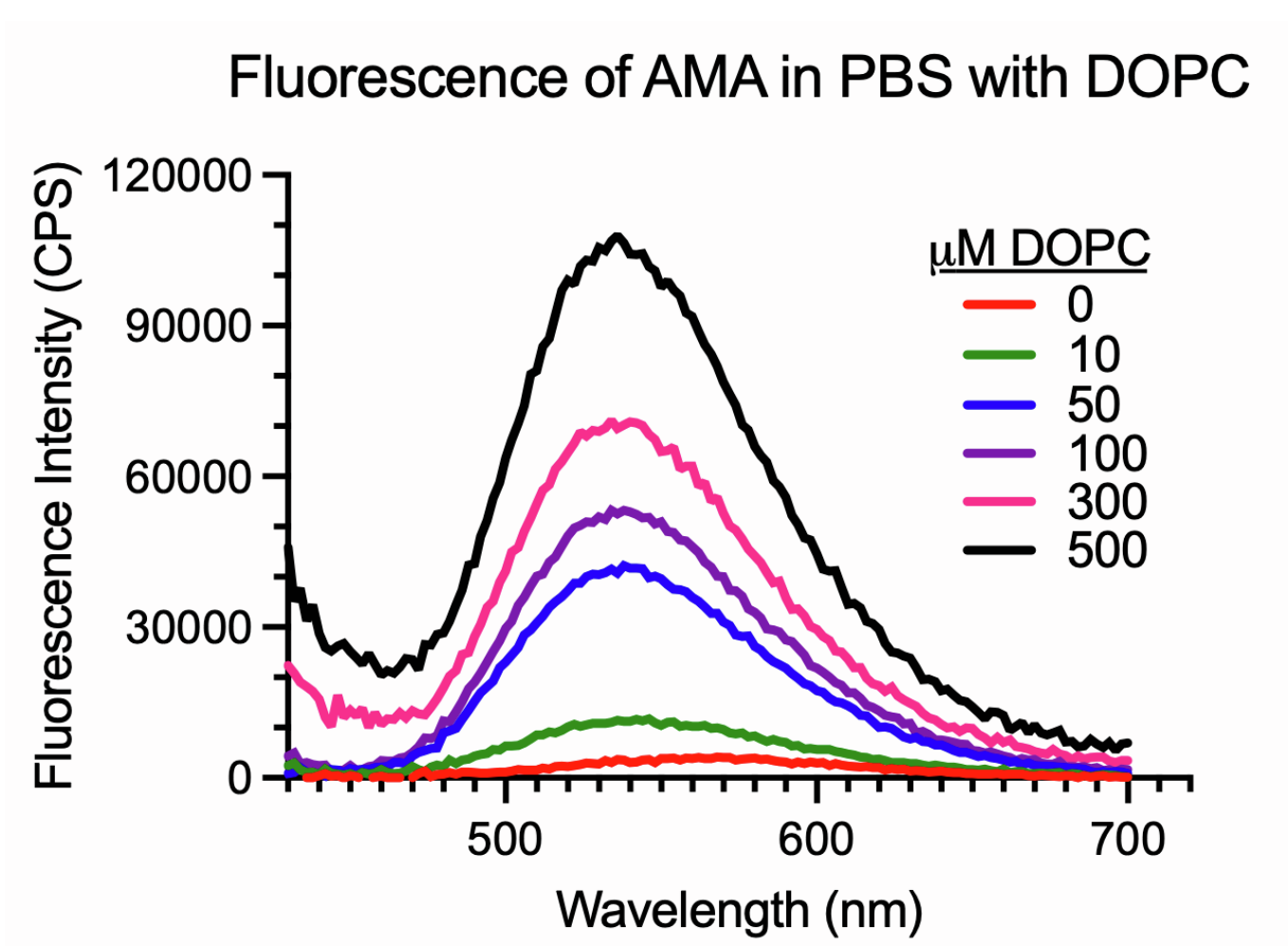

Figure S3. Anesthetics do not compete with AMA for ApoE or pyruvate kinase binding. (A) The fluorescence of 100 nM AMA is shown with 5 μM ApoE and the indicated ligands. (B) The fluorescence of 100 nM AMA is shown with 10 μM pyruvate kinase and the indicated ligands.

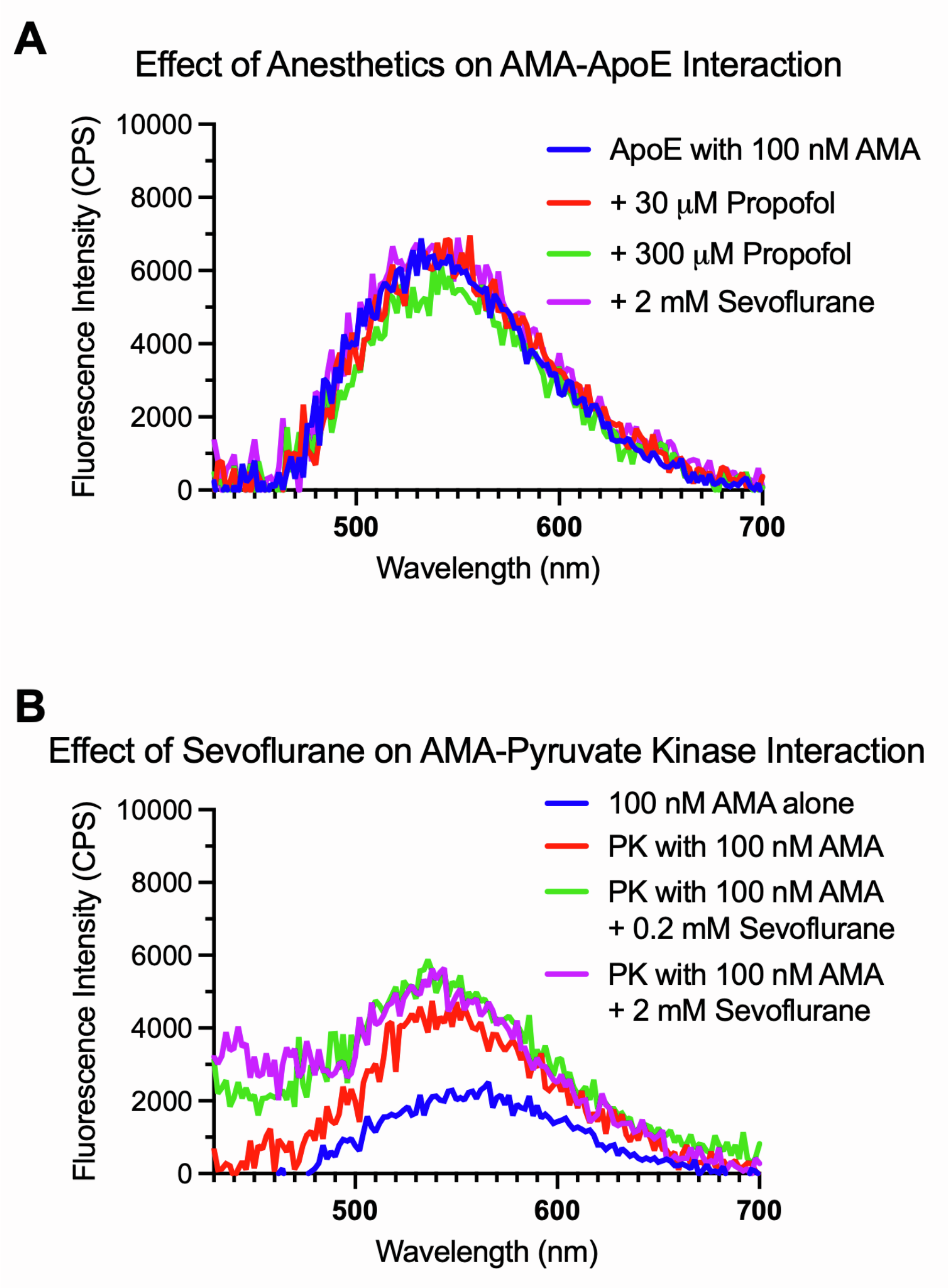

Figure S4. Mass spectra of peptides photolabeled by AziP*m*.

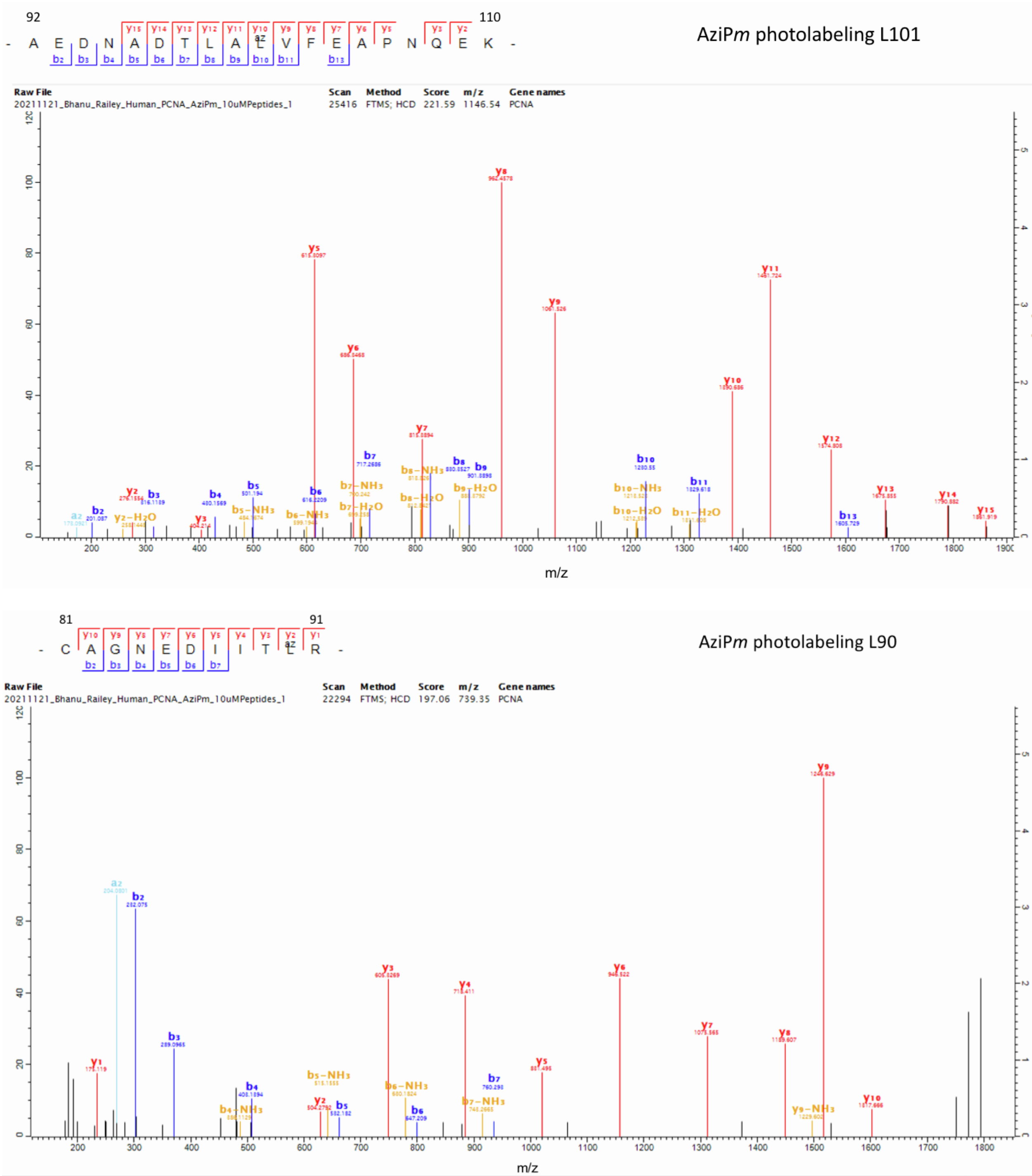

Figure S4. (continued)

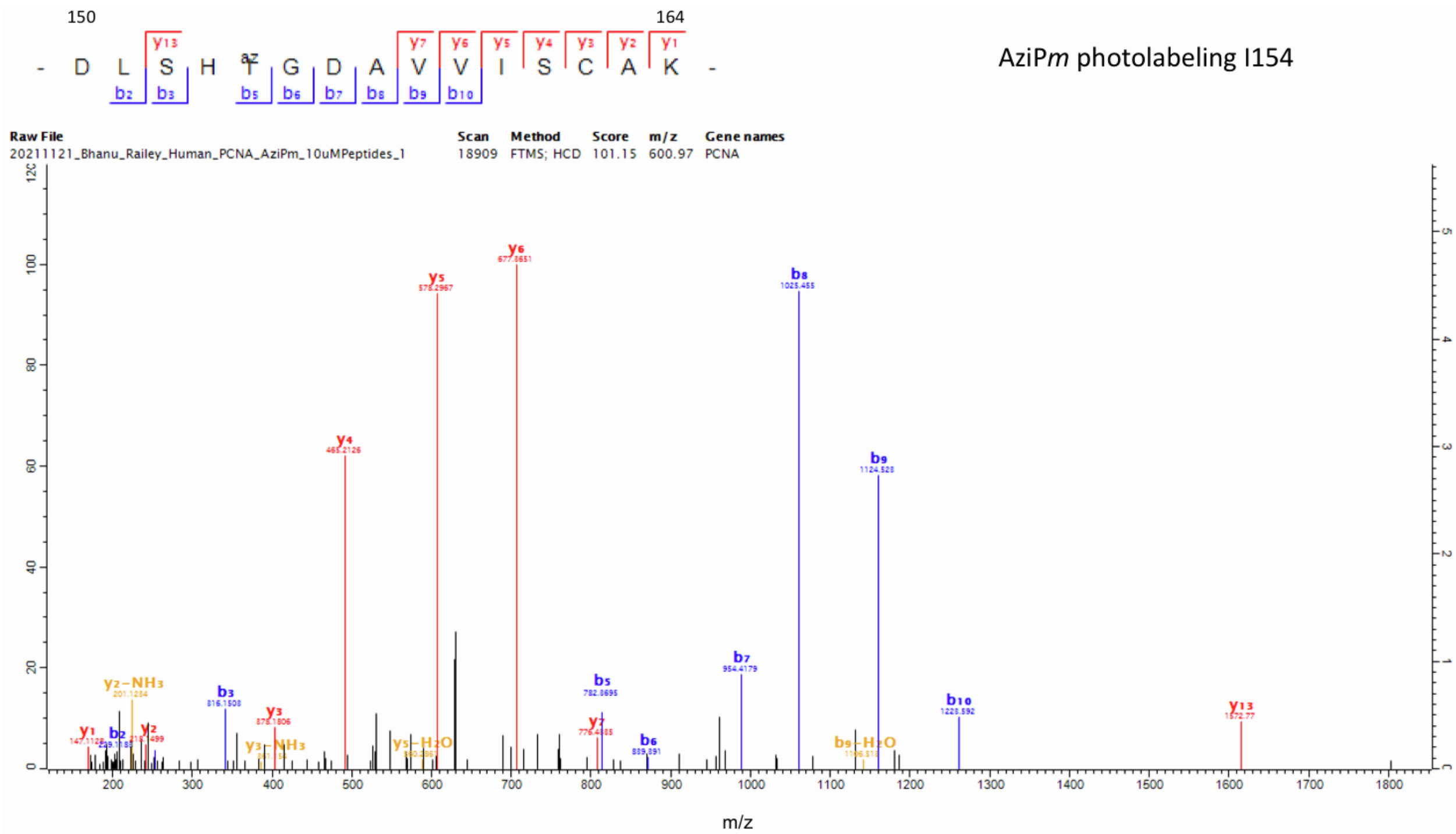

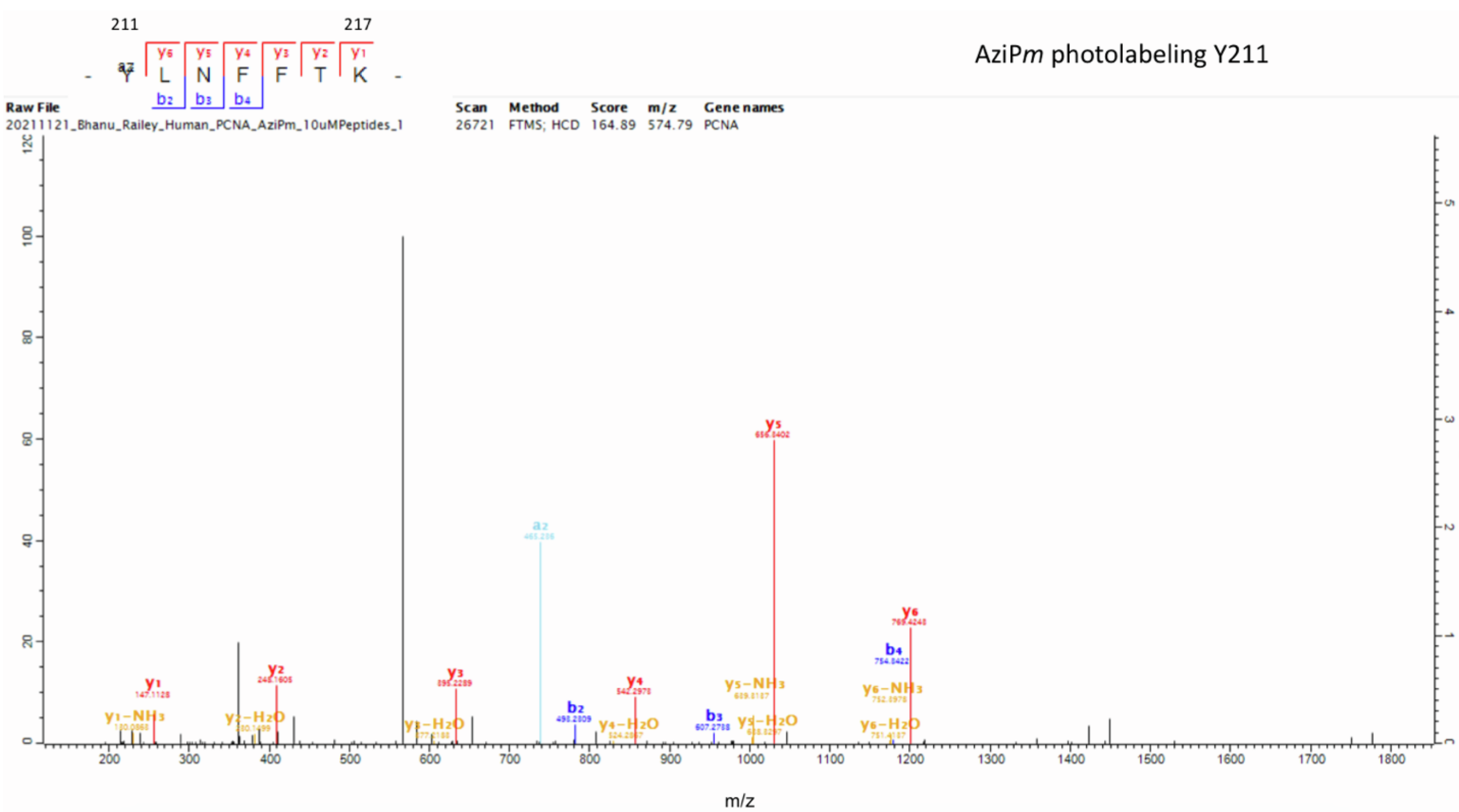

Figure S5. Surface cavity mutations that did not significantly affect AMA binding to PCNA. Y250 and V236 were in surface pockets identified by Fpocket as potential sites for small molecules, but their mutation did not significantly affect AMA binding or its emission peak wavelength.

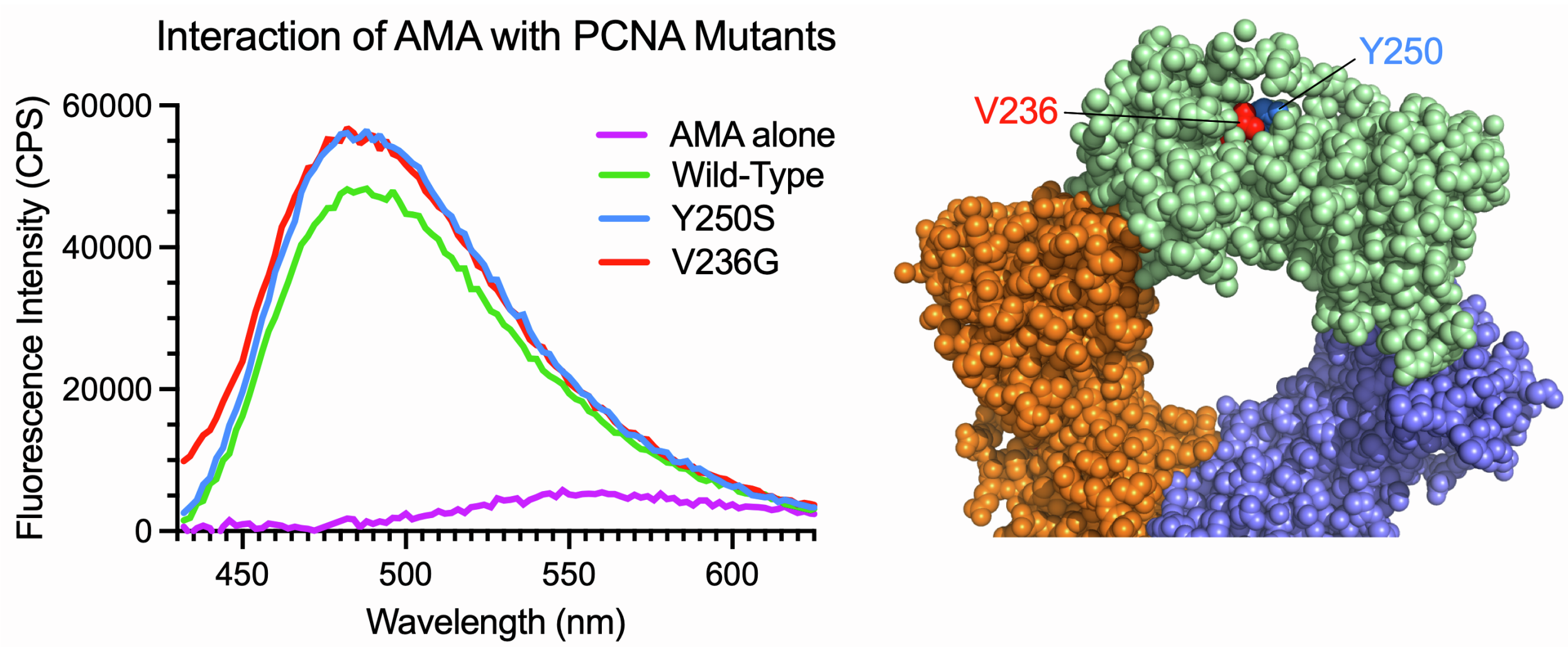

Figure S6. Fpocket analysis of the PCNA crystal structure PDB: 1VYM showing identified cavities in black and residues L90 and L101 as magenta and cyan sticks, respectively. No pockets or cavities were contacting these residues.

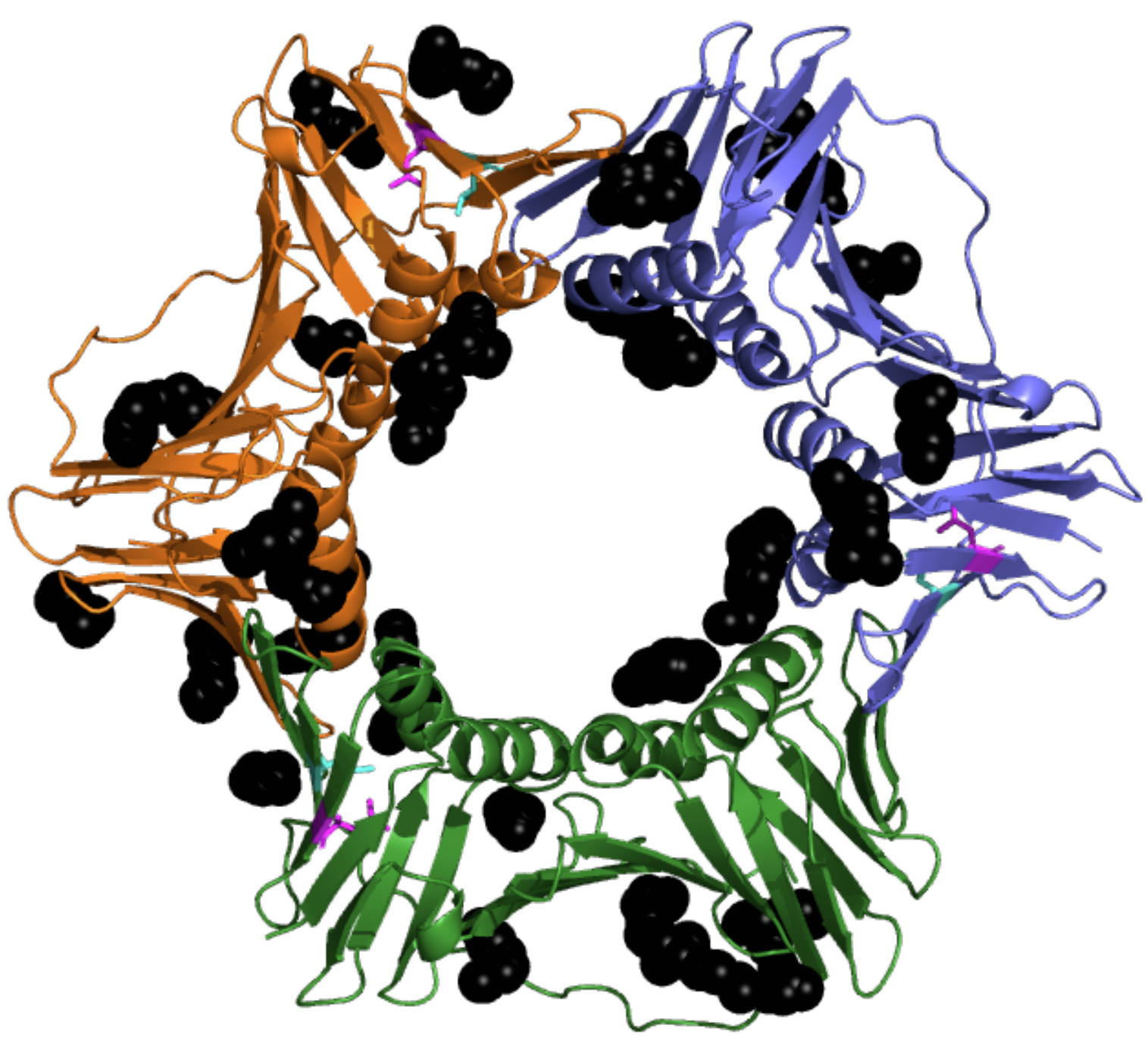

Figure S7. Root mean square deviation (RMSD) of protein backbone atoms throughout atomistic molecular dynamics simulations. The frames were referenced to the initial structure that resembled the crystal structure (PDB: 1VYM) for the 1 μs simulations, or AlphaFold-generated models for the 0.5 μs simulations.

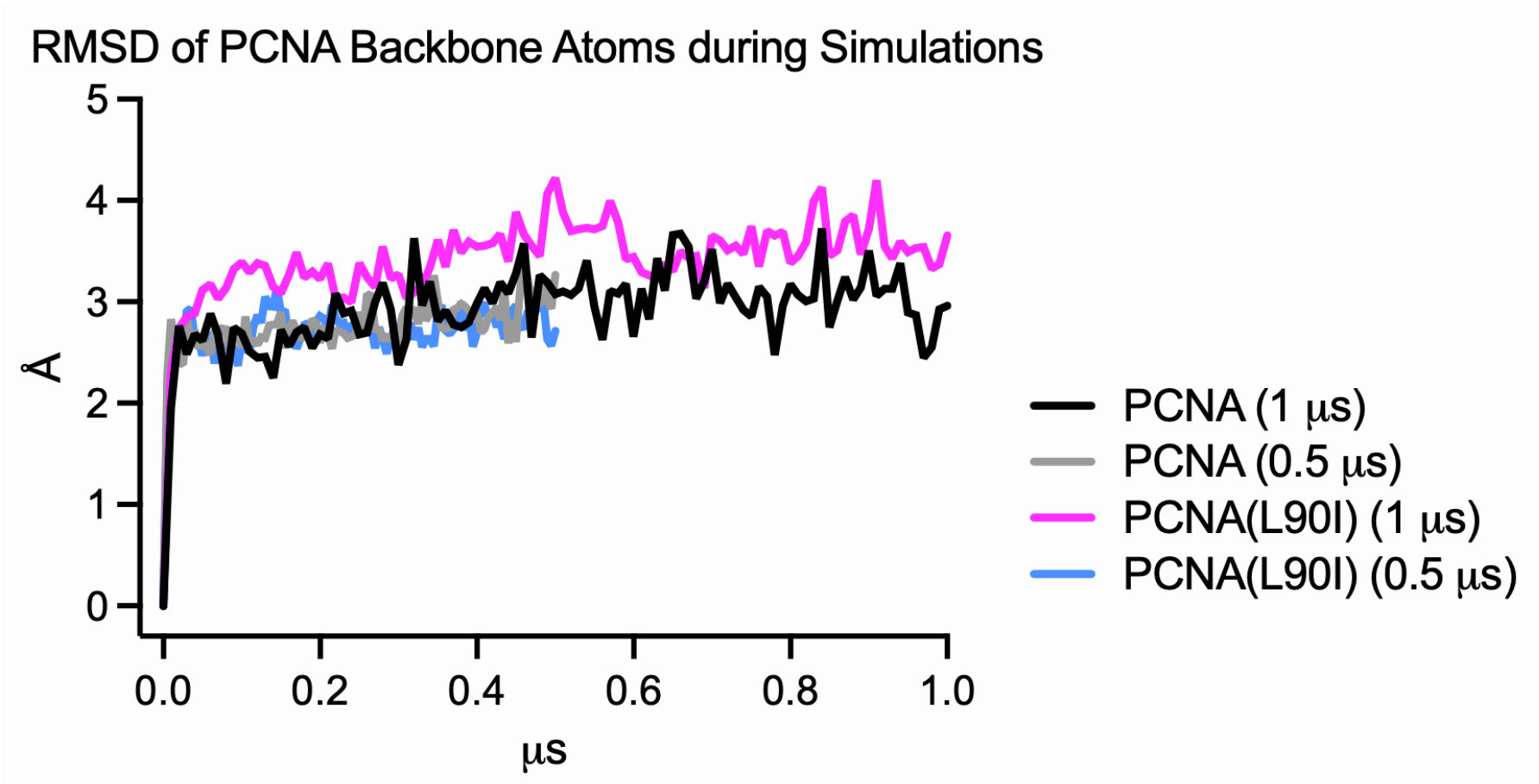

Table S2. Cryptic pocket analysis of wild-type PCNA and PCNA(L90I) from MD simulations. MDpocket analysis of 1 μs simulations was used to identify residues contributing to the cryptic site in subunits 1, 2, and 3 of the PCNA homotrimer. The list of pocket-lining residues were reported below.

Subunit	PCNA Variant	Cryptic Pocket Residues
1	WT	F2, A4, L6, L12, L16, W28, I30, V35, N36, L50, L52, F57, Y60, M68, V70, M75, L79, L90, L99, L101, L118
2	WT	F2, A4, L6, I11, L12, V15, L16, W28, I30, V35, L37, L50, L52, F57, M68, V70, M75, L79, I88, L90, R91, A92, L99, L101, L118
3	WT	F2, A4, L6, Q8, I11, L12, L16, W28, I30, V35, L50, L52, F57, Y60, M68, V70, M75, L79, I88, L90, R91, L99
1	L90I	F2, A4, L12, V15, L16, W28, I30, V35, L50, L52, F57, Y60, L66, M68, V70, L72, S74, M75, I78, L79, I88, I90, A92, L99, L101, M116, K117, L118, L175 from chain B
2	L90I	F2, A4, L6, L12, V15, L16, W28, D29, I30, S31, G34, V35, N36, L50, L52, F57, Y60, M68, V70, M75, L79, I88, I90, L99, L101, L118
3	L90I	F2, A4, L6, Q8, I11, L12, K13, V15, L16, L19, W28, I30, V35, N36, L37, L50, T51, L52, F57, Y60, M68, V70, L72, M75, L79, I88, T89, I90, A92, L99, L101, L118, L221, I241, M244

Figure S8. Pocket volumes in PCNA subunits over time and protein molecular interactions in the cryptic site. (A) Volume of the cryptic site in each of the three PCNA subunits throughout two independent MD simulations. (B) Volume of the cryptic site in each of the three PCNA(L90I) subunits throughout two independent MD simulations. Spearman rank correlations showed no relationship between pocket volumes in different subunits of the same PCNA homotrimer for both wild-type and PCNA(L90I) proteins. Spearman rank correlations were calculated in GraphPad Prism for two subunits at a time (data was non-parametric because pockets < ~25 $Å^3$ were not detected and assigned a volume of 0). (C) Volume of the cryptic site for every analyzed frame in MD simulations. The data for each of the three subunits were combined within the same simulation, and the black bar represents the median (*left* to *right*, 120.55, 136.06, 119.54, 137.57). (D) Top PCNA residue side chains that interacted with L90 or I90 in MD simulations of wild-type PCNA or PCNA(L90I). Each protein had three subunits. Values represent the percent of simulation time that each residue side chain was within 4 Å of L90 or I90. For statistical comparison, values were grouped by interacting residue (e.g., n = 6 per protein for residue 2). Data were analyzed using a two-way ANOVA followed by Sidak's multiple comparisons test in GraphPad Prism. Residues 2, 75, and 99 showed increased interaction with L90 compared to I90 (adjusted p value = 0.0001, 0.0064, and < 0.0001, respectively).

Figure S8. (continued)

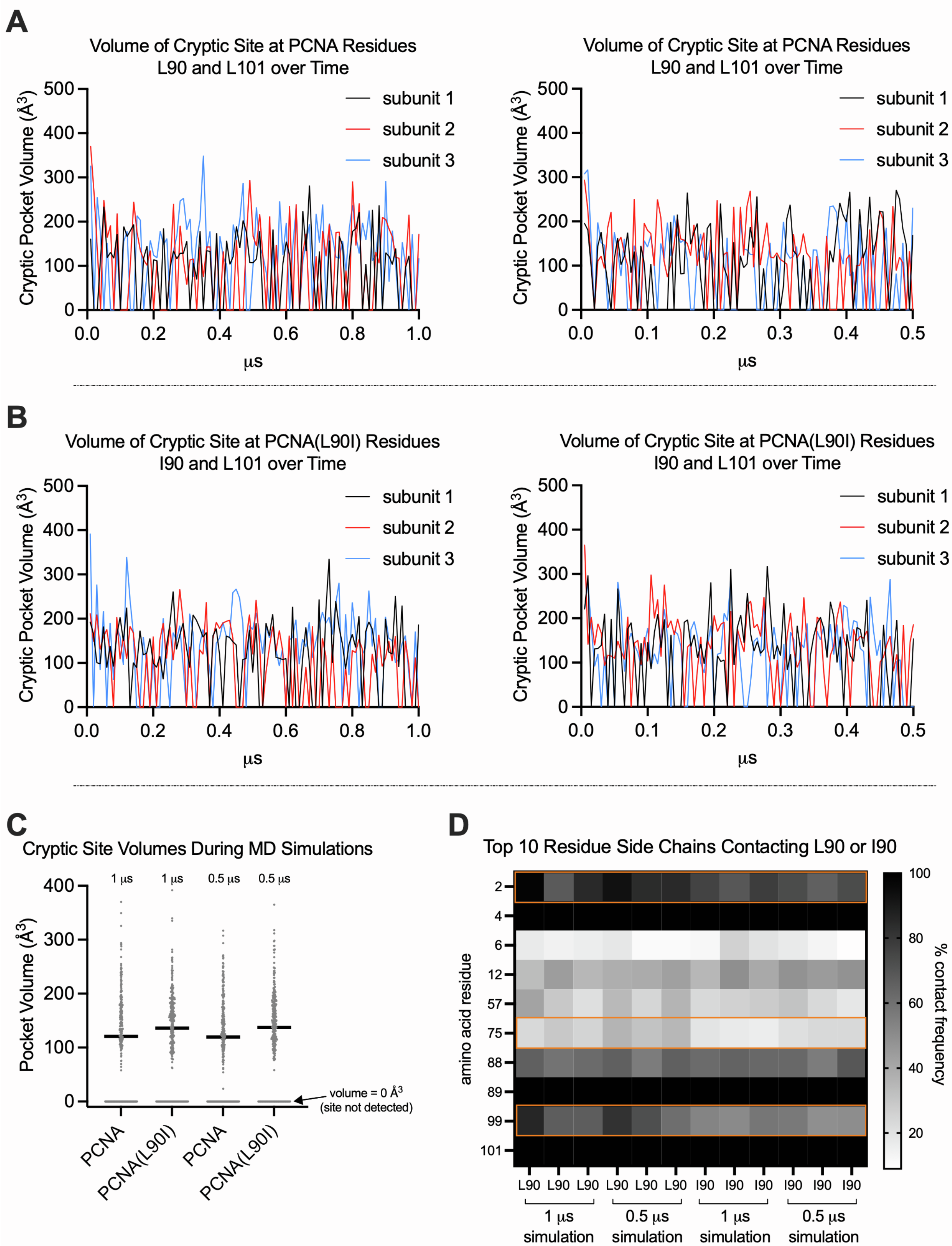

Figure S9. Ligand RMSD during MD simulations and snapshots of AMA unbinding PCNA(L90I). (A) RMSD of three sevoflurane molecules, each bound to a PCNA subunit for 1 μs. RMSD was calculated relative to the starting position of the ligand after aligning the protein backbone throughout the trajectory. Note that RMSD factors ligand translation, rotation, and internal conformational changes. Subunit 1 data is also shown in Video S1. (B) RMSD of three sevoflurane molecules, each bound to a PCNA(L90I) subunit for 1 μs. (C) RMSD of one AMA molecule bound to PCNA or PCNA(L90I) from three separate simulations. The PCNA data is also shown in Video S2. Snapshots from the AMA unbinding trajectory are shown in panel D. (D) Snapshots of AMA unbinding PCNA(L90I). Residues I90 and L101 in the cryptic site are shown as sticks along with residues V70, N71, S74, M116, K117, and M119 at the site exit point; residue L175 is also shown at the exit and is on a different PCNA subunit colored green. The gray blobs were pockets identified by MDpocket on the frames using default parameters. The third PCNA subunit was omitted from the background for clarity. Note that the ligand did not leave through the subunit interface, but instead through an intra-subunit tunnel that formed between I90/L90 and the indicated residues.

Figure S9. (continued)

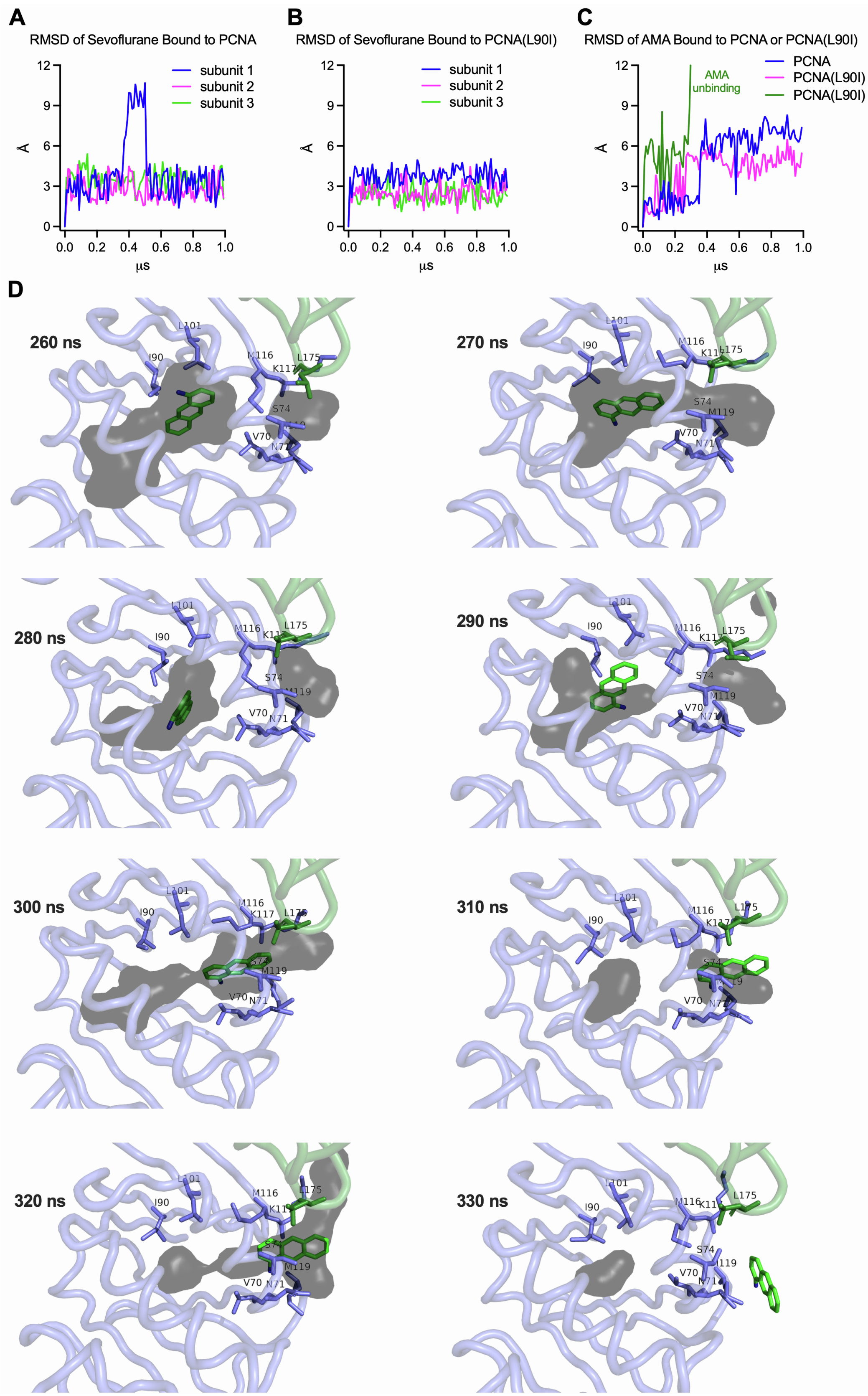

Figure S10. Thermostability of PCNA, PCNA(L90I), PCNA(C148S), or UNG2 in the absence and presence of sevoflurane. (A) The thermal unfolding of 3 µM PCNA protein was measured by recording its intrinsic tryptophan fluorescence at increasing temperatures (3 min holds every 2.5°C increment). When added, the sevoflurane concentration was 2.5 mM, and the DMSO vehicle was always 1%. The curves were fit using a four parameter sigmoidal function (see main text). (B) The thermal unfolding of 3 µM PCNA(L90I) conducted as in panel A. (C) The thermal unfolding of 3 µM UNG2 (uracil DNA glycosylase), which was conducted as above and fit with the four parameter function. For UNG2, we had to constrain the Y_{max} and Y_{min} parameters to 100 and 0, respectively, in order to determine the T_m values. (D) The thermal unfolding of 3 µM PCNA(C148S) conducted as in panel A. (E) Binding isotherm showing the interaction of AMA with recombinant PCNA(C148S). (F) Displacement of AMA from 10 µM PCNA(C148S) by sevoflurane. In this assay, 2 mM sevoflurane displaced 46% of AMA; as a close comparison, we determined that a concentration of 2.2 mM sevoflurane displaces 50% of AMA from wild-type PCNA (see Figure 3 in the main text).

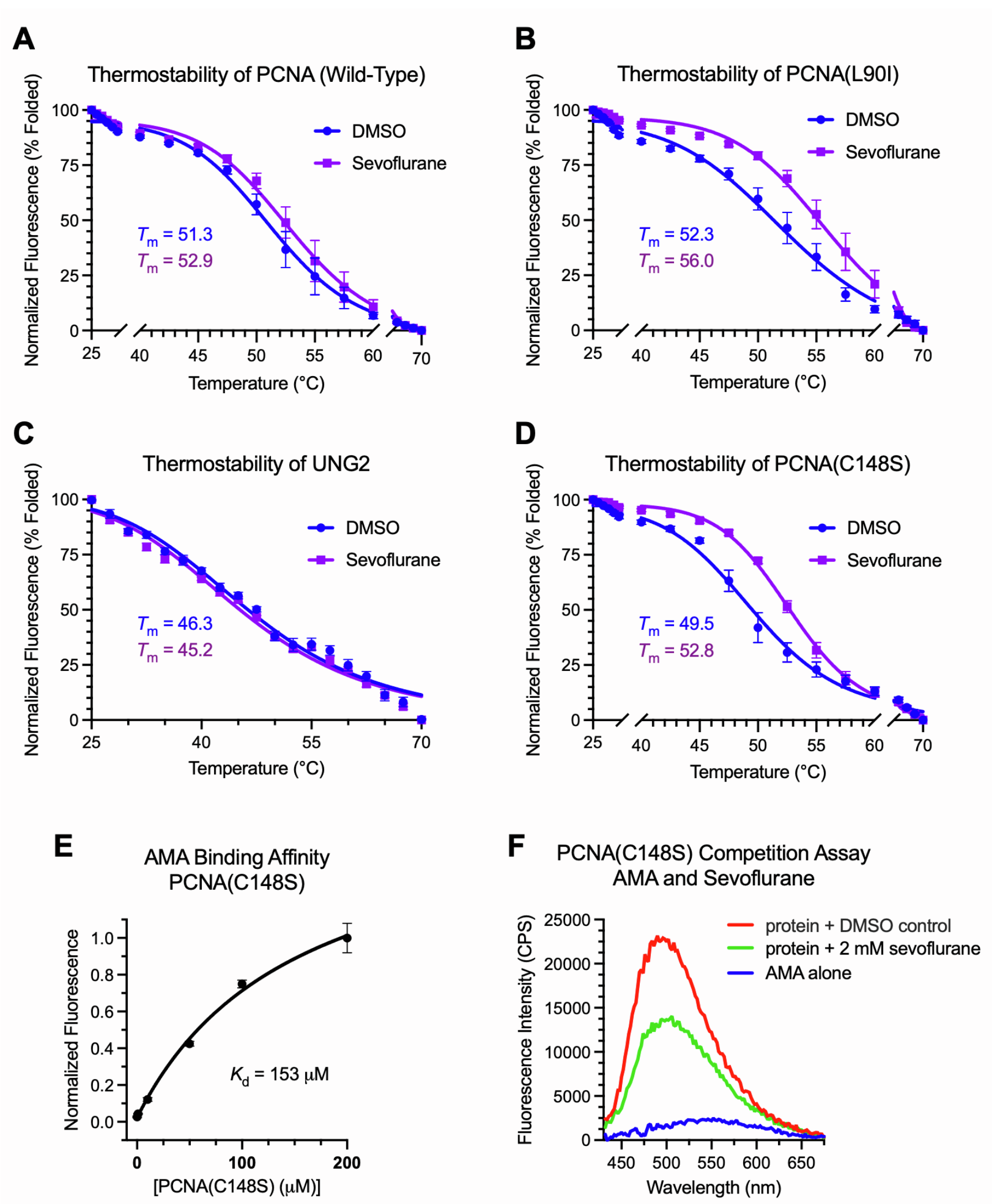

Figure S11. Anesthetic binding to the PCNA cryptic site does not affect interactions at the interdomain connecting loop (IDCL) site. (A) Sevoflurane did not significantly disrupt the interaction between PCNA and a fluorescein-labeled full-length UNG2 protein called UNG2(fluor) (27). In this assay, 50 nM UNG2(fluor) was complexed with 5 μM PCNA. If sevoflurane prevented their interaction, the fluorescence anisotropy of fluorescein-labeled UNG2 would reduce toward the free UNG2(fluor) baseline (27). (B) Propofol did not efficiently disrupt binding between PCNA and fluorescein-labeled UNG2. This experiment was conducted as in panel A. (C) The affinity (K_d) of PCNA for a fluorescein-labeled peptide called Pogo-Ligase was determined in the presence and absence of 100 μM propofol, which did not significantly affect the protein-peptide interaction. Methods for this assay can be found elsewhere (27, 28). (D) The affinity (K_d) of PCNA for a fluorescein-labeled p15 peptide was determined in the presence and absence of 2 mM sevoflurane, which did not significantly affect the protein-peptide interaction. This fluorescence anisotropy assay was performed with the same method as the Pogo-Ligase binding assay (27, 28), except that we used a peptide purchased from Biosynth with a p15 sequence instead (6FAM-dPEG4-AGGNPVCVRPTPKWQKGIGEFFRLS).

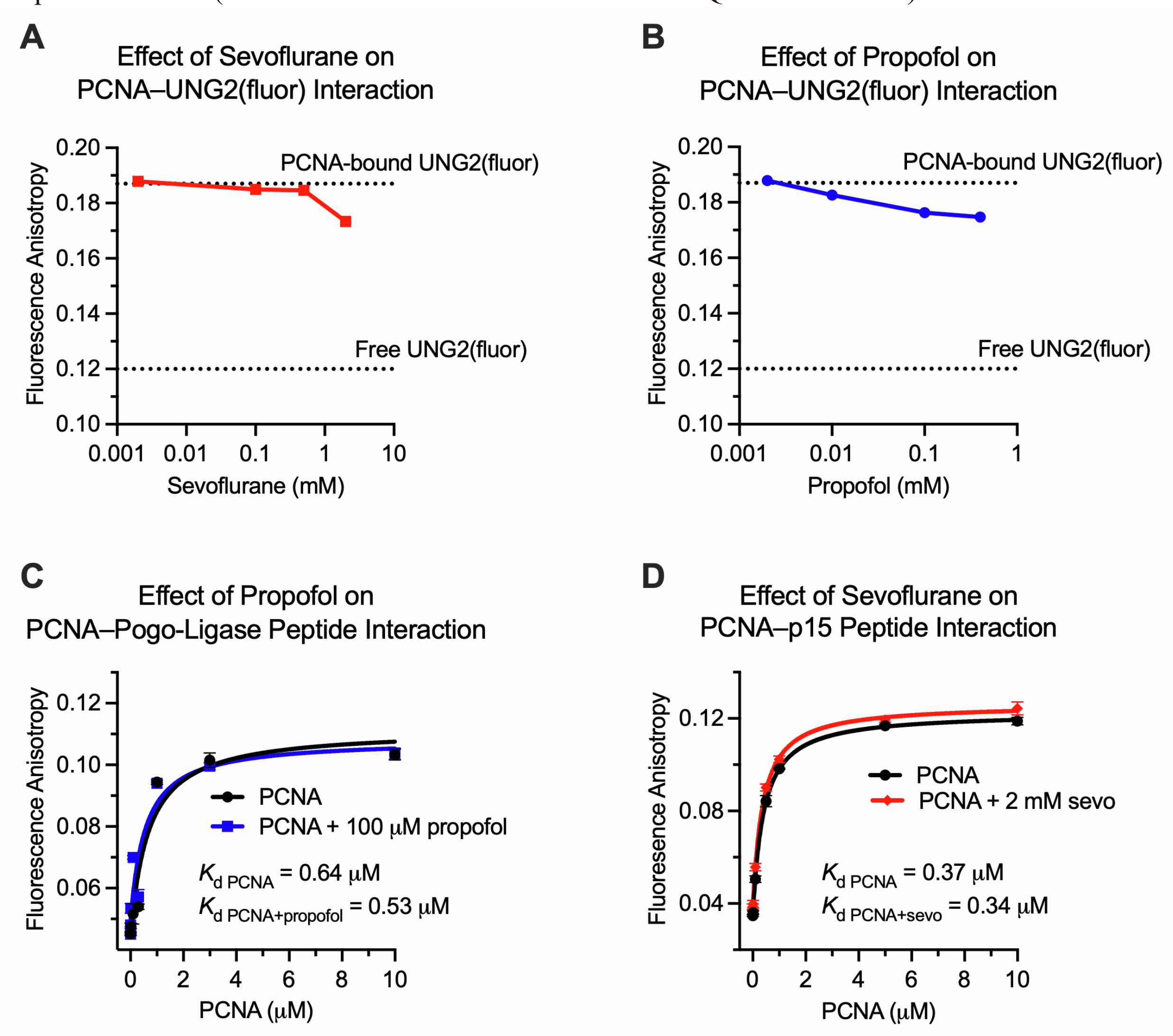

Figure S12. Sevoflurane did not affect loading of PCNA onto DNA by RFC. (A) Continuous timepoint fluorescence data from RFC-catalyzed ATP hydrolysis assays, which coupled ATP hydrolysis to NADH oxidation, which was observed by fluorescence (see Methods beneath the figure). 150 nM RFC was stimulated by 1 μM primer-template DNA, even more so in the presence of 1 μM PCNA, as previously reported (29). DMSO vehicle and 2.5 mM sevoflurane did not have a significant effect on ATP hydrolysis rate (i.e., the slope of the traces in panel A). (B) Rate constants (k_{obs}) for RFC activity from the ATP hydrolysis assays shown in panel A.

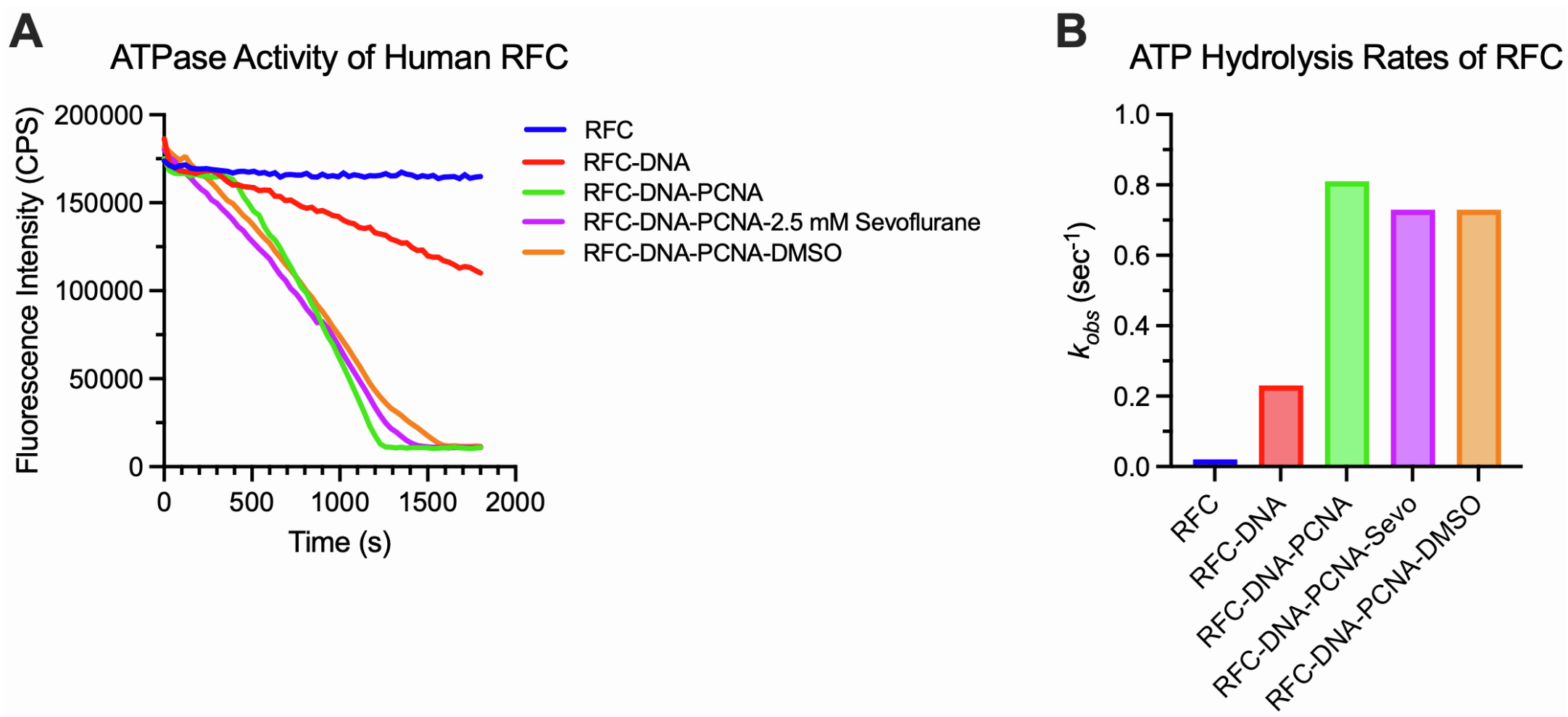

RFC-catalyzed ATP hydrolysis was monitored using an NADH-coupled enzyme assay that links ATP hydrolysis to NADH oxidation via pyruvate kinase and lactate dehydrogenase (29). Recombinant human ΔN555-RFC was a generous gift from Dr. Brian Kelch (University of Massachusetts). Reactions were performed in assay buffer containing 50 mM Tris (pH 7.5), 5 mM $MgCl_2$, 500 μM TCEP, 200 mM potassium glutamate, 1 mM ATP, 670 μM phosphoenolpyruvate, 170 μM NADH, 3 U/mL pyruvate kinase, and 3 U/mL lactate dehydrogenase. Reactions (200 μL, 23°C) containing 1 μM PCNA and 1 μM primer-template DNA oligonucleotide were initiated by addition of 150 nM RFC. NADH fluorescence was measured continuously (λ_{ex} = 350 nm, λ_{em} = 460 nm) using a Horiba Fluoromax-4 instrument. The decrease in NADH fluorescence corresponds stoichiometrically to ATP hydrolysis and was plotted against time (s) to calculate observed rates (K_{obs}) of RFC activity using the equations

$$Rate = \frac{m * [NADH]}{Y_{max} - Y_{min}} \qquad Rate\ Constant\ (k_{obs}) = \frac{Rate}{[RFC]}$$

where m was the slope obtained from a simple linear regression fit of the data in GraphPad Prism, [NADH] was the initial NADH concentration, Y_{max} was the maximum values of NADH fluorescence intensity in the absence of RFC, Y_{min} was the minimum plateau after complete ATP hydrolysis by RFC, and [RFC] was the RFC concentration in the assay.

<u>Primer-template oligonucleotide sequences (annealed for the assays)</u>
5'-ATCGAGCTGGACACCATCACTCCATATTGGTCACTTAGCTATCGATTCCCGCATGC
TCTTAGCTATCGATTCACCTGAGACTAGTCA-3'

5'-TGACTAGTCTCAGGTGAATCGAUAGCTAAGAGCATGCGGGAATCGAU
AGCTAAGT-3'

Figure S13. Relationship between pocket volumes at the L90/L101 site and the resolution of experimental data. (A) A correlation was observed between subunit pocket volumes and structural resolution for human PCNA structures. (B) No correlation was observed between subunit pocket volumes and structural resolution for yeast PCNA structures. Spearman rank correlations were calculated in GraphPad Prism.

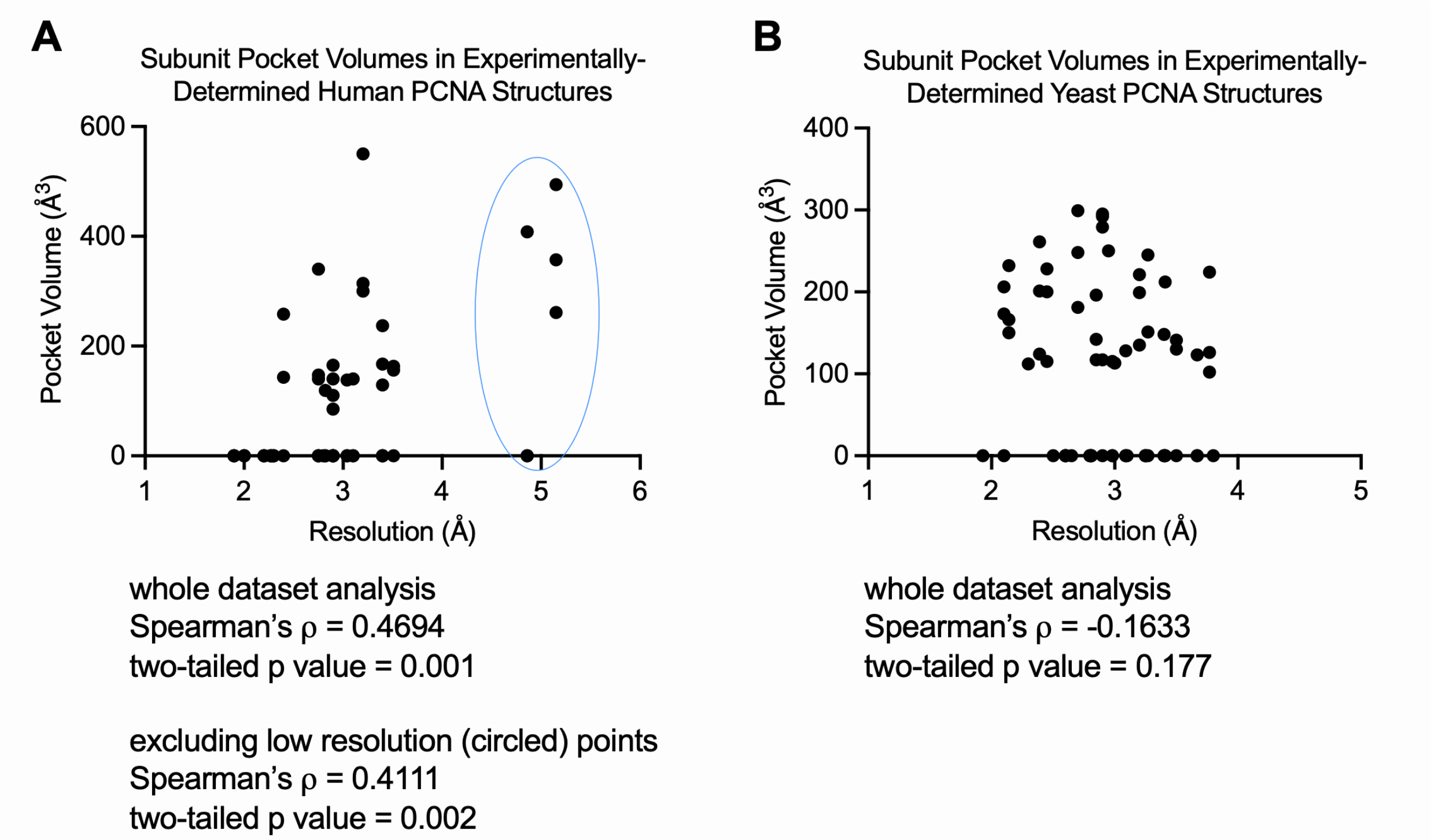

Table S3. Hydrophobic pocket volumes at sites analogous to the PCNA L90/L101 site in various PCNA and β clamp proteins.

PDB	Description	Chain A	Chain B	Chain C	Link
		**Pocket Volume (Angstroms^3)** (not detected assigned value of 0; blank = less than 3 subunits in asymmetric unit)			
**human structures**					
1VYM	Native human crystal; Xray; 2.3 angstroms; apo	0	0	0	https://doi.org/10.1073/pnas.0406540102
6GWS	Human PCNA bound to three p15 peptides; xray; 2.9 angstroms	110	0	0	https://doi.org/10.1093/nar/gky723
6EHT	Human PCNA bound to two p15 peptides and dsDNA; xray, 3.2 angstroms	300	550	314	https://doi.org/10.1093/nar/gky723

5MOM	Human PCNA-S228I ; mutant with IDCL impaired binding and impaired DNA repair; xray; 2.27 angstroms	0	0	0	https://doi.org/10.1016/j.dnarep.2016.12.003
6GIS	Human PCNA bound to dsDNA; xray, 2.82 angstroms	0	119	0	https://doi.org/10.1038/ncomms13935
3TBL	Human monoubiquinated PCNA ; 2 ubiquitins; xray; 2.9 angstroms	140	0	165	https://doi.org/10.4161/cc.20595
1UL1	Human PCNA bound to three FEN1 proteins; xray 2.9 angstroms	0	85	0	https://doi.org/10.1038/sj.emboj.7600519
3WGW	Human PCNA bound to small molecule IDCL inhibitor; xray 2.8	0			https://doi.org/10.1074/jbc.M113.520429
8E84.	Human PCNA C148S; xray 3.10	0	0	140	https://doi.org/10.1016/j.jbc.2023.104656
8UII	Cryo-EM of human clamp-clamp loader ATAD5-RFC-closed PCNA complex in intermediate state 1; cryo-EM 3.04	138	0	0	https://doi.org/10.1038/s41594-024-01332-4
6S1N	Human polymerase delta holoenzyme Conformer 2; cryo-EM 4.86	408	0	0	https://doi.org/10.1038/s41467-020-14898-6
6VVO	Structure of the human clamp loader (Replication Factor C, RFC) bound to the sliding clamp; cryo-EM 3.40	167	0	0	https://doi.org/10.1101/2020.02.18.953257
7KQ0	human PCNA bound to peptide mimetic; xray 2.40	143	258	0	https://doi.org/10.1016/j.jbc.2021.100773
8YJZ	Structure of the human endogenous PCNA-FEN1-RNase H2 complex - State D; cryo-EM 5.15	261	494	357	https://doi.org/10.1038/s44318-024-00296-x

6CBI	PCNA in complex with inhibitor; xray 2.75	0	0	0	https://doi.org/10.1002/chem.201801734
4RJF	Crystal structure of the human sliding clamp at xray 2.0 angstrom resolution	0	0	0	https://doi.org/10.1021/acs.biochem.5b00241
8UMV	human CTF18-RFC-PCNA-DNA ternary complex with narrow PCNA opening state I (state 5); cryo-EM 2.75	340	140	147	https://doi.org/10.1073/pnas.2319727121
5YCO	Complex structure of PCNA with UHRF2; xray 2.2	0	0	0	https://doi.org/10.1016/j.bbrc.2017.09.102
7NV0	Human Pol Kappa holoenzyme with wt PCNA; cryo-EM 3.4	237	0	129	https://doi.org/10.1038/s41467-021-26251-6
8F5Q	Crystal structure of human PCNA in complex with the PIP box of FBH1; xray 1.9	0	0	0	https://doi.org/10.1016/j.str.2023.03.004
8YJL	Structure of the human endogenous PCNA-FEN1 complex - State B; cryo-EM 3.51	163	156	0	https://doi.org/10.1038/s44318-024-00296-x
**yeast structures**					-
8GLA	Co-crystal structure of caPCNA bound to the AOH1996 derivative, AOH1996-1LE; xray 3.77	224	102	126	https://doi.org/10.1016/j.chembiol.2023.07.001
8GL9	Co-crystal structure of caPCNA bound to AOH1160 derivative 1LE; xray 2.81	0	0	0	https://doi.org/10.1016/j.chembiol.2023.07.001
1PLQ	apo yeast PCNA; xray 2.3	112			https://doi.org/10.1016/0092-8674(94)90014-0
2OD8	yeast PCNA bound to CDC9 PIP peptide; saccharomycese cerevisiae;xray 2.8	0			https://doi.org/10.1093/nar/gkm006
1SXJ	yeast PCNA bound to yeast RFC (no DNA), PCNA ring closed;	117	142	196	https://doi.org/10.1038/nature02585

	saccharomycese cerevisiae; xray 2.85				
7TFH	yeast PCNA bound to yeast RFC and DNA; PCNA ring closed; saccharomycese cerevisiae; cryo EM 3.09	0	0	128	https://doi.org/10.7554/eLife.77469
7TI8	yeast PCNA bound to RFC only, no DNA, ring open; saccharomycese cerevisiae; cryo EM 3.5	0	130	141	https://doi.org/10.7554/eLife.74175
7TIB	yeast PCNA ring open bound to RFC with primer-template DNA; saccharomycese cerevisiae; cryo EM 3.4	0	148	0	https://doi.org/10.7554/eLife.74175
8DQX	open state of RFC:PCNA bound to a 3' ss/dsDNA junction; saccharomycese cerevisiae; cryo EM 2.1	0	206	173	https://doi.org/10.7554/eLife.78253
3GPN	non-trimeric form of the yeast E113G PCNA mutant protein; saccharomycese cerevisiae; xray 2.5	0			https://doi.org/10.1107/S0907444909011329
3F1W	yeast PCNA mutant G168S; mutant blocks translesion synthesis; saccharomycese cerevisiae; xray 2.9	117			https://doi.org/10.1021/bi8017762
7TFI	yeast PCNA bound to yeast RFC and DNA; PCNA ring open; saccharomycese cerevisiae; cryo EM 3.41	0	212	0	https://doi.org/10.7554/eLife.77469
5V7M	PCNA mutant L126A/I128A Protein Defective in Gene Silencing; Saccharomyces cerevisiae S288C; xray 1.93	0			https://doi.org/10.1371/journal.pone.0193333

5T9D	Structure of PCNA acetylated on K20; Saccharomyces cerevisiae S288C; xray 3.27	245	151	0	https://doi.org/10.2210/pdb5T9D/pdb
3K4X	Saccharomyces cerevisiae Sliding Clamp PCNA Bound to DNA; xray 2.98	115	0	0	https://doi.org/10.1186/1472-6807-10-3
8THW	Cac1 PIP motif bound to Saccharomyces cerevisiae PCNA; xray 2.6	0	0	0	https://doi.org/10.1016/j.jmb.2024.168695
3V60	Structure of S. cerevisiae PCNA conjugated to SUMO on lysine 164; xray 2.6	0			https://doi.org/10.1038/nature10883
3L0W	Structure of split monoubiquitinated PCNA with ubiquitin in position two; xray 2.8	0			https://doi.org/10.1038/nsmb.1776
3V62	S. cerevisiae Srs2 C-terminal domain in complex with PCNA conjugated to SUMO on lysine 164; xray 2.9	0			https://doi.org/10.1038/nature10883
8THC	Structure of the Saccharomyces cerevisiae clamp unloader Elg1-RFC bound to a cracked PCNA; cryo-EM 3.67	123	0	0	https://doi.org/10.1126/sciadv.adl1739
8THD	Structure of the Saccharomyces cerevisiae clamp unloader Elg1-RFC bound to PCNA; cryo-EM 3.25	0	0	0	https://doi.org/10.1126/sciadv.adl1739
8TWB	Structure of S. cerevisiae Ctf18-RFC-PCNA-DNA complex; cryo-EM 3.2	199	221	135	https://doi.org/10.1126/science.adk5901
8DR1	Consensus closed state of RFC:PCNA bound to a 3' ss/dsDNA junction (DNA2); xray 2.14	232	150	166	https://doi.org/10.7554/eLife.78253

8DR4	Open state of RFC:PCNA bound to a 3' ss/dsDNA junction (DNA2) without NTD; cryo-EM 2.45	228	200	115	https://doi.org/10.7554/eLife.78253
8DR6	Closed state of RFC:PCNA bound to a nicked dsDNA; cryo-EM 2.39	124	201	261	https://doi.org/10.7554/eLife.78253
8DR7	Open state of RFC:PCNA bound to a nicked dsDNA; cryo-EM 2.7	181	248	299	https://doi.org/10.7554/eLife.78253
4YHR	Crystal Structure of Yeast Proliferating Cell Nuclear Antigen; Saccharomyces cerevisiae S288C; xray 2.95	250			https://doi.org/10.2210/pdb4YHR/pdb
6XC3	S179T Mutant of Saccharomyces cerevisiae S288C PCNA; xray 3.10	0			https://doi.org/10.1371/journal.pone.0157023
3L0X	Structure of split yeast PCNA; Saccharomyces cerevisiae; xray 3.0	113			ttps://doi.org/10.1038/nsmb.1776
6W9W	R80A PCNA mutant defective in BIR; Saccharomyces cerevisiae; xray 2.65	0			https://doi.org/10.2210/pdb6W9W/pdb
3GPM	Structure of the trimeric form of the E113G PCNA mutant protein; Saccharomyces cerevisiae; xray 3.8	0			https://doi.org/10.1107/S0907444909011329
6QH1	The structure of Schizosaccharomyces pombe PCNA in complex with an Spd1 derived peptide; xray 2.9	279	295	292	https://doi.org/10.1093/nar/gkae011
**prokaryotic B clamps**					
2HIK	apo archael heterotrimeric PCNA; Sulfolobus solfataricus; xray 3.3	0	0	0	https://doi.org/10.1016/j.molcel.2006.08.015

3IFV	Haloferax volcanii archael PCNA; xray 2.0	179	153	174	https://doi.org/10.1186/1472-6807-9-55
1RWZ	Archaeoglobus fulgidus archael PCNA; xray 1.8	315			https://doi.org/10.1016/s0092-8674(03)01036-5
1RXM	Archaeoglobus fulgidus archael PCNA bound to C-terminal region of FEN-1 peptide; xray 2.8	198			https://doi.org/10.1016/s0092-8674(03)01036-5
2NTI	Saccharolobus solfataricus P2 PCNA heterotrimer (archael); xray 2.5	171	0	0	https://doi.org/10.1107/S0907444908021665
3AIX	Sulfurisphaera tokodaii str. 7; xray 2.9; archael; PCNA2-PCNA3 complex from Sulfolobus tokodaii	72	0		https://doi.org/10.1016/j.jsb.2011.02.006
1RXZ	archael; xray 2.0; C-terminal region of A. fulgidus FEN-1 complexed with A. fulgidus PCNA	263			https://doi.org/10.1016/s0092-8674(03)01036-5
7RPX	archael; Archaeal DNA ligase and heterotrimeric PCNA in complex with end-joined DNA; cryo EM 4.2;	359	562	288	https://doi.org/10.1016/j.str.2021.11.002
1GE8	PROLIFERATING CELL NUCLEAR ANTIGEN (PCNA) HOMOLOG FROM PYROCOCCUS FURIOSUS; xray 2.1	281			https://doi.org/10.1110/ps.36401
8PPT	Pyrococcus abyssi DNA polymerase D (PolD) in editing mode bound to P/T substrate containing a mismatch; cryo-EM 2.9	309	0	0	https://doi.org/10.1038/s41467-023-44125-x
3LX1	Crystal Structure analysis of PCNA1 from Thermococcus kodakaraensis tk0535; xray 2.0	102			https://doi.org/10.1073/pnas.1019179108
2IX2	Crystal structure of the heterotrimeric	165	153	101	https://doi.org/10.1107/S1744309106034075

	PCNA from Sulfolobus solfataricus; xray 2.2				
7RPW	Archaeal DNA ligase and heterotrimeric PCNA in complex with adenylated DNA; cryo-EM 4.38	492	612	758	https://doi.org/10.1016/j.str.2021.11.002
5A6D	Proliferating Cell Nuclear Antigen, PCNA, from Thermococcus gammatolerans; xray 2.8	219	244		https://doi.org/10.1016/j.bbrep.2016.08.004
7N5M	PCNA from Thermococcus gammatolerans: crystal III, collection 1, 2.00 A, 1.91 MGy	198	199		https://doi.org/10.1002/prot.26346
6T7Y	Structure of PCNA bound to cPIP motif of DP2 from P. abyssi; xray 2.7	278			https://doi.org/10.1038/s41467-020-15392-9
6T8H	Structure of the DNA-bound PolD-PCNA processive complex from P. abyssi; cryo-EM 3.77	179	174	0	https://doi.org/10.1038/s41467-020-15392-9
4TSZ	bacterial beta-clamp; DNA polymerase sliding clamp from Pseudomonas aeruginosa with ligand; xray 2.0	139	117		https://doi.org/10.1021/jm500467a
6AP4	Crystal structure of the DNA polymerase III subunit beta from Acinetobacter baumannii; 2.95 angstroms	0	0		https://doi.org/10.1016/j.jsb.2018.10.008
7AZD	DNA polymerase sliding clamp from Escherichia coli with peptide 20 bound; xray 2.19	0	0		https://doi.org/10.1021/acs.jmedchem.1c00918
3BEP	E. coli beta clamp bound to DNA; xray 1.92	0	0		https://doi.org/10.1016/j.cell.2007.11.045

7EVP	Cryo-EM structure of the staph aureus Gp168-beta-clamp complex; cryo-em 3.2	0	0	https://doi.org/10.1093/nar/gkab875
8VAL	cryo em Structure of the E. coli clamp loader bound to the beta clamp in a Open-DNAp/t conformation; 3.7 angstroms	0	0	https://doi.org/10.1016/j.jbc.2024.107166
8VAQ	cryo em Structure of the E. coli clamp loader bound to the beta clamp in a Closed-DNA1 conformation; 3.8 angstroms	146	133	https://doi.org/10.1016/j.jbc.2024.107166
4N97	E. coli sliding clamp in complex with 5-nitroindole; xray 1.97	0	0	https://doi.org/10.1021/jm500122r
6FVM	Mutant DNA polymerase sliding clamp from Escherichia coli with bound P7 peptide; xray 1.631	0	0	https://doi.org/10.1021/acsinfecdis.9b00089
6AMS	Crystal structure of the DNA polymerase III subunit beta from Pseudomonas aeruginosa	182	233	https://doi.org/10.1016/j.jsb.2018.10.008
4TR7	Crystal structure of DNA polymerase sliding clamp from Mycobaterium tuberculosis; xray 2.29	208	127	https://doi.org/10.1021/jm500467a
3D1G	Structure of a small molecule inhibitor bound to a DNA sliding clamp; xray 1.64	106	161	https://doi.org/10.1073/pnas.0804754105
6E8E	Crystal structure of the Escherichia coli sliding clamp-MutL complex; xray 2.25	0	0	https://doi.org/10.1093/nar/gkz115
1UNN	Complex of beta-clamp E. coli processivity factor and little finger	0	0	https://doi.org/10.1093/emboj/cdg568

	domain of PolIV; xray 1.9				
8GIY	E. coli clamp loader with closed clamp; cryo-EM 3.7	283	291		https://doi.org/10.1038/s41467-024-52623-9
**AlphaFold3 structures**					
wild-type	PCNA model 1	102.918	0	102.735	
wild-type	PCNA model 2	0	0	0	
wild-type	PCNA model 3	0	0	0	
wild-type	PCNA model 4	0	0	0	
wild-type	PCNA model 5	0	0	0	
Mutant	PCNA(L90I) model 1	0	0	0	
Mutant	PCNA(L90I) model 2	0	0	0	
Mutant	PCNA(L90I) model 3	112.322	106.443	108.049	
Mutant	PCNA(L90I) model 4	111.586	125.478	105.367	
Mutant	PCNA(L90I) model 5	417.289	434.512	306.777	
**The structures below were not included in Figure 6D (main text) because of their unique or unusual hydrophobic site features which we did not characterize in detail (more than one pocket in the same pseudodomain, pockets in adjacent pseudodomains, etc., as described below)**					
6J8Y	Crystal structure of the human RAD9-HUS1-RAD1-RHINO complex, a PCNA-like DNA clamp; xray 2.4	104	409, 156 (on adjacent pseudodomains)	125	https://doi.org/10.1074/jbc.AC119.011816
9EOA	Human PCNA bound to dsDNA; xray, 2.82 angstroms	ND	two adjacent pockets; 250 angstroms closest to L90 and 164 angstroms closest to L101	248 angstroms	https://doi.org/10.1038/s41467-025-59324-x

9B8T	Human polymerase epsilon bound to PCNA and DNA in the nucleotide bound state; cryo-EM 2.95 angstroms	ND	240	two adjacent pockets near 90; 247 and 229 angstroms	https://doi.org/10.1038/s41467-024-52257-x
7TFI	yeast PCNA bound to yeast RFC and DNA; PCNA ring open; saccharomycese cerevisiae; cryo EM 3.41	ND	2 pockets; 114 and 98 angstroms	ND	https://doi.org/10.7554/eLife.77469
8UI8	Cryo-EM map of human clamp-clamp loader ATAD5-RFC-two PCNAs complex in intermediate state 3; cryo-EM 3.10	136, 146, 212 (PCNA dimer w. 3 adjacent internal pockets on one subunit)	0		https://doi.org/10.1038/s41594-024-01332-4
6TNY	Processive human polymerase delta holoenzyme; cryo-EM 3.08	238, 400 (on opposing pseudodomains)	0	0	https://doi.org/10.1038/s41467-020-14898-6
6TNZ	Human polymerase delta-FEN1-PCNA toolbelt; cryo-EM 4.05	0	238, 389 (adjacent pseudodomains)	0	https://doi.org/10.1038/s41467-020-14898-6
9NEA	Human POL epsilon bound to PCNA and DNA with a pre-existing mismatch in the blocked conformation II; cryo-EM 3.81	638,826 (adjacent pseudodomains)	148, 533 (adjacent pseudomains)	111, 322 (adjacent pseudodomains)	https://doi.org/10.1073/pnas.2507232122
9NE9	Human POL epsilon bound to PCNA and DNA with a pre-existing mismatch in the blocked conformation I; cryo-EM 3.88	764, 303 (adjacent pseudodomains)	134, 817 (adjacent pseudodomains)		https://doi.org/10.1073/pnas.2507232122
5E0V	Human PCNA variant (S228I) complexed with FEN1 at xray 2.1 Angstroms	125, 100 (adjacent in same pseudodomain near L90)		150, 223 (adjacent pseudodomains)	https://doi.org/10.1016/j.jmb.2015.11.029
7KC0	Saccharomyces cerevisiae replicative polymerase delta in complex with a primer/template and	120, 87 (adjacent pseudodomains)	0	0	https://doi.org/10.1073/pnas.2017637117

	the PCNA clamp; cryo-EM 3.2				
8PPU	Pyrococcus abyssi DNA polymerase D (PolD) in editing mode bound to P/T substrate containing three consecutive mismatches; cryo-EM 3.02	265	205, 61 (adjacent in same pseudodomain)	0	https://doi.org/10.1038/s41467-023-44125-x
6AMQ	Crystal structure of the DNA polymerase III subunit beta from Enterobacter cloacae; xray 2.67	146, 151 (adjacent pseudomains)	0		https://doi.org/10.1016/j.jsb.2018.10.008
8GIZ	E. coli clamp loader with open clamp; cryo-EM 2. 7	97, 544 (adjacent pseudodomains)	409		https://doi.org/10.1038/s41467-024-52623-9

Supporting Material References

1. Hong, J.Y., J. Cassel, J. Yang, H. Lin, and B.P. Weiser. 2021. High-Throughput Screening Identifies Ascorbyl Palmitate as a SIRT2 Deacetylase and Defatty-Acylase Inhibitor. *ChemMedChem* 16:3484–3494, doi: 10.1002/cmdc.202100343.

2. Kmiecik, D., and J.R. Albani. 2010. Effect of 1-aminoanthracene (1-AMA) binding on the structure of three lipocalin proteins, the dimeric β lactoglobulin, the dimeric odorant binding protein and the monomeric α1-acid glycoprotein. Fluorescence spectra and lifetimes studies. *J Fluoresc* 20:973–983, doi: 10.1007/s10895-010-0643-z.

3. Butts, C.A., J. Xi, G. Brannigan, A.A. Saad, S.P. Venkatachalan, R.A. Pearce, M.L. Klein, R.G. Eckenhoff, and I.J. Dmochowski. 2009. Identification of a fluorescent general anesthetic, 1-aminoanthracene. *Proc. Natl. Acad. Sci. U.S.A.* 106:6501–6506, doi: 10.1073/pnas.0810590106.

4. Woll, K.A., B.P. Weiser, Q. Liang, T. Meng, A. McKinstry-Wu, B. Pinch, W.P. Dailey, W.D. Gao, M. Covarrubias, and R.G. Eckenhoff. 2015. Role for the propofol hydroxyl in anesthetic protein target molecular recognition. *ACS Chem Neurosci* 6:927–935, doi: 10.1021/acschemneuro.5b00078.

5. Bu, W., R. Liu, J.C. Cheung-Lau, I.J. Dmochowski, P.J. Loll, and R.G. Eckenhoff. 2012. Ferritin couples iron and fatty acid metabolism. *FASEB J.* 26:2394–2400, doi: 10.1096/fj.11-198853.

6. Miller, P.S., and A.R. Aricescu. 2014. Crystal structure of a human $GABA_A$ receptor. *Nature* 512:270–275, doi: 10.1038/nature13293.

7. Miled, N., L. Berti-Dupuis, M. Riviere, F. Carrière, and R. Verger. 2003. In vitro lipolysis by human pancreatic lipase is specifically abolished by its inactive forms. *Biochim Biophys Acta* 1645:241–246, doi: 10.1016/s1570-9639(02)00537-x.

8. Roussel, A., N. Miled, L. Berti-Dupuis, M. Rivière, S. Spinelli, P. Berna, V. Gruber, R. Verger, and C. Cambillau. 2002. Crystal Structure of the Open Form of Dog Gastric Lipase in Complex with a Phosphonate Inhibitor*. *Journal of Biological Chemistry* 277:2266–2274, doi: 10.1074/jbc.M109484200.

9. Yuki, K., W. Bu, J. Xi, M. Shimaoka, and R. Eckenhoff. 2013. Propofol shares the binding site with isoflurane and sevoflurane on leukocyte function-associated antigen-1. *Anesth. Analg.* 117:803–811, doi: 10.1213/ANE.0b013e3182a00ae0.

10. Bu, W., L.M. Pereira, R.G. Eckenhoff, and K. Yuki. 2014. Stereoselectivity of Isoflurane in Adhesion Molecule Leukocyte Function-Associated Antigen-1. *PLOS ONE* 9:e96649, doi: 10.1371/journal.pone.0096649.

11. Gu, Y., Q. Liu, P. Chen, C. Guo, Y. Liu, Y. Zhao, Y. Zhang, and D. Lin. 2013. Characterization of the oligomerization and ligand-binding properties of recombinant rat lipocalin 11. *Biochim Biophys Acta* 1834:1–7, doi: 10.1016/j.bbapap.2012.08.018.

12. Paolini, S., F. Tanfani, C. Fini, E. Bertoli, and P. Pelosi. 1999. Porcine odorant-binding protein: structural stability and ligand affinities measured by fourier-transform infrared spectroscopy and fluorescence spectroscopy. *Biochim. Biophys. Acta* 1431:179–188.

13. Johansson, J.S., G.A. Manderson, R. Ramoni, S. Grolli, and R.G. Eckenhoff. 2005. Binding of the volatile general anesthetics halothane and isoflurane to a mammalian beta-barrel protein. *FEBS J.* 272:573–581, doi: 10.1111/j.1742-4658.2004.04500.x.

14. Meillour, P.N.-L., P. Lagant, J.-P. Cornard, F. Brimau, C. Le Danvic, G. Vergoten, and J.-C. Michalski. 2009. Phenylalanine 35 and tyrosine 82 are involved in the uptake and release of ligand by porcine odorant-binding protein. *Biochimica et Biophysica Acta (BBA) - Proteins and Proteomics* 1794:1142–1150, doi: 10.1016/j.bbapap.2009.04.012.

15. Löbel, D., J. Strotmann, M. Jacob, and H. Breer. 2001. Identification of a Third Rat Odorant-binding Protein (OBP3). *Chemical Senses* 26:673–680, doi: 10.1093/chemse/26.6.673.

16. Ramoni, R., F. Vincent, S. Grolli, V. Conti, C. Malosse, F.-D. Boyer, P. Nagnan-Le Meillour, S. Spinelli, C. Cambillau, and M. Tegoni. 2001. The Insect Attractant 1-Octen-3-ol Is the Natural Ligand of Bovine Odorant-binding Protein*. *Journal of Biological Chemistry* 276:7150–7155, doi: 10.1074/jbc.M010368200.

17. Vincent, F., R. Ramoni, S. Spinelli, S. Grolli, M. Tegoni, and C. Cambillau. 2004. Crystal structures of bovine odorant-binding protein in complex with odorant molecules. *European Journal of Biochemistry* 271:3832–3842, doi: 10.1111/j.1432-1033.2004.04315.x.

18. Ramoni, R., S. Bellucci, I. Grycznyski, Z. Grycznyski, S. Grolli, M. Staiano, G.D. Bellis, F. Micciulla, R. Pastore, A. Tiberia, V. Conti, E. Merli, A. Varriale, M. Rossi, and S. D'Auria. 2007. The protein scaffold of the lipocalin odorant-binding protein is suitable for the design of new biosensors for the detection of explosive components. *J. Phys.: Condens. Matter* 19:395012, doi: 10.1088/0953-8984/19/39/395012.

19. Campanacci, V., J. Krieger, S. Bette, J.N. Sturgis, A. Lartigue, C. Cambillau, H. Breer, and M. Tegoni. 2001. Revisiting the specificity of Mamestra brassicae and Antheraea polyphemus pheromone-binding proteins with a fluorescence binding assay. *J Biol Chem* 276:20078–20084, doi: 10.1074/jbc.M100713200.

20. Katre, U.V., S. Mazumder, R.K. Prusti, and S. Mohanty. 2009. Ligand Binding Turns Moth Pheromone-binding Protein into a pH Sensor. *J Biol Chem* 284:32167–32177, doi: 10.1074/jbc.M109.013383.

21. Loebel, D., A. Scaloni, S. Paolini, C. Fini, L. Ferrara, H. Breer, and P. Pelosi. 2000. Cloning, post-translational modifications, heterologous expression and ligand-binding of boar salivary lipocalin. *Biochem J* 350:369–379.

22. Bi, D., J. Yang, J.Y. Hong, P. Parikh, N. Hinds, J. Infanti, H. Lin, and B.P. Weiser. 2020. Substrate-Dependent Modulation of SIRT2 by a Fluorescent Probe, 1-Aminoanthracene. *Biochemistry* 59:3869–3878, doi: 10.1021/acs.biochem.0c00564.

23. Paliwal, A., and P.K. De. 2007. Purification, cloning and regulation of a novel acid-lipase-like protein of hamster expressed in lacrimal glands and tears during lactation. *Biochimica et Biophysica Acta (BBA) - Molecular and Cell Biology of Lipids* 1771:55–65, doi: 10.1016/j.bbalip.2006.10.002.

24. Emerson, D.J., B.P. Weiser, J. Psonis, Z. Liao, O. Taratula, A. Fiamengo, X. Wang, K. Sugasawa, A.B. Smith 3rd, R.G. Eckenhoff, and I.J. Dmochowski. 2013. Direct modulation of microtubule stability contributes to anthracene general anesthesia. *J. Am. Chem. Soc.* 135:5389–5398, doi: 10.1021/ja311171u.

25. Kannan, S., P. Mareeswaran, M. Krishnan, S. Achiraman, N. Spencer, and B.F. Nytingel. 2011. Properties of mouse vomeronasal receptor and assessment of its role in pheromone signalling. *Rapid Communications in Mass Spectrometry* 25:262–270, doi: 10.1002/rcm.4864.

26. Cui, L., K. Kittipongpittaya, D.J. McClements, and E.A. Decker. 2014. Impact of Phosphoethanolamine Reverse Micelles on Lipid Oxidation in Bulk Oils. *Journal of the American Oil Chemists' Society* 91:1931–1937, doi: 10.1007/s11746-014-2544-9.

27. Weiser, B.P., J.T. Stivers, and P.A. Cole. 2017. Investigation of N-Terminal Phospho-Regulation of Uracil DNA Glycosylase Using Protein Semisynthesis. *Biophys. J.* 113:393–401, doi: 10.1016/j.bpj.2017.06.016.

28. Greenwood, S.N., R.G. Belz, and B.P. Weiser. 2022. A Conserved Mechanism for Hormesis in Molecular Systems. *Dose Response* 20:15593258221109335, doi: 10.1177/15593258221109335.

29. Gaubitz, C., X. Liu, J. Magrino, N.P. Stone, J. Landeck, M. Hedglin, and B.A. Kelch. 2020. Structure of the human clamp loader reveals an autoinhibited conformation of a substrate-bound AAA+ switch. *Proc. Natl. Acad. Sci. U.S.A.* 117:23571–23580, doi: 10.1073/pnas.2007437117.
